# Separating the effects of life course adiposity on diabetic nephropathy: a comprehensive multivariable Mendelian randomization study

**DOI:** 10.3389/fendo.2024.1285872

**Published:** 2024-02-08

**Authors:** Han Zhang, QingYa Zhang, YiJue Song, LiJun Wang, MinChao Cai, JinFang Bao, Qing Yu

**Affiliations:** Department of Nephrology, Shanghai General Hospital, Shanghai Jiao Tong University School of Medicine, Shanghai, China

**Keywords:** Mendelian randomization, diabetic nephropathy, life course adiposity, childhood obesity, obesity

## Abstract

**Aims:**

Previous Mendelian randomization (MR) of obesity and diabetic nephropathy (DN) risk used small sample sizes or focused on a single adiposity metric. We explored the independent causal connection between obesity-related factors and DN risk using the most extensive GWAS summary data available, considering the distribution of adiposity across childhood and adulthood.

**Methods:**

To evaluate the overall effect of each obesity-related exposure on DN (Ncase = 3,676, Ncontrol = 283,456), a two-sample univariate MR (UVMR) analysis was performed. The independent causal influence of each obesity-related feature on DN was estimated using multivariable MR (MVMR) when accounting for confounding variables. It was also used to examine the independent effects of adult and pediatric obesity, adjusting for their interrelationships. We used data from genome-wide association studies, including overall general (body mass index, BMI) and abdominal obesity (waist-to-hip ratio with and without adjustment for BMI, i.e., WHR and WHR_adj_BMI), along with childhood obesity (childhood BMI).

**Results:**

UVMR revealed a significant association between adult BMI (OR=1.24, 95%CI=1.03-1.49, P=2.06×10^-2^) and pediatric BMI (OR=1.97, 95%CI=1.59-2.45, P=8.55×10^-10^) with DN risk. At the same time, adult WHR showed a marginally significant increase in DN (OR =1.27, 95%CI = 1.01-1.60, P=3.80×10^-2^). However, the outcomes were adverse when the influence of BMI was taken out of the WHR (WHR_adj_BMI). After adjusting for childhood BMI, the causal effects of adult BMI and adult abdominal obesity (WHR) on DN were significantly attenuated and became nonsignificant in MVMR models. In contrast, childhood BMI had a constant and robust independent effect on DN risk(adjusted for adult BMI: IVW, OR=1.90, 95% CI=1.60-2.25, P=2.03×10^-13^; LASSO, OR=1.91, 95% CI=1.65-2.21, P=3.80×10^-18^; adjusted for adult WHR: IVW, OR=1.80, 95% CI=1.40-2.31, P=4.20×10^-6^; LASSO, OR=1.90, 95% CI=1.56-2.32, P=2.76×10^-10^).

**Interpretation:**

Our comprehensive analysis illustrated the hazard effect of obesity-related exposures for DN. In addition, we showed that childhood obesity plays a separate function in influencing the risk of DN and that the adverse effects of adult obesity (adult BMI and adult WHR) can be substantially attributed to it. Thus, several obesity-related traits deserve more attention and may become a new target for the prevention and treatment of DN and warrant further clinical investigation, especially in childhood obesity.

## 1 Introduction

Diabetic nephropathy (DN), a severe microvascular complication associated with diabetes, is characterized by a decline in estimated glomerular filtration rate (eGFR) or the presence of albuminuria. DN is widely recognized as the primary factor behind end-stage renal disease (ESRD) globally (1), exacerbating the occurrence and progression of cardiovascular disease and mortality (2). Around 30% to 40% of people with diabetes will eventually develop DN (3). Furthermore, the financial load of DN and ESKD is immense (4). Therefore, it is vital to identify the potential causative factor for the prevention and management of DN.

It is well-known that obesity poses a unique risk for the onset and progression of DN (5). The main subtypes of obesity are general obesity and abdominal obesity. Consistent findings from conventional epidemiologic research have consistently shown an increased likelihood of DN associated with adult BMI (a measure of overall adiposity in adults). In contrast, the results for adult WHR (a measure of abdominal adiposity in adults) have been inconclusive (6–8).

In addition, while childhood obesity has been a concern for decades, it is now an undeniable public health crisis (9), with evidence from observational studies linking early-life obesity to a greater risk of chronic conditions such as type 2 diabetic mellitus (10–13). DN has a more extended latency period, and obese children are more likely than children with a standard body mass index (BMI) to develop end-stage renal disease (ESRD) or chronic kidney disease (CKD) (14). Hence, it is reasonable to suggest that the early stages of life, including childhood and adolescence, play a significant role in shaping adults’ susceptibility to DN through adiposity. Because of the inconsistent findings in observational research regarding the link between abdominal obesity and DN, as well as the lack of sufficient evidence regarding the connection between early-life adiposity and the risk of DN. Due to the complexity of these relationships, it is essential to understand how different obesity-related characteristics interact to increase the risk of DN.

Mendelian randomization (MR) employs genetic variations in the reproductive cell line as substitutes to enable causal deduction between a specific exposure and a result (15). Due to the random distribution and stable characteristics of genetic variations, In comparison to conventional observational analysis, MR analyses are anticipated to be less susceptible to conventional confounding and reverse causality (16).

Furthermore, multivariable MR enables the evaluation of distinct impacts of various exposures (e.g., childhood and adult BMI) on health outcomes (17–19). Univariable MR can estimate the general impact of body size in early life on DN (20, 21). However, independent of adult body size, multivariable MR can assess the precise impact of childhood obesity on DN risk. This approach has recently been used to investigate whether body size in childhood influences the risk of developing diseases like breast and colorectal cancer later in life. It also explores if adult body size impacts this influence (22, 23).

There have been limited publications on the relationship between obesity and DN using Mendelian randomization due to the absence of genome-wide association study (GWAS) data on DN in type 2 diabetes. Only one obesity-related measure, BMI, has been evaluated for a causal relationship with DN (24). In addition, MR studies of abdominal obesity and childhood obesity remain empty.

The intrinsic link between obesity and DN has been better understood because of earlier MR investigations, but there are still a lot of unanswered questions. Furthermore, due to the increasing sample size of genome-wide association studies (GWAS) and the continuous accumulation of data, it is crucial to thoroughly research the relationship between obesity and DN utilizing an MR approach.

In this study, we utilized an extensively expanded collection of IVs derived from the most comprehensive exposure and outcome GWAS conducted to date (25) to (1) assess the overall influence of characteristics related to obesity (general and abdominal obesity, adult and childhood obesity) on DN; (2) Determine the separate causal influence of each characteristic related to obesity while accounting for the confounding influences of glycemic traits, hypertension, and three other noteworthy risk factors; (3) To assess the separate influence of obesity in adulthood and during childhood on DN, while considering their correlation.

## 2 Materials and methods

### 2.1 Data sources and selection of genetic instruments

#### 2.1.1 Genetic variables associated with each obesity-related exposure

The Genetic Investigation of Anthropometric Traits (GIANT) consortium and the UK Biobank (UKBB) worked together to conduct the largest GWAS study of adults with general body obesity (BMI) and abdominal obesity (WHR and WHR_adj_BMI) in 2019. It included an estimated 700,000 people of European ancestry (25). Standard techniques were used to measure anthropometric information such as height, weight, waist, and hip circumference. By dividing the circumferences of the hips and the waist, the Waist-to-Hip Ratio (WHR) was calculated. In contrast, Body Mass Index (BMI) was computed by dividing weight by the square of height. Regressing WHR on BMI and adding BMI as a second independent variable led to the creation of WHR_adj_BMI. The most recent and comprehensive genome-wide association study (GWAS) on childhood BMI was carried out in 2020 by the Early Growth Genetics (EGG) consortium (26). This study included a combined dataset from 41 studies, comprising 39,620 children between the ages of 6 and 10, all of European ancestry.

#### 2.1.2 GWAS summary data for outcome (diabetic nephropathy)

For DN, we defined the outcome as the presence of glomerular damage in patients with diabetes mellitus based on the ICD-10 criterion (code: N08.3*). To obtain the necessary summary statistics, we used data from the FinnGen biobank, which included 3,676 cases and 283,456 controls of European ancestry (27).

#### 2.1.3 Other GWAS(s) of five risk factors

Our MR study now includes five key risk factors: fasting insulin, Homeostasis Model Assessment of Insulin Resistance (HOMA-IR), high blood pressure, circulating CRP levels, and smoking. This inclusion provides a more comprehensive perspective. The summary-level data for glycemic traits, specifically fasting insulin (N=153,525) and HOMA-IR (N=37,037), were sourced from the Meta-analyses of Glucose and Insulin-related Traits Consortium (MAGIC) (28, 29). We obtained the GWAS summary statistics from the IEU Open GWAS project download for high blood pressure, including 124,227 European cases and 337,653 European controls. We used information from a massive GWAS meta-analysis of 88 studies (involving 204,402 individuals) (30) to determine levels of circulating CRP. This GWAS meta-analysis identified 58 significant genetic loci across the entire genome for circulating CRP levels, accounting for up to 7.0% of the variation in circulating CRP levels (30). To analyze smoking, we utilized information released in 2019 by the GSCAN (GWAS & Sequencing Consortium of Alcohol and Nicotine), involving 1,232,091 individuals of European descent, explicitly focusing on smoking initiation (31).

### 2.2 Selection of genetic IVs

We extracted IVs from exposed GWAS that reached genome-wide significance. A total of 85109 SNPs were shown to be independently associated with BMI, 39709 with WHR, 54367 with WHR_adj_BMI, and 1353 with pediatric BMI (all P value< 5×10^-8^).

After conducting the clumping process (R^2^<0.001, window size=10,000kb), the instrumental variables were disentangled from linkage disequilibrium (LD) to ensure their autonomy. Besides, to meet the exclusivity assumption in Mendelian Randomization, we rigorously excluded SNP data with strong associations (P<5×10^-5^) to diabetic nephropathy. This ensures the integrity of our instrumental variable selection. In addition, performing F-statistics to validate significant effects for all IV-SNPs (overall F-statistic value>10) was necessary for the validity of the results.

These SNPs were carefully matched up with the results of the GWAS for DN. See **Supplementary Table 2** for details. We used the following equation $F=(\frac{N-K-1}{K})(\frac{{\text{R}}^{2}}{{1\text{-R}}^{2}})$ to figure out the strength of the device (**Table 1**). The F-statistic of an instrument is considered to be strong enough if it is greater than 10 (32). R2 shows the phenotypic diversity that can be explained by genetic tools. These tools are based on raw GWAS data or can be calculated using the genetic association of an SNP with an exposure (β) and the minor allele frequency (MAF). The following equation $R^{2}={\sum 2\times \hat{\text{β}}}^{2}\times MAF\times (1-MAF)$ was used to figure out R^2^.

**Table 1:** A detailed description of the GWAS data involved in our study.

Phenotype	IV	Sample size	Ethnicity	Consortium	Quotation
BMI	510	806,834	European	Genetic Investigation of ANthropometric Traits (GIANT) and UK BioBank	Pulit, 2019 (25)
WHR	330	697,734	European	Genetic Investigation of ANthropometric Traits (GIANT) and UK BioBank	Pulit, 2019 (25)
WHR_adj_BMI	308	694,649	European	Genetic Investigation of ANthropometric Traits (GIANT) and UK BioBank	Pulit, 2019 (25)
childhood BMI	16	39,620	European	Early Growth Genetics (EGG)	Vogelezang, 2020 (26)
DN	170	3676 cases / 283456 controls	European	FinnGen biobank	Kristiansson K, 2022 (27)
fasting insulin	46	151,013	European	Meta-Analysis of Glucose and Insulin-related traits Consortium (MAGIC)	Chen, 2021 (28)
HOMA-IR	30	37,037	European	Meta-Analysis of Glucose and Insulin-related traits Consortium (MAGIC)	Manning AK, 2012 (29)
high blood pressure	185	461,880	European	MRC-IEU	https://gwas.mrcieu.ac.uk/datasets/ukb-b-14177/
Smoking	83	607,291	European	GWAS & Sequencing Consortium of Alcohol and Nicotine (GSCAN)	https://gwas.mrcieu.ac.uk/datasets/ieu-b-4877/
circulating CRP levels	53	204,402	European	GWAS meta-analysis	Ligthart, 2018 (30)

GWAS, genome-wide association study; IV, instrumental variable; BMI, body mass index; WHR, waist-to-hip ratio; WHR_adj_BMI, waist-to-hip ratio adjusted for body mass index; DN, diabetic nephropathy; HOMA-IR, homeostasis model of insulin resistance;

### 2.3 Statistical analysis

**Figure 1:**
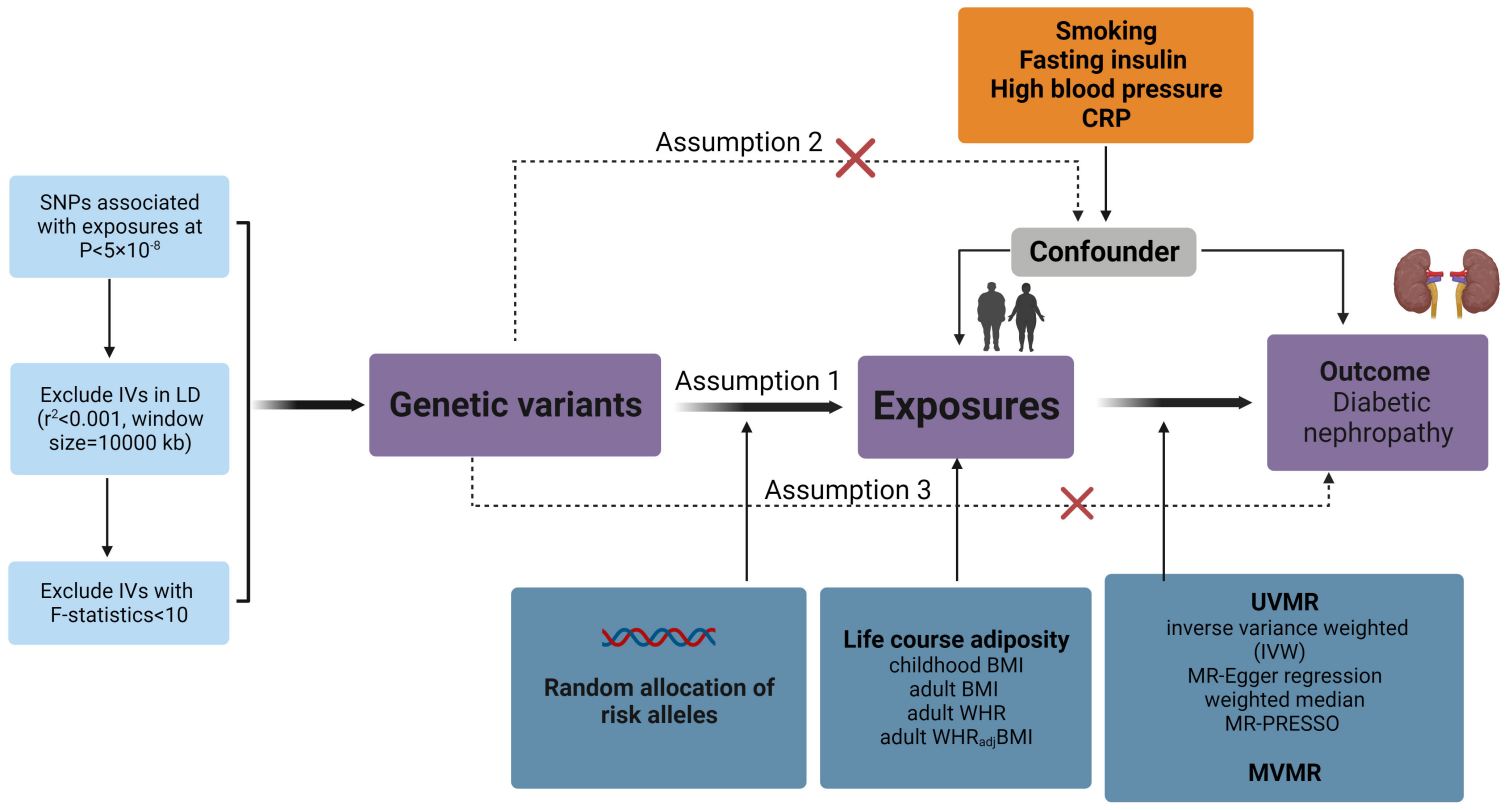
Overview of the MR design assumptions and study methodology. Assumption 1 states that the genetic variants proposed as instrumental variables should be strongly associated with the relevant risk factor; assumption 2 states that the genetic variants used should not be associated with potential confounders; and assumption 3 states that the selected genetic variants should only influence outcome risk through the relevant risk factor and not through other possible pathways. BMI, body mass index; WHR, waist-to-hip ratio; WHR_adj_BMI, waist-to-hip ratio adjusted for body mass index; UVMR, Univariable Mendelian randomization analysis; MVMR, Multivariable Mendelian randomization analysis.

To assess the potential causal relationship between obesity-related exposures (BMI, WHR, WHR_adj_BMI, childhood BMI) and DN, we conducted a comprehensive two-sample (MR) analysis. In **Figure 1**, a schematic diagram is shown to illustrate the method of MR analysis.

#### 2.3.1 Univariable Mendelian randomization analysis

We used UVMR as our primary analysis method to examine the overall impact of each obesity-related characteristic on DN. We first applied the inverse variance weighted (IVW) approach in a random effects model. Regressing the outcome coefficient on the exposure coefficient in this manner yields an estimate of the causal effect without including an intercept term (33). We supplemented the IVW analysis with MR-Egger regression to account for potential bias due to horizontal pleiotropy (34).

MR-Egger regression is similar to IVW but includes an intercept term in the regression model to capture the presence of directional pleiotropy, thereby providing insight into potential bias due to pleiotropic effects. In addition, we used the weighted median approach (35), which is known to be more robust to the inclusion of invalid instruments than IVW and MR-Egger regression.

Furthermore, after locating and eliminating all discovered outlying SNPs, we further employed MR-PRESSO to determine whether horizontal pleiotropy existed and to re-estimate the causal effect (36). By detecting and removing outlying SNPs, we aimed to obtain a more reliable causal effect estimate while accounting for potential horizontal pleiotropy.

We carried out various sensitivity analyses to ensure our results’ robustness. First, we conducted an analysis with IVs that excluded palindromic SNPs with strand ambiguity. The analysis was done to address potential issues related to ambiguous strand orientation that could introduce bias into the analysis. In order to determine if a single SNP had a significant impact on the MR estimate, we also conducted a leave-one-out study. In this analysis, we sequentially left out one SNP at a time and assessed the effect on the results. This approach helped us to identify potential sources of heterogeneity and assess the robustness of the MR estimate. In addition, we also performed Steiger filtering to ensure the directionality of the association between obesity and DN (37).

#### 2.3.2 Multivariable Mendelian randomization analysis

To investigate the independence of the causative effects of childhood and adult obesity on DN, we used a multivariable MR analysis. By adopting this approach, we were able to investigate whether the causal effects of childhood and adult obesity on diabetic nephropathy (DN) are distinct from each other. This provided vital insights into the development of DN in relation to obesity during different life stages.

We included childhood BMI as an additional component to each adult obesity trait (BMI, WHR, and WHR_adj_BMI) to account for the intercorrelation between adult and childhood obesity in order to assess the independent effects of each adult obesity characteristic on DN. To create composite instrumental variables (IVs), we used linkage disequilibrium clustering with an R2 threshold greater than 0.001 (38). Three sets of SNPs were used to create composite IVs: 510 SNPs for BMI and childhood BMI, 327 SNPs for WHR and childhood BMI, and 266 SNPs for WHR_adj_BMI and childhood BMI. These composite IVs allowed us to calculate the direct effect of childhood adiposity on DN while accounting for the effect of adult BMI or vice versa. In our multivariable MR analysis, we implemented IVW, MR-LASSO, and Weighted Median methods to address instrumental variable collinearity and integrated MR-PRESSO to control pleiotropic biases, thereby enhancing our study’s accuracy and interpretability.

In addition, to further explore the independent association between childhood obesity and DN, five risk factors (fasting insulin, HOMA-IR, hypertension, circulating CRP levels, and smoking) considered as important confounders were included. After taking into account the confounding factors, one at a time as well as concurrently to calculate the independent impact of childhood obesity on DN. Odds ratios (ORs) and their 95% confidence intervals (CIs) are used to present the results, which provide a relative risk estimate of DN produced by each increase in standard deviation (SD) of each obesity-related trait examined in this study. P-values were changed into q-values in our MR analysis to take the false discovery rate (FDR) in multiple testing into consideration. FDR-adjusted P-values less than 0.05 were used to define robust statistical significance, and crude P-values less than 0.05 and FDR-adjusted P-values greater than 0.05 were used to define marginal significance. All statistical analyses use R v4.0.0 and the “TwoSampleMR” package (39).

## 3 Results

### 3.1 Univariable Mendelian randomization results

**Figure 2:**
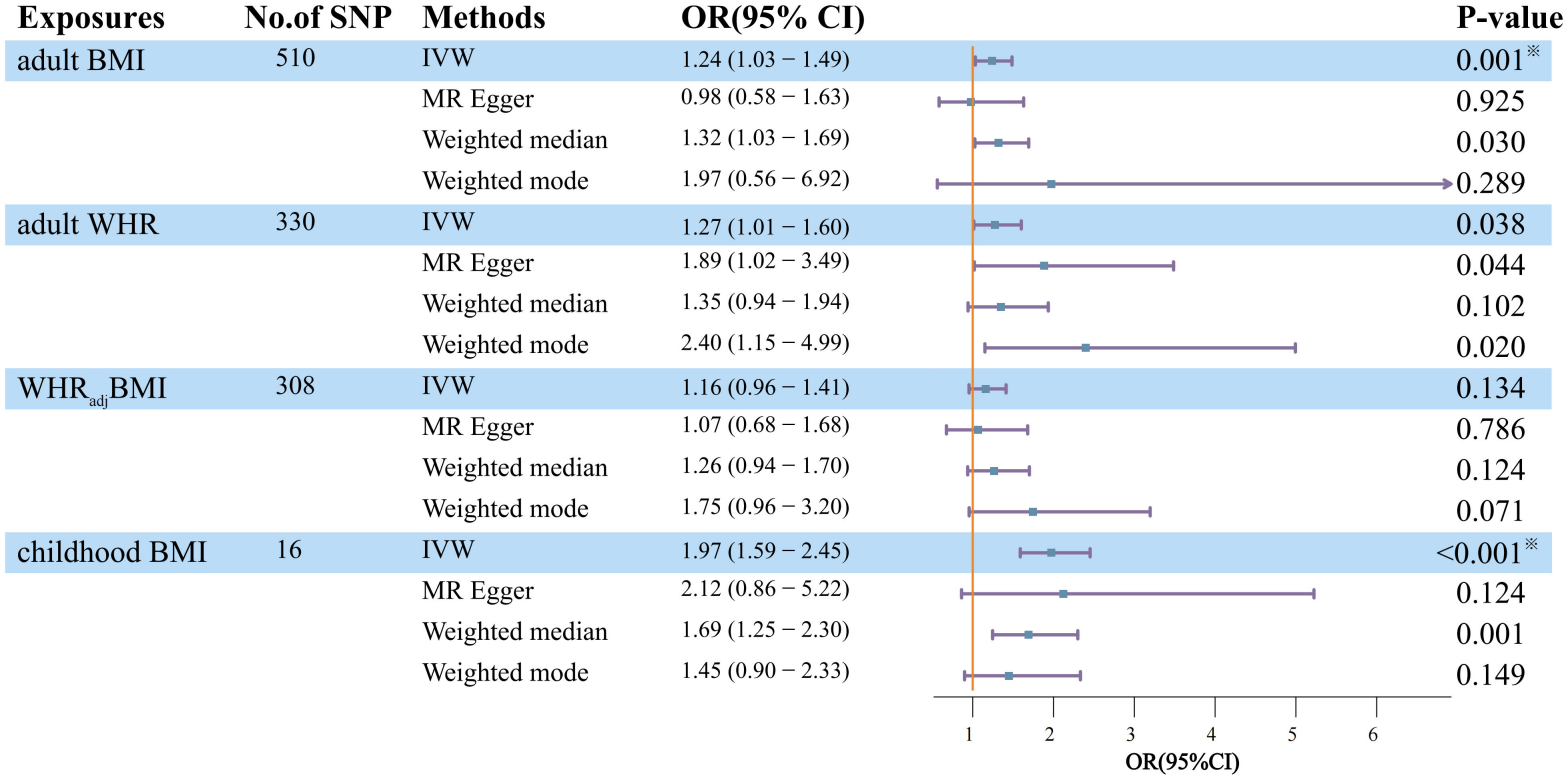
Estimation of the total effect of life course adiposity on the risk of DN using univariable Mendelian randomization. Boxes denote the point estimates of causal effects, and error bars denote 95% confidence intervals. Asterisks (^※^) denote statistical significance survived false discovery rate (FDR) correction (P_FDR_ <0.05). BMI, body mass index; WHR, waist-to-hip ratio; WHR_adj_BMI, waist-to-hip ratio adjusted for body mass index; No. of SNP, number of instrumental variables; OR, odds ratio; 95%CI, 95% confidence interval.

Employing UVMR, genetically predicted BMI showed a statistically significant association with an elevated risk of DN (OR=1.24, 95%CI=1.03-1.49, P=2.06×10^-2^), as shown in **Figure 2**, which also survived FDR correction. Adult WHR showed a marginally significant increase in DN (OR=1.27, 95%CI=1.01-1.60, P=3.80×10^-2^). When the effect of BMI was removed from WHR (WHR_adj_BMI), the results were negative. Regarding childhood BMI, there is convincing evidence that it has a statistically significant hazard effect on DN (OR=1.97, 95%CI= 1.59-2.45, P=8.55×10^-10^). The weighted median method and MR-Egger regression provided additional support for the results of IVW as mentioned above, with estimates that were all in the same general direction.

In contrast to childhood BMI, the Q statistics of the SNP instruments for the other lifelong adiposity measures (BMI, WHR, WHR_adj_BMI) were all less than 0.05, indicating significant heterogeneity among the genetic instruments. To account for this heterogeneity and ensure our results’ robustness, we used a random effects model for these adiposity measures in the analysis.

In addition, MR-Egger regression analysis revealed an intercept centered at zero, indicating no strong evidence for substantial horizontal pleiotropy (MR-Egger intercept>0.05). This suggests that asymmetric pleiotropic effects were less likely to influence the causal estimates generated. However, the significant global test p-value 0.05 of the MR-PRESSO study demonstrated pleiotropy for adult BMI and WHR. Notably, the results remained statistically significant after outlier-corrected filtering, demonstrating the robustness of our findings even after accounting for potential pleiotropic effects.

The funnel plot of the life course adiposity instruments showed a proportional distribution of effect estimates, indicating no significant publication bias. Leave-one-out histogram analysis also failed to identify any specific SNP that had a disproportionate effect on the association between the risk of DN and the overall association. These results demonstrate that the reported associations are legitimate and robust and that the influence of the genetic tools, rather than any particular SNP, is responsible for the overall results. Furthermore, Steiger filtering did not reveal a causal effect of DN on any of the obesity exposures, further assuring directionality (**Supplementary Table 2**).

### 3.2 Multivariable Mendelian randomization results

Although previous evidence has shown that both childhood and adult obesity are associated with an increased risk of DN, it is still uncertain whether their effects are independent of each other. To address this question, we performed a series of MVMR analyses (**Table 2**). Of note, the causal effects of adult BMI and adult abdominal obesity (WHR) on DN were significantly attenuated. They became nonsignificant in MVMR models after accounting for childhood BMI as a covariate. It suggests that the presence of childhood obesity influences the effect of adult general and abdominal obesity on DN. These results imply that childhood obesity may mediate the association between adult obesity and the risk of DN.

**Table 2:** Independent effect of adult obesity and childhood obesity on the risk of DN using MVMR.

	OR (95% CI)	*P-value*
Model 1		
adult BMI	1.15 (0.96-1.37)	0.140
childhood BMI	1.90 (1.60-2.25)	<0.001^※^
Model 2		
adult WHR	1.03 (0.81-1.31)	0.796
childhood BMI	1.80 (1.40-2.31)	<0.001^※^

Model 1: independent effect of adult BMI and childhood BMI on DN; Model 2: independent effect of adult WHR and childhood BMI on DN; Asterisks (※) denote statistical significance survived false discovery rate (FDR) correction (*P*_FDR_<0.05).

BMI, body mass index; WHR, waist-to-hip ratio; DN, diabetic nephropathy; OR, odds ratio; 95%CI, 95% confidence interval.

In contrast, a constant and robust independent effect of childhood BMI on the risk of DN was found even after controlling for each adult adiposity characteristic. The effect of childhood BMI was still substantial and high after adjusting for adult BMI[IVW, OR=1.90, 95% CI =1.60-2.25, P=2.03×10^-13^; LASSO, OR=1.91, 95% CI =1.65-2.21, P=3.80×10^-18^], the independent effect of childhood BMI on DN persisted after adjustment for adult WHR [IVW, OR=1.80, 95% CI=1.40-2.31, P=4.20×10^-6^; LASSO, OR=1.90, 95% CI =1.56-2.32, P=2.76×10^-10^]. These consistent findings across both IVW and MR-Lasso analyses, with p-values significantly less than 0.001, reinforce the independent and substantial impact of childhood BMI on the risk of DN. Furthermore, the implementation of MR-PRESSO, especially after outlier correction, highlighted significant differences, affirming that the exposure effects on DN outcomes are statistically meaningful and robust even when adjusting for pleiotropy. This underscores the reliability and strength of our conclusions.

**Figure 3:**
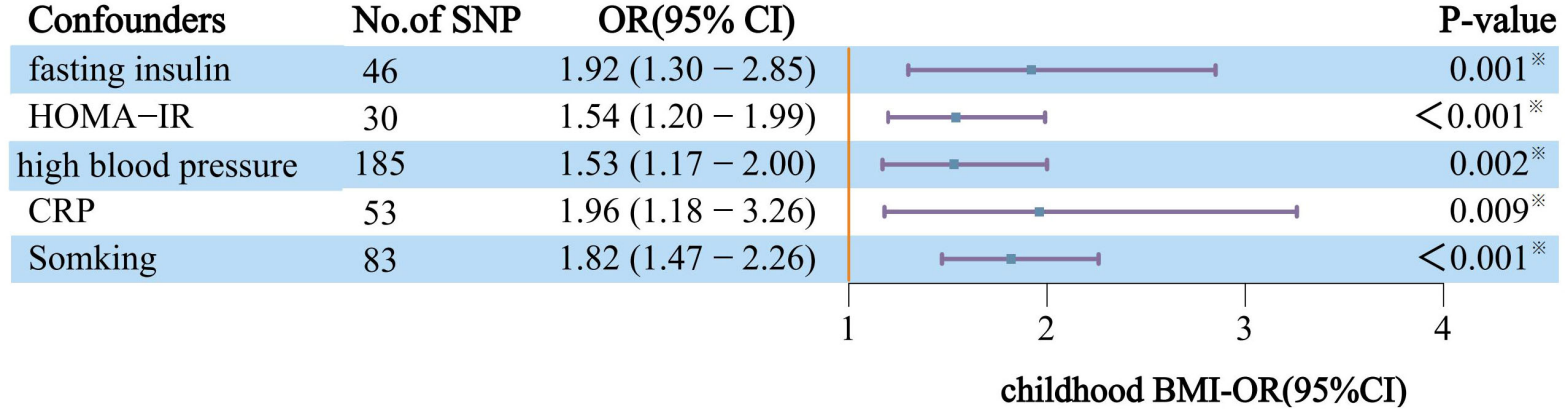
After correcting for each confounder separately and applying MVMR, the effects of genetically predicted childhood obesity on the risk of DN are independent. The y-axis details the genetically predicted confounder(s) for which adjustment was made, and the x-axis details the ORs and 95%Cls per 1-standard deviation (SD) increase in exposure. Asterisks (^※^) denote statistical significance survived false discovery rate (FDR) correction (P_FDR_<0.05). BMI, body mass index; DN, Diabetic nephropathy; CRP, circulating CRP levels; IVW, Inverse-variance weighted approach; NO.SNP, number of instrumental variables; OR, odds ratio; 95%CI, 95% confidence interval.

Lastly, in our MVMR analysis, adjustments for confounders such as fasting insulin, HOMA-IR, high blood pressure, circulating CRP levels, and smoking were meticulously made. These adjustments are crucial for accurately assessing the link between childhood obesity and DN, considering the potential modifying effects of various risk factors. After controlling for covariates, the magnitude and direction of the impact of childhood obesity on DN were all sustained across repeated testing corrections (**Figure 3**).

## 4 Discussion

### 4.1 Main study findings

Using data from the biggest GWAS(s) carried out to date for each variable, our MR investigation evaluated the causative involvement of numerous obesity-related features in the development of DN. We successfully identified the significant deleterious effects of genetically predicted adult BMI, adult WHR, and childhood BMI on DN. Integrating these obesity-related traits, we observed that the impact of adult BMI on DN was mainly mediated by childhood BMI, which also held true for adult WHR. On the contrary, when compared to adult measures, childhood BMI consistently exhibited a distinct and independent risk factor for DN.

### 4.2 Relationship between adult obesity and diabetic nephropathy

Multiple studies have employed an MR method to identify links between genetic susceptibility to overall obesity and DN (24, 40). We additionally investigated the utilization of MVMR to regulate the impact of childhood BMI and discovered a moderation in the impact of adult BMI, indicating that the presumed causal association between adult BMI and DN is primarily due to elevated childhood BMI. When considering these traits collectively, these findings indicate an intricate interaction that underlies various obesity-related characteristics throughout one’s lifetime, emphasizing the significance of simultaneously taking these traits into account.

### 4.3 Relationship between adult abdominal obesity and diabetic nephropathy

A meta-analysis of 14 cross-sectional studies indicated that abdominal obesity parameters were associated with increased odds of DN (6), While Man et al. (8) found that people with abdominal obesity did not have DN in T2DM. Despite the above discrepancies, there is no MR study on the association between abdominal obesity and DN.

Our UVMR revealed a significant detrimental effect of DN using an enlarged collection of IVs that included 330 WHR-associated SNPs. After accounting for childhood BMI, our MVMR also noticed that this protective impact of WHR practically vanished, suggesting that the established putative causality of adult WHR and DN is mainly due to high childhood BMI. There are several possible interpretations of this discrepancy: First, these studies were limited by their observational nature and lack of randomization, prospective design, and blinding. They primarily consisted of observational studies or meta-analyses based on observational data. The discrepancies in the results could be attributed to the inherent limitations of the non-randomized comparative study design itself (41). Second, the association between abdominal obesity and DN identified in observational studies may be due to insidious confounding variables. Despite adjusting for various confounders such as age and diabetes mellitus in numerous studies, there may still be underlying confounders that remain unaccounted for. Hence, further research is warranted to investigate the genetic link between DN and abdominal obesity.

### 4.4 Childhood obesity is a causal risk factor for diabetic nephropathy

Moreover, our research emphasizes the critical contribution of childhood BMI to DN. Based on the findings from our UVMR analysis, it was evident that childhood obesity posed a significant threat to the development of DN. Conversely, our MVMR results offered robust evidence supporting a direct link between childhood obesity and DN, which remained unaffected by adult metrics or other variables.

In recent times, multiple systematic reviews and meta-analyses have consistently demonstrated a significant association between high childhood BMI and an elevated risk of developing adult diabetes, with indications of its potential persistence into adulthood (42–44). Therefore, DN, one of the most common microvascular complications of diabetes, may have potential mechanisms, including long-term childhood obesity status, that directly affect renal function by altering intrarenal hemodynamics, causing oxidative stress, and increasing pro-inflammatory adipokines and cytokines (8), such as insulin resistance, hypertension, and impaired glucose and lipid metabolism, which damage the kidney. Unfortunately, there was not enough data from observational studies to prove the significant association between childhood obesity and DN; therefore, additional experimental studies are necessary to elucidate the precise molecular mechanism behind this discovery.

### 4.5 Strengths and limitations

The current study has several advantages, as we utilized extensive MR to creatively evaluate the distinct impact of various interconnected obesity characteristics on DN. Notably, our findings indicate that childhood obesity has a detrimental effect on DN later in life. However, we also need to acknowledge several limitations. Initially, the presence of pleiotropy caused by undetected confounders may introduce bias to the causal estimates. Nevertheless, we made every effort to minimize such prejudice. We applied the MR-Steiger filtering method separately in UVMR to eliminate SNPs that might suggest reverse causality during the research process. Furthermore, we conducted MVMR analysis by incorporating exposure with confounders individually to prevent pleiotropy. In addition, due to the linear assumption underlying the two-sample MR approach, it was not possible for us to investigate the non-linear relationship between obesity and DN using GWAS summary statistics. Therefore, it may be necessary to conduct future one-sample MR studies utilizing semiparametric methods (45) to address this issue. Given the restricted sample size of DN, it may be necessary to have a more extensive GWAS database for DN to further confirm causality.

In conclusion, we have clarified the increasing correlation between obesity and the risk of DN. Additionally, we discovered that childhood obesity was substantially responsible for the overall impact of adult general obesity and adult abdominal obesity on DN. Finally, we showed that childhood BMI has a largely independent influence on DN, independent of adult measurements. Our findings highlight the importance of childhood obesity in the development of DN and underscore the need to consider the complex interactions that underlie related exposures. Further observational studies or pathophysiological mechanisms of childhood obesity are needed in the future to provide new targets for the prevention and treatment of DN.

## Data availability statement

The original contributions presented in the study are included in the article/**Supplementary Material**. Further inquiries can be directed to the corresponding author.

## Author contributions

HZ: Writing – original draft, Writing – review & editing. QZ: Writing – review & editing. YS: Writing – review & editing. LW: Writing – review & editing. MC: Writing – review & editing. JB: Writing – review & editing. QY: Writing – review & editing.
