# Supplementary material for: Separating the effects of life course adiposity on diabetic nephropathy: a comprehensive multivariable Mendelian randomization study
Source: Front Endocrinol (Lausanne). 2024 Feb 8;15:1285872. doi: 10.3389/fendo.2024.1285872 (PMC10881683; doi:10.3389/fendo.2024.1285872)
Supplement: Supplementary file 1 [file Table_1.docx]

| **Supplementary Table 1.To estimate the overall effect of life course adiposity on the risk of DN using a univariable Mendelian randomization analysis** | | | | | | | | | | | | | | | | | | | | |
| --- | --- | --- | --- | --- | --- | --- | --- | --- | --- | --- | --- | --- | --- | --- | --- | --- | --- | --- | --- | --- |
|  |  | Inverse-variance weighted | | |  | MR-Egger | | | | |  | Weighted median | | |  | MR-PRESSO | | | | |
| Traits | NO.  SNP | OR (95% CI) | *P* | *P-_FDR_* |  | Intercept (SE) | *P^＊^* | OR (95% CI) | *P* | *P-_FDR_* |  | OR (95% CI) | *P* | *P-_FDR_* |  | Global Test  *P-value* | NO.  SNP*^#^* | OR (95% CI) | *P* | *P-_FDR_* |
| adult BMI | 510 | 1.24(1.03−1.49) | 2.06×  10^−2^ | 4.12×10^−2^ |  | 0.004  (0.00) | 0.33 | 0.98(0.58−1.63) | 0.93 | 0.93 |  | 1.32(1.03−1.69) | 3.01×10^−2^ | 6.20×10^−2^ |  | <0.05 | 508 | 1.27(  1.06−1.52) | 9.88×10^−3^ | 9.88×10^−3^ |
| adult WHR | 330 | 1.27(1.01−1.60) | 3.80×  10^−2^ | 0.06 |  | -0.007  (0.01) | 0.18 | 1.89(1.02−3.49) | 4.39×10^−2^ | 0.18 |  | 1.35(0.94−1.94) | 0.10 | 0.12 |  | <0.05 | 329 | 1.27(  1.01−1.60) | 2.02×10^−2^ | 2.02×10^−2^ |
| adult WHR_adj_BMI | 308 | 1.17( 0.96−1.41) | 0.13 | 0.13 |  | 0.00  (0.00) | 0.68 | 1.07(0.68−1.68) | 0.79 | 0.93 |  | 1.26(0.94−1.70) | 0.12 | 0.12 |  | <0.05 | 307 | 1.16(  0.96−1.41) | 0.09 | 0.09 |
| childhood BMI | 16 | 1.97(1.59−2.45) | 8.55×  10^−10^ | 3.42×10^−9^ |  | 0.01  (0.01) | 0.49 | 2.12(0.86−5.22) | 0.12 | 0.24 |  | 1.69(1.25−2.30) | 7.61×  10^−4^ | 3.04×10^−3^ |  | 0.44 | NA | NA | NA | NA |
| Asterisk (^＊^) denotes *P* -value for intercept (pleiotropy) in MR-Egger analysis. Asterisk (*^#^*) denotes Number of single nucleotide polymorphisms (SNPs) of instrumental variables in MR-PRESSO analysis(Outlier-corrected).  Abbreviations: NO.SNP, the number of SNPs of instrumental variables; BMI, body mass index; WHR, waist-to-hip ratio; WHR_adj_BMI, waist-to-hip ratio adjusted for body mass index; DN, diabetic nephropathy; OR, odds ratio; 95% CI, 95% confidence interval. | | | | | | | | | | | | | | | | | | | | |

| **Supplementary Table 2. Characteristics of index SNPs associated with life course adiposity and their effect sizes with outcomes^※^** | | | | | | | | | | | | | |
| --- | --- | --- | --- | --- | --- | --- | --- | --- | --- | --- | --- | --- | --- |
| **SNP** | **Chr** | **Position** | **EAF** | **A1** | **A2** | **Exposure** | | | DN | | | Steiger filtering | |
|  |  |  |  |  |  | **beta** | **se** | ***P*-value** | **beta** | **se** | ***P*-value** | **direction** | ***P*-value** |
| **adult BMI** | | | | | | | | | | | | | |
| rs10002111 | 4 | 67815504 | 0.22 | G | A | 0.013 | 0.002 | 1.26E-09 | 0.026 | 0.029 | 3.64E-01 | TRUE | 2.32E-02 |
| rs10033843 | 4 | 77028783 | 0.22 | A | G | 0.014 | 0.002 | 1.07E-11 | 0.022 | 0.028 | 4.38E-01 | TRUE | 5.98E-03 |
| rs10050620 | 5 | 63927239 | 0.33 | T | C | -0.013 | 0.002 | 1.20E-10 | -0.045 | 0.025 | 7.06E-02 | TRUE | 7.55E-02 |
| rs1006317 | 1 | 209552636 | 0.13 | T | G | 0.016 | 0.003 | 3.75E-10 | 0.063 | 0.037 | 8.92E-02 | TRUE | 9.04E-02 |
| rs10099330 | 8 | 143383694 | 0.54 | G | A | -0.012 | 0.002 | 3.22E-12 | 0.012 | 0.024 | 6.30E-01 | TRUE | 1.51E-03 |
| rs10101364 | 8 | 20634888 | 0.68 | T | C | 0.012 | 0.002 | 5.61E-11 | -0.005 | 0.024 | 8.25E-01 | TRUE | 1.26E-03 |
| rs10110189 | 8 | 15393380 | 0.11 | C | T | -0.016 | 0.003 | 2.20E-08 | 0.076 | 0.054 | 1.58E-01 | TRUE | 1.01E-01 |
| rs10132280 | 14 | 25928179 | 0.31 | A | C | -0.021 | 0.002 | 2.28E-33 | -0.025 | 0.025 | 3.22E-01 | TRUE | 1.61E-07 |
| rs10145749 | 14 | 102782109 | 0.15 | T | C | 0.016 | 0.003 | 8.33E-10 | 0.012 | 0.032 | 7.00E-01 | TRUE | 4.49E-03 |
| rs10168563 | 2 | 35552173 | 0.70 | A | G | 0.013 | 0.002 | 9.33E-12 | 0.043 | 0.025 | 7.69E-02 | TRUE | 3.87E-02 |
| rs10169594 | 2 | 41637688 | 0.65 | C | T | -0.012 | 0.002 | 1.34E-11 | -0.017 | 0.025 | 5.04E-01 | TRUE | 4.49E-03 |
| rs10182181 | 2 | 25150296 | 0.51 | G | A | -0.033 | 0.002 | 2.45E-91 | 0.059 | 0.024 | 1.48E-02 | TRUE | 5.50E-17 |
| rs10197031 | 2 | 105454590 | 0.73 | C | T | -0.016 | 0.002 | 5.05E-18 | 0.024 | 0.025 | 3.45E-01 | TRUE | 4.16E-04 |
| rs10261050 | 7 | 114337652 | 0.48 | T | C | 0.011 | 0.002 | 4.53E-11 | 0.052 | 0.024 | 3.09E-02 | TRUE | 1.21E-01 |
| rs1048637 | 3 | 13358171 | 0.55 | G | T | -0.009 | 0.002 | 2.94E-08 | 0.033 | 0.024 | 1.63E-01 | TRUE | 1.02E-01 |
| rs10499694 | 7 | 50614173 | 0.49 | G | A | 0.013 | 0.002 | 1.29E-15 | 0.035 | 0.024 | 1.48E-01 | TRUE | 3.51E-03 |
| rs10506971 | 12 | 89757937 | 0.55 | A | G | -0.014 | 0.002 | 4.69E-17 | 0.040 | 0.024 | 1.00E-01 | TRUE | 4.13E-03 |
| rs10510419 | 3 | 12426936 | 0.15 | T | G | -0.017 | 0.002 | 2.23E-13 | 0.010 | 0.034 | 7.73E-01 | TRUE | 4.75E-04 |
| rs10518694 | 15 | 53072673 | 0.14 | A | C | 0.014 | 0.002 | 3.43E-09 | 0.072 | 0.038 | 5.87E-02 | TRUE | 1.47E-01 |
| rs10733051 | 1 | 167280354 | 0.51 | G | A | 0.009 | 0.002 | 6.96E-09 | 0.004 | 0.024 | 8.63E-01 | TRUE | 4.67E-03 |
| rs10733682 | 9 | 129460914 | 0.46 | G | A | 0.015 | 0.002 | 1.76E-19 | -0.009 | 0.024 | 7.12E-01 | TRUE | 9.79E-06 |
| rs10741329 | 11 | 89997796 | 0.69 | A | G | 0.012 | 0.002 | 3.91E-10 | 0.036 | 0.027 | 1.77E-01 | TRUE | 3.44E-02 |
| rs10742752 | 11 | 45438374 | 0.38 | C | T | -0.012 | 0.002 | 1.20E-13 | 0.003 | 0.024 | 9.02E-01 | TRUE | 3.18E-04 |
| rs1075901 | 17 | 15943910 | 0.45 | C | T | -0.012 | 0.002 | 4.43E-13 | -0.020 | 0.024 | 3.97E-01 | TRUE | 2.28E-03 |
| rs10761785 | 10 | 65318766 | 0.51 | T | G | -0.013 | 0.002 | 3.47E-16 | -0.017 | 0.024 | 4.86E-01 | TRUE | 2.53E-04 |
| rs10772983 | 12 | 17141582 | 0.54 | T | C | -0.010 | 0.002 | 6.21E-10 | 0.001 | 0.024 | 9.68E-01 | TRUE | 1.54E-03 |
| rs10779751 | 1 | 11284336 | 0.28 | G | A | 0.013 | 0.002 | 2.66E-13 | 0.029 | 0.027 | 2.88E-01 | TRUE | 4.86E-03 |
| rs10811868 | 9 | 23199959 | 0.32 | A | G | -0.010 | 0.002 | 3.18E-08 | -0.033 | 0.026 | 2.05E-01 | TRUE | 7.90E-02 |
| rs10829164 | 10 | 27318370 | 0.15 | T | C | 0.015 | 0.002 | 2.37E-10 | 0.014 | 0.028 | 6.20E-01 | TRUE | 5.15E-03 |
| rs10842240 | 12 | 24060075 | 0.13 | C | G | 0.020 | 0.003 | 1.21E-14 | 0.021 | 0.033 | 5.27E-01 | TRUE | 5.54E-04 |
| rs10858334 | 9 | 137989785 | 0.85 | G | C | -0.015 | 0.003 | 4.91E-09 | -0.037 | 0.044 | 4.01E-01 | TRUE | 2.08E-02 |
| rs10864728 | 1 | 230304914 | 0.40 | G | A | 0.011 | 0.002 | 1.20E-08 | -0.048 | 0.024 | 4.65E-02 | TRUE | 2.09E-01 |
| rs10909880 | 1 | 2727804 | 0.45 | T | C | -0.014 | 0.002 | 2.21E-16 | 0.048 | 0.024 | 4.72E-02 | TRUE | 8.84E-03 |
| rs10920336 | 1 | 202115945 | 0.53 | A | G | -0.010 | 0.002 | 4.20E-09 | -0.035 | 0.024 | 1.35E-01 | TRUE | 7.82E-02 |
| rs10920678 | 1 | 190239907 | 0.43 | G | A | 0.015 | 0.002 | 7.15E-20 | -0.013 | 0.024 | 5.76E-01 | TRUE | 1.78E-05 |
| rs10929925 | 2 | 6155557 | 0.41 | A | C | -0.014 | 0.002 | 3.05E-18 | -0.044 | 0.024 | 6.51E-02 | TRUE | 3.05E-03 |
| rs10938397 | 4 | 45182527 | 0.57 | G | A | -0.032 | 0.002 | 2.42E-86 | 0.046 | 0.024 | 5.48E-02 | TRUE | 4.71E-18 |
| rs10942267 | 5 | 80841914 | 0.69 | G | A | 0.015 | 0.002 | 6.53E-16 | 0.035 | 0.029 | 2.15E-01 | TRUE | 1.64E-03 |
| rs10961649 | 9 | 14670949 | 0.32 | T | C | 0.010 | 0.002 | 1.51E-08 | -0.007 | 0.026 | 7.79E-01 | TRUE | 6.54E-03 |
| rs10962550 | 9 | 16720329 | 0.18 | C | G | 0.019 | 0.002 | 5.68E-17 | 0.079 | 0.031 | 1.15E-02 | TRUE | 3.06E-02 |
| rs10968114 | 9 | 27800007 | 0.53 | C | A | 0.011 | 0.002 | 3.30E-11 | -0.004 | 0.024 | 8.57E-01 | TRUE | 1.15E-03 |
| rs10975933 | 9 | 6954557 | 0.66 | G | C | 0.011 | 0.002 | 2.07E-10 | 0.004 | 0.024 | 8.86E-01 | TRUE | 1.80E-03 |
| rs10992867 | 9 | 96461013 | 0.27 | A | G | 0.016 | 0.002 | 3.20E-17 | -0.022 | 0.026 | 3.91E-01 | TRUE | 2.81E-04 |
| rs11001259 | 10 | 76807650 | 0.18 | A | T | -0.014 | 0.002 | 1.22E-08 | -0.020 | 0.031 | 5.14E-01 | TRUE | 1.71E-02 |
| rs11030618 | 11 | 29243293 | 0.58 | T | C | 0.011 | 0.002 | 1.67E-10 | -0.032 | 0.024 | 1.80E-01 | TRUE | 3.04E-02 |
| rs11044430 | 12 | 19287416 | 0.84 | A | T | 0.016 | 0.002 | 3.77E-12 | -0.023 | 0.035 | 5.12E-01 | TRUE | 2.50E-03 |
| rs11046972 | 12 | 23705969 | 0.07 | T | C | 0.018 | 0.003 | 3.72E-08 | 0.062 | 0.037 | 9.67E-02 | TRUE | 1.64E-01 |
| rs11060853 | 12 | 123424071 | 0.59 | G | A | -0.011 | 0.002 | 3.10E-08 | 0.046 | 0.024 | 6.04E-02 | TRUE | 2.03E-01 |
| rs11066188 | 12 | 112610714 | 0.38 | A | G | -0.011 | 0.002 | 3.05E-12 | 0.039 | 0.024 | 1.10E-01 | TRUE | 2.27E-02 |
| rs11078883 | 17 | 2138828 | 0.65 | G | C | -0.013 | 0.002 | 1.30E-12 | -0.007 | 0.026 | 7.95E-01 | TRUE | 7.72E-04 |
| rs11105839 | 12 | 91237920 | 0.37 | A | T | -0.011 | 0.002 | 1.25E-11 | 0.043 | 0.025 | 8.40E-02 | TRUE | 5.47E-02 |
| rs11115176 | 12 | 82465797 | 0.78 | C | T | 0.013 | 0.002 | 6.79E-12 | 0.016 | 0.028 | 5.72E-01 | TRUE | 2.31E-03 |
| rs11121210 | 1 | 8708529 | 0.34 | C | T | -0.011 | 0.002 | 2.37E-10 | -0.042 | 0.026 | 1.04E-01 | TRUE | 5.13E-02 |
| rs11128021 | 3 | 88139016 | 0.16 | A | G | -0.018 | 0.002 | 1.63E-14 | -0.069 | 0.031 | 2.59E-02 | TRUE | 5.11E-02 |
| rs11150911 | 18 | 73498528 | 0.28 | C | A | 0.012 | 0.002 | 3.72E-11 | -0.033 | 0.026 | 2.01E-01 | TRUE | 2.37E-02 |
| rs11165643 | 1 | 96924097 | 0.58 | T | C | 0.019 | 0.002 | 4.49E-30 | -0.023 | 0.024 | 3.37E-01 | TRUE | 3.43E-07 |
| rs11170468 | 12 | 39430048 | 0.78 | C | A | 0.013 | 0.002 | 1.12E-11 | -0.045 | 0.030 | 1.40E-01 | TRUE | 2.53E-02 |
| rs11218510 | 11 | 121922587 | 0.39 | A | G | -0.014 | 0.002 | 6.79E-13 | 0.001 | 0.025 | 9.57E-01 | TRUE | 4.00E-04 |
| rs11246136 | 11 | 371265 | 0.10 | A | C | -0.017 | 0.003 | 2.03E-08 | -0.045 | 0.043 | 2.96E-01 | TRUE | 5.08E-02 |
| rs112646560 | 1 | 39560250 | 0.21 | T | C | 0.018 | 0.002 | 1.29E-14 | 0.029 | 0.030 | 3.40E-01 | TRUE | 1.43E-03 |
| rs1126930 | 12 | 49399132 | 0.03 | C | G | 0.034 | 0.005 | 1.00E-10 | -0.006 | 0.069 | 9.34E-01 | TRUE | 1.26E-03 |
| rs11525873 | 7 | 138817193 | 0.90 | C | T | 0.023 | 0.003 | 2.98E-13 | -0.022 | 0.033 | 5.10E-01 | TRUE | 1.64E-03 |
| rs11538 | 22 | 18220831 | 0.83 | G | A | -0.014 | 0.002 | 1.09E-09 | -0.014 | 0.030 | 6.42E-01 | TRUE | 7.48E-03 |
| rs11577094 | 1 | 38026600 | 0.08 | T | C | 0.019 | 0.003 | 3.28E-10 | -0.025 | 0.041 | 5.43E-01 | TRUE | 7.95E-03 |
| rs11594179 | 10 | 104392580 | 0.23 | T | C | -0.011 | 0.002 | 2.16E-08 | -0.032 | 0.031 | 3.15E-01 | TRUE | 3.79E-02 |
| rs1159692 | 5 | 63977815 | 0.48 | C | A | 0.014 | 0.002 | 5.54E-15 | 0.010 | 0.024 | 6.68E-01 | TRUE | 2.15E-04 |
| rs11611246 | 12 | 939480 | 0.20 | T | G | 0.022 | 0.002 | 2.04E-28 | 0.071 | 0.029 | 1.48E-02 | TRUE | 2.95E-04 |
| rs11614340 | 12 | 133426483 | 0.71 | C | T | -0.012 | 0.002 | 1.87E-10 | 0.020 | 0.027 | 4.54E-01 | TRUE | 7.22E-03 |
| rs11615578 | 12 | 121714935 | 0.26 | T | C | 0.012 | 0.002 | 3.04E-09 | -0.003 | 0.028 | 9.03E-01 | TRUE | 3.82E-03 |
| rs11633626 | 15 | 95271378 | 0.63 | A | C | -0.016 | 0.002 | 7.27E-19 | 0.007 | 0.024 | 7.67E-01 | TRUE | 2.50E-05 |
| rs11636611 | 15 | 36391965 | 0.50 | T | C | 0.010 | 0.002 | 8.85E-10 | 0.004 | 0.024 | 8.51E-01 | TRUE | 2.96E-03 |
| rs116374395 | 5 | 50723410 | 0.03 | A | G | 0.032 | 0.005 | 7.05E-10 | -0.094 | 0.068 | 1.65E-01 | TRUE | 4.90E-02 |
| rs11649864 | 17 | 56093061 | 0.09 | A | G | 0.019 | 0.003 | 2.52E-10 | -0.050 | 0.052 | 3.40E-01 | TRUE | 1.39E-02 |
| rs11655587 | 17 | 47140794 | 0.36 | T | C | -0.021 | 0.002 | 6.87E-26 | -0.043 | 0.025 | 8.38E-02 | TRUE | 9.85E-05 |
| rs11672660 | 19 | 46180184 | 0.19 | T | C | -0.034 | 0.002 | 6.83E-60 | 0.015 | 0.027 | 5.78E-01 | TRUE | 8.00E-15 |
| rs11692326 | 2 | 208263279 | 0.23 | T | C | 0.015 | 0.002 | 1.69E-14 | 0.031 | 0.029 | 2.75E-01 | TRUE | 2.47E-03 |
| rs11695013 | 2 | 157057487 | 0.63 | T | C | -0.010 | 0.002 | 2.43E-09 | 0.015 | 0.025 | 5.42E-01 | TRUE | 9.06E-03 |
| rs11713193 | 3 | 49924424 | 0.52 | A | G | 0.025 | 0.002 | 3.02E-48 | -0.007 | 0.024 | 7.79E-01 | TRUE | 7.37E-13 |
| rs11739877 | 5 | 105876806 | 0.62 | T | C | 0.012 | 0.002 | 4.01E-11 | 0.012 | 0.024 | 6.29E-01 | TRUE | 3.89E-03 |
| rs11757278 | 6 | 13180454 | 0.70 | C | T | 0.013 | 0.002 | 6.92E-13 | 0.044 | 0.024 | 7.03E-02 | TRUE | 4.22E-02 |
| rs11772246 | 7 | 71603692 | 0.82 | C | T | 0.015 | 0.002 | 3.69E-11 | -0.036 | 0.029 | 2.22E-01 | TRUE | 1.99E-02 |
| rs11773362 | 7 | 147668180 | 0.34 | T | C | -0.011 | 0.002 | 6.39E-09 | -0.011 | 0.024 | 6.65E-01 | TRUE | 8.89E-03 |
| rs11782074 | 8 | 142617096 | 0.37 | T | G | 0.012 | 0.002 | 4.24E-12 | 0.037 | 0.025 | 1.31E-01 | TRUE | 2.56E-02 |
| rs118081010 | 11 | 46174948 | 0.02 | T | C | 0.052 | 0.008 | 5.28E-12 | 0.450 | 0.189 | 1.73E-02 | TRUE | 1.35E-01 |
| rs11882409 | 19 | 34019685 | 0.30 | A | C | 0.012 | 0.002 | 3.31E-10 | 0.005 | 0.031 | 8.67E-01 | TRUE | 1.82E-03 |
| rs11902450 | 2 | 12845368 | 0.11 | T | C | 0.017 | 0.003 | 1.06E-09 | -0.047 | 0.058 | 4.14E-01 | TRUE | 1.36E-02 |
| rs11915371 | 3 | 70539559 | 0.80 | C | A | -0.015 | 0.002 | 2.29E-13 | 0.089 | 0.029 | 2.50E-03 | TRUE | 2.46E-01 |
| rs11919665 | 3 | 48085349 | 0.32 | T | A | 0.012 | 0.002 | 7.30E-09 | -0.038 | 0.027 | 1.68E-01 | TRUE | 7.00E-02 |
| rs11921432 | 3 | 35117776 | 0.89 | C | T | -0.019 | 0.003 | 5.38E-12 | -0.006 | 0.043 | 8.90E-01 | TRUE | 5.25E-04 |
| rs12072739 | 1 | 98315893 | 0.78 | G | A | -0.017 | 0.002 | 1.50E-13 | 0.022 | 0.026 | 3.89E-01 | TRUE | 2.49E-03 |
| rs12098284 | 10 | 76047464 | 0.12 | T | C | 0.018 | 0.003 | 9.87E-13 | -0.003 | 0.046 | 9.56E-01 | TRUE | 3.46E-04 |
| rs12140153 | 1 | 62579891 | 0.09 | T | G | -0.035 | 0.003 | 1.44E-25 | -0.037 | 0.044 | 4.03E-01 | TRUE | 4.21E-06 |
| rs12150665 | 17 | 34914787 | 0.59 | C | T | 0.017 | 0.002 | 1.74E-24 | -0.077 | 0.025 | 1.93E-03 | TRUE | 6.62E-03 |
| rs12259464 | 10 | 53680099 | 0.48 | A | G | 0.011 | 0.002 | 1.48E-10 | -0.018 | 0.024 | 4.59E-01 | TRUE | 8.09E-03 |
| rs12282785 | 11 | 76476030 | 0.22 | A | C | -0.016 | 0.002 | 1.43E-11 | -0.002 | 0.029 | 9.46E-01 | TRUE | 5.84E-04 |
| rs12286929 | 11 | 115022404 | 0.50 | G | A | -0.018 | 0.002 | 1.93E-27 | 0.030 | 0.024 | 2.12E-01 | TRUE | 4.32E-06 |
| rs12334877 | 8 | 67194171 | 0.19 | A | G | -0.014 | 0.002 | 2.17E-11 | -0.051 | 0.028 | 7.26E-02 | TRUE | 4.87E-02 |
| rs12364470 | 11 | 134601012 | 0.85 | G | T | -0.019 | 0.002 | 2.18E-17 | 0.022 | 0.028 | 4.35E-01 | TRUE | 2.30E-04 |
| rs12369179 | 12 | 122963550 | 0.09 | T | C | -0.034 | 0.003 | 2.32E-28 | 0.003 | 0.043 | 9.42E-01 | TRUE | 2.75E-08 |
| rs12386885 | 8 | 87766769 | 0.16 | T | C | 0.015 | 0.003 | 9.90E-09 | 0.011 | 0.032 | 7.23E-01 | TRUE | 7.57E-03 |
| rs12421848 | 11 | 891338 | 0.40 | A | G | -0.014 | 0.002 | 3.99E-13 | -0.037 | 0.025 | 1.40E-01 | TRUE | 1.12E-02 |
| rs12429545 | 13 | 54102206 | 0.12 | A | G | 0.031 | 0.002 | 1.42E-37 | 0.029 | 0.034 | 3.99E-01 | TRUE | 2.57E-09 |
| rs12439632 | 15 | 59103963 | 0.17 | C | G | 0.015 | 0.002 | 3.68E-10 | 0.006 | 0.028 | 8.38E-01 | TRUE | 2.25E-03 |
| rs12448257 | 16 | 3599655 | 0.22 | A | G | 0.016 | 0.002 | 9.04E-16 | 0.073 | 0.032 | 2.44E-02 | TRUE | 2.85E-02 |
| rs12462975 | 19 | 30272202 | 0.32 | A | G | 0.019 | 0.002 | 1.47E-25 | -0.055 | 0.026 | 3.26E-02 | TRUE | 2.54E-04 |
| rs12591120 | 15 | 99236869 | 0.74 | C | T | 0.012 | 0.002 | 3.84E-08 | 0.003 | 0.028 | 9.22E-01 | TRUE | 6.73E-03 |
| rs12602912 | 17 | 65870073 | 0.21 | T | C | 0.017 | 0.002 | 2.90E-16 | 0.057 | 0.028 | 4.13E-02 | TRUE | 1.24E-02 |
| rs12611148 | 19 | 19865077 | 0.14 | A | C | -0.014 | 0.002 | 1.42E-08 | 0.009 | 0.038 | 8.17E-01 | TRUE | 6.41E-03 |
| rs12628051 | 22 | 40654276 | 0.64 | C | T | 0.016 | 0.002 | 2.90E-19 | -0.054 | 0.024 | 2.75E-02 | TRUE | 7.16E-03 |
| rs12628891 | 22 | 38317137 | 0.32 | T | C | -0.012 | 0.002 | 5.85E-10 | -0.078 | 0.027 | 3.78E-03 | TRUE | 5.39E-01 |
| rs12636480 | 3 | 82719412 | 0.35 | T | G | 0.013 | 0.002 | 6.96E-13 | -0.009 | 0.026 | 7.26E-01 | TRUE | 8.31E-04 |
| rs12652212 | 5 | 88808594 | 0.56 | G | A | -0.013 | 0.002 | 1.63E-15 | -0.004 | 0.025 | 8.78E-01 | TRUE | 4.85E-05 |
| rs1268065 | 6 | 126042783 | 0.50 | A | G | -0.010 | 0.002 | 7.12E-10 | -0.025 | 0.024 | 2.87E-01 | TRUE | 2.21E-02 |
| rs12680842 | 8 | 95582606 | 0.68 | G | A | 0.014 | 0.002 | 3.41E-16 | -0.022 | 0.024 | 3.53E-01 | TRUE | 4.98E-04 |
| rs12681792 | 8 | 62054463 | 0.20 | A | C | 0.015 | 0.002 | 2.88E-12 | 0.018 | 0.025 | 4.67E-01 | TRUE | 2.41E-03 |
| rs12692596 | 2 | 161265910 | 0.36 | T | C | 0.012 | 0.002 | 1.03E-12 | -0.016 | 0.025 | 5.25E-01 | TRUE | 2.13E-03 |
| rs12714199 | 2 | 86812549 | 0.61 | T | C | -0.014 | 0.002 | 3.22E-16 | 0.017 | 0.024 | 4.72E-01 | TRUE | 2.82E-04 |
| rs12765914 | 10 | 34013507 | 0.08 | T | C | 0.023 | 0.003 | 1.96E-13 | 0.039 | 0.038 | 2.96E-01 | TRUE | 4.54E-03 |
| rs12888545 | 14 | 88308044 | 0.75 | G | A | -0.013 | 0.002 | 1.76E-11 | 0.024 | 0.028 | 3.87E-01 | TRUE | 7.73E-03 |
| rs12912198 | 15 | 47103953 | 0.27 | T | C | -0.010 | 0.002 | 3.44E-08 | -0.061 | 0.027 | 2.35E-02 | TRUE | 3.68E-01 |
| rs12914623 | 15 | 80993570 | 0.27 | C | G | -0.016 | 0.002 | 2.00E-16 | -0.018 | 0.028 | 5.09E-01 | TRUE | 1.99E-04 |
| rs12922346 | 16 | 82438337 | 0.26 | C | G | 0.013 | 0.002 | 1.48E-11 | -0.015 | 0.030 | 6.08E-01 | TRUE | 3.02E-03 |
| rs12926250 | 16 | 72213316 | 0.11 | T | G | 0.018 | 0.003 | 1.78E-10 | 0.087 | 0.050 | 8.14E-02 | TRUE | 7.24E-02 |
| rs1293037 | 6 | 70248345 | 0.75 | T | C | 0.013 | 0.002 | 5.73E-09 | 0.012 | 0.031 | 6.86E-01 | TRUE | 7.88E-03 |
| rs12939549 | 17 | 78611724 | 0.56 | G | A | 0.018 | 0.002 | 3.68E-28 | -0.013 | 0.025 | 5.99E-01 | TRUE | 1.08E-07 |
| rs1296328 | 4 | 137083193 | 0.45 | C | A | 0.017 | 0.002 | 3.49E-22 | -0.007 | 0.024 | 7.69E-01 | TRUE | 2.03E-06 |
| rs12981256 | 19 | 1865901 | 0.53 | A | G | 0.015 | 0.002 | 1.43E-18 | -0.007 | 0.024 | 7.82E-01 | TRUE | 1.61E-05 |
| rs13021737 | 2 | 632348 | 0.16 | G | A | -0.058 | 0.002 | 2.89E-161 | 0.036 | 0.032 | 2.55E-01 | TRUE | 2.51E-39 |
| rs13033310 | 2 | 133523605 | 0.25 | A | G | 0.015 | 0.002 | 3.40E-11 | 0.009 | 0.028 | 7.34E-01 | TRUE | 1.88E-03 |
| rs1304549 | 20 | 54378256 | 0.23 | A | G | -0.012 | 0.002 | 1.35E-08 | -0.009 | 0.030 | 7.63E-01 | TRUE | 8.81E-03 |
| rs13107325 | 4 | 103188709 | 0.08 | T | C | 0.047 | 0.003 | 3.81E-47 | 0.075 | 0.102 | 4.62E-01 | TRUE | 6.88E-12 |
| rs13110266 | 4 | 162129844 | 0.40 | A | G | -0.012 | 0.002 | 3.96E-14 | 0.000 | 0.024 | 9.94E-01 | TRUE | 7.36E-05 |
| rs13174863 | 5 | 139080745 | 0.85 | G | A | -0.020 | 0.002 | 1.94E-17 | -0.028 | 0.033 | 4.02E-01 | TRUE | 2.44E-04 |
| rs13186194 | 5 | 60795485 | 0.62 | C | T | 0.010 | 0.002 | 2.20E-09 | -0.009 | 0.025 | 7.15E-01 | TRUE | 6.93E-03 |
| rs13191362 | 6 | 163033350 | 0.86 | G | A | 0.024 | 0.003 | 4.08E-21 | 0.019 | 0.049 | 6.98E-01 | TRUE | 7.39E-06 |
| rs13245051 | 7 | 113362799 | 0.46 | G | A | 0.015 | 0.002 | 1.12E-18 | 0.026 | 0.024 | 2.78E-01 | TRUE | 3.32E-04 |
| rs13263601 | 8 | 14095900 | 0.66 | C | A | -0.015 | 0.002 | 4.87E-16 | 0.013 | 0.027 | 6.28E-01 | TRUE | 2.06E-04 |
| rs1327259 | 6 | 51177811 | 0.61 | G | A | 0.016 | 0.002 | 1.47E-19 | -0.045 | 0.024 | 5.82E-02 | TRUE | 1.91E-03 |
| rs13296413 | 9 | 37258105 | 0.38 | T | C | -0.015 | 0.002 | 2.49E-17 | -0.037 | 0.026 | 1.49E-01 | TRUE | 1.28E-03 |
| rs13298487 | 9 | 126112104 | 0.61 | C | T | 0.012 | 0.002 | 1.43E-08 | -0.024 | 0.025 | 3.33E-01 | TRUE | 3.45E-02 |
| rs1346841 | 4 | 65651730 | 0.41 | A | G | -0.013 | 0.002 | 3.18E-13 | -0.002 | 0.026 | 9.29E-01 | TRUE | 1.99E-04 |
| rs1350430 | 12 | 41819215 | 0.53 | C | T | -0.013 | 0.002 | 2.08E-13 | 0.020 | 0.024 | 4.01E-01 | TRUE | 2.32E-03 |
| rs1356506 | 18 | 40708038 | 0.63 | C | T | 0.014 | 0.002 | 8.39E-15 | 0.014 | 0.024 | 5.70E-01 | TRUE | 6.47E-04 |
| rs1358980 | 6 | 43764551 | 0.47 | T | C | -0.013 | 0.002 | 5.15E-15 | 0.051 | 0.024 | 3.26E-02 | TRUE | 4.02E-02 |
| rs1383592 | 8 | 106430676 | 0.21 | A | G | 0.012 | 0.002 | 4.92E-09 | 0.023 | 0.026 | 3.82E-01 | TRUE | 2.60E-02 |
| rs1409818 | 20 | 21381121 | 0.11 | T | C | 0.020 | 0.003 | 2.59E-12 | 0.069 | 0.048 | 1.52E-01 | TRUE | 1.94E-02 |
| rs1412235 | 9 | 28410996 | 0.32 | C | G | 0.024 | 0.002 | 2.28E-42 | 0.056 | 0.025 | 2.20E-02 | TRUE | 2.29E-07 |
| rs1421334 | 8 | 30865733 | 0.45 | C | A | 0.014 | 0.002 | 3.11E-15 | -0.024 | 0.024 | 3.30E-01 | TRUE | 1.23E-03 |
| rs1437842 | 4 | 173597016 | 0.49 | A | G | -0.011 | 0.002 | 8.49E-10 | -0.016 | 0.024 | 5.17E-01 | TRUE | 8.35E-03 |
| rs1441264 | 13 | 79580919 | 0.58 | A | G | 0.017 | 0.002 | 7.60E-25 | 0.016 | 0.025 | 5.28E-01 | TRUE | 2.58E-06 |
| rs1451077 | 2 | 147901207 | 0.58 | A | G | -0.017 | 0.002 | 1.43E-18 | -0.025 | 0.024 | 2.90E-01 | TRUE | 2.64E-04 |
| rs147568678 | 10 | 93061851 | 0.77 | C | T | 0.013 | 0.002 | 3.42E-09 | 0.030 | 0.027 | 2.78E-01 | TRUE | 4.01E-02 |
| rs1477199 | 16 | 53712135 | 0.84 | G | A | -0.022 | 0.002 | 5.15E-21 | -0.028 | 0.038 | 4.57E-01 | TRUE | 1.83E-05 |
| rs1492014 | 3 | 94071481 | 0.57 | C | T | -0.017 | 0.002 | 1.44E-23 | 0.005 | 0.024 | 8.36E-01 | TRUE | 6.52E-07 |
| rs1492767 | 4 | 55221467 | 0.47 | T | C | 0.010 | 0.002 | 3.55E-09 | 0.023 | 0.024 | 3.44E-01 | TRUE | 2.58E-02 |
| rs1501673 | 5 | 87963600 | 0.14 | A | G | 0.029 | 0.003 | 2.73E-31 | 0.084 | 0.038 | 2.91E-02 | TRUE | 5.17E-05 |
| rs150215901 | 5 | 50935903 | 0.04 | A | T | -0.028 | 0.005 | 1.54E-08 | -0.061 | 0.076 | 4.25E-01 | TRUE | 2.85E-02 |
| rs1522569 | 4 | 171632637 | 0.82 | G | T | 0.014 | 0.002 | 1.60E-10 | -0.002 | 0.042 | 9.56E-01 | TRUE | 1.21E-03 |
| rs1559673 | 15 | 62156514 | 0.97 | C | A | 0.036 | 0.005 | 2.21E-13 | -0.057 | 0.039 | 1.42E-01 | TRUE | 1.26E-02 |
| rs156201 | 6 | 104847441 | 0.75 | C | G | 0.012 | 0.002 | 1.65E-10 | -0.010 | 0.025 | 6.97E-01 | TRUE | 2.62E-03 |
| rs16851483 | 3 | 141275436 | 0.07 | T | G | 0.035 | 0.003 | 4.87E-25 | 0.000 | 0.055 | 9.98E-01 | TRUE | 1.15E-07 |
| rs16906838 | 8 | 138213836 | 0.05 | T | C | -0.025 | 0.004 | 4.31E-10 | 0.016 | 0.053 | 7.69E-01 | TRUE | 3.60E-03 |
| rs1700082 | 8 | 4121727 | 0.66 | C | G | 0.009 | 0.002 | 3.38E-08 | 0.000 | 0.025 | 9.95E-01 | TRUE | 4.69E-03 |
| rs17020497 | 2 | 81826131 | 0.13 | A | G | 0.014 | 0.003 | 2.04E-08 | 0.037 | 0.038 | 3.39E-01 | TRUE | 4.06E-02 |
| rs17024393 | 1 | 110154688 | 0.97 | C | T | -0.064 | 0.005 | 7.11E-39 | 0.128 | 0.052 | 1.44E-02 | TRUE | 3.64E-06 |
| rs17094222 | 10 | 102395440 | 0.79 | C | T | -0.017 | 0.002 | 4.04E-18 | 0.029 | 0.028 | 2.96E-01 | TRUE | 4.10E-04 |
| rs17182027 | 14 | 73348130 | 0.56 | A | G | -0.011 | 0.002 | 1.46E-10 | 0.002 | 0.024 | 9.27E-01 | TRUE | 1.21E-03 |
| rs17207196 | 7 | 75101065 | 0.42 | T | C | -0.022 | 0.002 | 1.58E-36 | 0.005 | 0.024 | 8.25E-01 | TRUE | 1.20E-10 |
| rs1721447 | 7 | 109214139 | 0.51 | G | T | -0.010 | 0.002 | 3.79E-09 | -0.009 | 0.024 | 6.95E-01 | TRUE | 7.42E-03 |
| rs17367750 | 4 | 140782542 | 0.31 | T | C | -0.012 | 0.002 | 1.97E-11 | -0.018 | 0.027 | 5.05E-01 | TRUE | 3.74E-03 |
| rs17405603 | 5 | 144608340 | 0.71 | T | A | -0.013 | 0.002 | 4.43E-11 | -0.026 | 0.026 | 3.18E-01 | TRUE | 1.20E-02 |
| rs17405819 | 8 | 76806584 | 0.68 | C | T | 0.021 | 0.002 | 6.04E-33 | -0.032 | 0.026 | 2.20E-01 | TRUE | 7.37E-07 |
| rs1750307 | 1 | 156488420 | 0.37 | A | T | 0.013 | 0.002 | 2.78E-13 | 0.011 | 0.027 | 6.92E-01 | TRUE | 8.62E-04 |
| rs17544384 | 1 | 115295160 | 0.80 | C | T | -0.013 | 0.002 | 2.28E-08 | -0.065 | 0.030 | 3.25E-02 | TRUE | 2.79E-01 |
| rs17636031 | 10 | 126594078 | 0.72 | C | T | -0.015 | 0.002 | 3.87E-17 | 0.001 | 0.029 | 9.65E-01 | TRUE | 1.39E-05 |
| rs17681451 | 3 | 114399296 | 0.08 | A | G | -0.023 | 0.003 | 7.37E-13 | -0.107 | 0.049 | 3.04E-02 | TRUE | 6.30E-02 |
| rs17724992 | 19 | 18454825 | 0.72 | G | A | 0.017 | 0.002 | 5.23E-21 | 0.014 | 0.028 | 6.31E-01 | TRUE | 7.38E-06 |
| rs17783165 | 18 | 63461638 | 0.67 | C | T | -0.013 | 0.002 | 2.74E-13 | -0.026 | 0.024 | 2.86E-01 | TRUE | 3.27E-03 |
| rs17806224 | 20 | 51065854 | 0.18 | A | G | -0.026 | 0.002 | 7.91E-32 | 0.033 | 0.033 | 3.10E-01 | TRUE | 2.19E-07 |
| rs17814208 | 2 | 144037998 | 0.76 | G | A | -0.013 | 0.002 | 7.24E-11 | 0.009 | 0.029 | 7.45E-01 | TRUE | 2.48E-03 |
| rs1799923 | 3 | 42306294 | 0.11 | G | A | -0.022 | 0.003 | 1.12E-17 | 0.027 | 0.039 | 4.84E-01 | TRUE | 1.38E-04 |
| rs1808629 | 8 | 73435964 | 0.67 | A | G | -0.020 | 0.002 | 2.19E-23 | -0.042 | 0.026 | 1.08E-01 | TRUE | 1.48E-04 |
| rs185350 | 19 | 34306816 | 0.49 | T | C | 0.014 | 0.002 | 9.10E-17 | -0.023 | 0.024 | 3.40E-01 | TRUE | 3.60E-04 |
| rs1860561 | 12 | 110783241 | 0.21 | A | G | 0.016 | 0.002 | 1.71E-16 | -0.008 | 0.027 | 7.68E-01 | TRUE | 5.48E-05 |
| rs1877875 | 9 | 120664469 | 0.43 | T | C | -0.011 | 0.002 | 2.51E-10 | -0.022 | 0.025 | 3.81E-01 | TRUE | 1.13E-02 |
| rs1884389 | 20 | 1410582 | 0.44 | T | C | -0.011 | 0.002 | 3.72E-10 | 0.020 | 0.024 | 4.01E-01 | TRUE | 1.13E-02 |
| rs1884897 | 20 | 6612832 | 0.37 | G | A | -0.018 | 0.002 | 2.70E-28 | -0.035 | 0.025 | 1.67E-01 | TRUE | 1.31E-05 |
| rs1927790 | 13 | 96922191 | 0.61 | C | T | -0.014 | 0.002 | 1.57E-17 | 0.021 | 0.024 | 3.77E-01 | TRUE | 1.96E-04 |
| rs1928295 | 9 | 120378483 | 0.57 | C | T | 0.013 | 0.002 | 2.23E-16 | -0.021 | 0.024 | 3.84E-01 | TRUE | 3.96E-04 |
| rs1941213 | 11 | 133125329 | 0.71 | C | A | 0.011 | 0.002 | 1.59E-08 | -0.041 | 0.027 | 1.31E-01 | TRUE | 1.06E-01 |
| rs1941696 | 18 | 31252129 | 0.52 | A | G | 0.011 | 0.002 | 4.74E-11 | 0.031 | 0.024 | 1.93E-01 | TRUE | 2.40E-02 |
| rs1945160 | 18 | 22164216 | 0.38 | A | G | -0.010 | 0.002 | 5.65E-09 | 0.024 | 0.025 | 3.30E-01 | TRUE | 3.37E-02 |
| rs1948080 | 9 | 11852043 | 0.63 | G | T | 0.014 | 0.002 | 1.13E-14 | -0.001 | 0.026 | 9.58E-01 | TRUE | 1.31E-04 |
| rs194809 | 16 | 23804956 | 0.19 | A | G | 0.013 | 0.002 | 4.86E-09 | -0.050 | 0.033 | 1.28E-01 | TRUE | 1.04E-01 |
| rs1951455 | 14 | 91512339 | 0.28 | C | T | -0.015 | 0.002 | 6.05E-15 | -0.028 | 0.027 | 3.00E-01 | TRUE | 1.93E-03 |
| rs1958898 | 14 | 40886886 | 0.21 | C | G | -0.015 | 0.002 | 4.23E-12 | 0.009 | 0.027 | 7.37E-01 | TRUE | 1.06E-03 |
| rs1965529 | 7 | 77825707 | 0.77 | G | A | 0.016 | 0.002 | 7.66E-14 | -0.023 | 0.031 | 4.71E-01 | TRUE | 1.75E-03 |
| rs197374 | 1 | 112289983 | 0.40 | T | C | 0.014 | 0.002 | 3.16E-16 | -0.023 | 0.024 | 3.45E-01 | TRUE | 5.85E-04 |
| rs1999433 | 9 | 81371441 | 0.44 | T | C | -0.011 | 0.002 | 2.90E-10 | -0.041 | 0.024 | 8.25E-02 | TRUE | 8.30E-02 |
| rs2007518 | 11 | 132639606 | 0.56 | G | A | -0.013 | 0.002 | 1.96E-14 | -0.027 | 0.025 | 2.77E-01 | TRUE | 2.85E-03 |
| rs2047648 | 3 | 157033438 | 0.75 | T | A | -0.013 | 0.002 | 7.95E-12 | -0.022 | 0.031 | 4.77E-01 | TRUE | 5.18E-03 |
| rs2051559 | 4 | 3298800 | 0.86 | C | T | -0.017 | 0.003 | 3.78E-11 | 0.040 | 0.038 | 2.87E-01 | TRUE | 1.22E-02 |
| rs2053682 | 5 | 170599327 | 0.68 | A | C | 0.017 | 0.002 | 2.59E-20 | 0.029 | 0.025 | 2.47E-01 | TRUE | 1.21E-04 |
| rs2058527 | 16 | 6704749 | 0.27 | G | T | -0.012 | 0.002 | 1.80E-09 | 0.001 | 0.028 | 9.79E-01 | TRUE | 2.08E-03 |
| rs2064044 | 21 | 22119890 | 0.81 | A | C | -0.012 | 0.002 | 1.02E-08 | -0.004 | 0.027 | 8.69E-01 | TRUE | 4.25E-03 |
| rs2065418 | 11 | 30422068 | 0.65 | G | T | 0.014 | 0.002 | 6.07E-15 | -0.040 | 0.026 | 1.21E-01 | TRUE | 8.64E-03 |
| rs2066295 | 6 | 26168903 | 0.76 | G | A | 0.014 | 0.002 | 2.29E-12 | 0.004 | 0.028 | 8.84E-01 | TRUE | 4.45E-04 |
| rs2074314 | 11 | 17411821 | 0.64 | T | C | 0.011 | 0.002 | 1.37E-09 | -0.044 | 0.024 | 6.32E-02 | TRUE | 1.17E-01 |
| rs2112347 | 5 | 75015242 | 0.63 | G | T | 0.028 | 0.002 | 1.17E-61 | -0.065 | 0.024 | 7.24E-03 | TRUE | 1.85E-09 |
| rs2119753 | 2 | 151224579 | 0.62 | G | A | 0.010 | 0.002 | 1.00E-08 | -0.007 | 0.024 | 7.71E-01 | TRUE | 5.73E-03 |
| rs2120710 | 8 | 93210803 | 0.65 | G | A | 0.010 | 0.002 | 1.98E-08 | -0.062 | 0.025 | 1.18E-02 | TRUE | 4.77E-01 |
| rs2134858 | 9 | 73837155 | 0.51 | T | C | -0.012 | 0.002 | 5.87E-12 | -0.049 | 0.024 | 3.82E-02 | TRUE | 8.09E-02 |
| rs213518 | 7 | 26941065 | 0.85 | C | T | -0.015 | 0.002 | 2.49E-10 | 0.007 | 0.037 | 8.41E-01 | TRUE | 1.98E-03 |
| rs214249 | 16 | 348687 | 0.61 | G | T | 0.014 | 0.002 | 2.72E-15 | 0.036 | 0.025 | 1.49E-01 | TRUE | 3.49E-03 |
| rs215669 | 7 | 32378979 | 0.60 | A | G | -0.015 | 0.002 | 8.94E-18 | -0.018 | 0.026 | 5.00E-01 | TRUE | 9.21E-05 |
| rs217433 | 7 | 44553496 | 0.80 | C | T | -0.012 | 0.002 | 3.00E-08 | -0.012 | 0.030 | 6.79E-01 | TRUE | 1.43E-02 |
| rs217669 | 14 | 62360075 | 0.72 | C | T | -0.017 | 0.002 | 6.27E-16 | 0.018 | 0.029 | 5.42E-01 | TRUE | 2.40E-04 |
| rs2192158 | 4 | 55505360 | 0.45 | G | A | 0.014 | 0.002 | 7.42E-16 | 0.019 | 0.024 | 4.21E-01 | TRUE | 5.88E-04 |
| rs2196618 | 8 | 85089437 | 0.26 | G | A | -0.014 | 0.002 | 1.49E-12 | -0.004 | 0.027 | 8.70E-01 | TRUE | 3.79E-04 |
| rs2206277 | 6 | 50798526 | 0.16 | T | C | 0.041 | 0.002 | 1.82E-83 | 0.035 | 0.029 | 2.23E-01 | TRUE | 5.30E-19 |
| rs2228213 | 6 | 12124855 | 0.34 | A | G | -0.014 | 0.002 | 5.50E-17 | -0.003 | 0.025 | 9.05E-01 | TRUE | 2.26E-05 |
| rs2228552 | 1 | 32165495 | 0.57 | T | G | 0.012 | 0.002 | 1.80E-11 | 0.003 | 0.025 | 8.94E-01 | TRUE | 1.24E-03 |
| rs2238799 | 22 | 20109325 | 0.62 | G | A | 0.010 | 0.002 | 8.65E-09 | -0.072 | 0.024 | 2.56E-03 | TRUE | 7.55E-01 |
| rs2241423 | 15 | 68086838 | 0.23 | A | G | -0.030 | 0.002 | 3.60E-54 | -0.113 | 0.033 | 6.16E-04 | TRUE | 3.51E-07 |
| rs2246012 | 6 | 131898208 | 0.85 | C | T | -0.016 | 0.002 | 1.15E-13 | 0.053 | 0.028 | 6.17E-02 | TRUE | 3.20E-02 |
| rs2257791 | 10 | 118643670 | 0.76 | A | G | -0.014 | 0.002 | 1.63E-12 | -0.035 | 0.029 | 2.32E-01 | TRUE | 1.13E-02 |
| rs225882 | 14 | 30480123 | 0.75 | T | C | 0.011 | 0.002 | 8.56E-10 | 0.030 | 0.025 | 2.27E-01 | TRUE | 2.94E-02 |
| rs2267958 | 9 | 131015279 | 0.51 | A | G | -0.013 | 0.002 | 2.01E-13 | -0.030 | 0.024 | 2.07E-01 | TRUE | 8.88E-03 |
| rs2271046 | 4 | 52752812 | 0.69 | T | A | -0.012 | 0.002 | 3.03E-10 | -0.039 | 0.026 | 1.41E-01 | TRUE | 4.46E-02 |
| rs2271189 | 12 | 56494991 | 0.40 | A | G | -0.014 | 0.002 | 9.26E-16 | 0.046 | 0.024 | 6.00E-02 | TRUE | 1.65E-02 |
| rs2273175 | 14 | 104160141 | 0.68 | C | T | -0.012 | 0.002 | 3.69E-11 | -0.009 | 0.024 | 7.10E-01 | TRUE | 1.78E-03 |
| rs2275003 | 9 | 34124860 | 0.50 | G | A | 0.011 | 0.002 | 9.44E-12 | -0.032 | 0.024 | 1.84E-01 | TRUE | 1.59E-02 |
| rs2281819 | 6 | 33771673 | 0.23 | A | T | -0.015 | 0.002 | 2.09E-14 | -0.077 | 0.026 | 3.18E-03 | TRUE | 1.58E-01 |
| rs2283006 | 7 | 93085722 | 0.49 | A | G | 0.013 | 0.002 | 8.06E-15 | 0.033 | 0.024 | 1.62E-01 | TRUE | 5.47E-03 |
| rs2283093 | 7 | 126721231 | 0.19 | T | C | 0.012 | 0.002 | 1.10E-08 | 0.026 | 0.030 | 3.86E-01 | TRUE | 2.73E-02 |
| rs2289379 | 7 | 44804225 | 0.39 | T | C | -0.014 | 0.002 | 7.02E-15 | 0.024 | 0.024 | 3.20E-01 | TRUE | 2.32E-03 |
| rs2342892 | 16 | 24540806 | 0.49 | G | T | 0.013 | 0.002 | 1.33E-13 | 0.011 | 0.024 | 6.49E-01 | TRUE | 6.59E-04 |
| rs2357760 | 6 | 120213880 | 0.67 | A | G | 0.014 | 0.002 | 2.11E-16 | -0.023 | 0.025 | 3.52E-01 | TRUE | 4.48E-04 |
| rs2365389 | 3 | 61236462 | 0.40 | T | C | -0.017 | 0.002 | 6.49E-25 | -0.070 | 0.024 | 3.64E-03 | TRUE | 3.95E-03 |
| rs2396625 | 7 | 113028634 | 0.42 | A | T | -0.018 | 0.002 | 2.81E-24 | -0.057 | 0.025 | 2.01E-02 | TRUE | 9.39E-04 |
| rs2400414 | 1 | 194965200 | 0.35 | T | C | -0.013 | 0.002 | 5.52E-13 | -0.015 | 0.025 | 5.58E-01 | TRUE | 2.05E-03 |
| rs2423668 | 20 | 12430673 | 0.42 | C | T | 0.011 | 0.002 | 7.84E-09 | -0.030 | 0.024 | 2.16E-01 | TRUE | 5.08E-02 |
| rs2436728 | 6 | 40365601 | 0.41 | A | G | 0.019 | 0.002 | 1.97E-29 | 0.042 | 0.024 | 8.52E-02 | TRUE | 2.47E-05 |
| rs2439823 | 10 | 99778226 | 0.45 | G | A | -0.017 | 0.002 | 6.51E-22 | 0.011 | 0.024 | 6.58E-01 | TRUE | 4.39E-06 |
| rs2466103 | 8 | 32412304 | 0.69 | G | T | -0.012 | 0.002 | 8.00E-12 | 0.072 | 0.028 | 1.10E-02 | TRUE | 2.07E-01 |
| rs2470893 | 15 | 75019449 | 0.30 | T | C | 0.011 | 0.002 | 9.43E-10 | 0.047 | 0.027 | 7.53E-02 | TRUE | 8.97E-02 |
| rs2503185 | 1 | 66461401 | 0.51 | G | A | 0.013 | 0.002 | 1.33E-14 | 0.031 | 0.024 | 2.01E-01 | TRUE | 4.81E-03 |
| rs2513999 | 11 | 103019633 | 0.16 | G | A | -0.015 | 0.003 | 3.07E-09 | 0.034 | 0.047 | 4.64E-01 | TRUE | 1.62E-02 |
| rs2600226 | 3 | 12928762 | 0.67 | T | C | -0.012 | 0.002 | 1.42E-10 | -0.005 | 0.025 | 8.29E-01 | TRUE | 1.83E-03 |
| rs2605603 | 11 | 93221105 | 0.48 | A | G | -0.010 | 0.002 | 2.04E-10 | -0.059 | 0.024 | 1.24E-02 | TRUE | 2.50E-01 |
| rs2622274 | 6 | 64240516 | 0.46 | T | G | -0.011 | 0.002 | 3.23E-10 | 0.038 | 0.024 | 1.13E-01 | TRUE | 6.22E-02 |
| rs264941 | 2 | 104297420 | 0.47 | A | C | -0.012 | 0.002 | 1.84E-13 | 0.020 | 0.024 | 4.03E-01 | TRUE | 2.54E-03 |
| rs2707183 | 12 | 116957607 | 0.53 | T | G | -0.009 | 0.002 | 4.89E-08 | -0.005 | 0.024 | 8.23E-01 | TRUE | 9.04E-03 |
| rs2712665 | 12 | 99594947 | 0.70 | T | C | -0.011 | 0.002 | 5.40E-09 | -0.008 | 0.027 | 7.56E-01 | TRUE | 8.17E-03 |
| rs2715423 | 15 | 99511873 | 0.28 | A | G | -0.012 | 0.002 | 1.90E-09 | -0.003 | 0.026 | 9.20E-01 | TRUE | 2.58E-03 |
| rs273512 | 19 | 18224729 | 0.41 | T | C | 0.016 | 0.002 | 4.48E-19 | 0.030 | 0.024 | 2.14E-01 | TRUE | 7.41E-04 |
| rs2744974 | 6 | 34579431 | 0.32 | T | C | 0.026 | 0.002 | 1.28E-51 | -0.029 | 0.025 | 2.56E-01 | TRUE | 5.62E-12 |
| rs274628 | 7 | 86265855 | 0.34 | C | A | -0.010 | 0.002 | 1.36E-08 | 0.039 | 0.024 | 1.11E-01 | TRUE | 1.25E-01 |
| rs2777768 | 9 | 84186734 | 0.72 | G | A | 0.012 | 0.002 | 6.38E-10 | 0.032 | 0.029 | 2.81E-01 | TRUE | 2.24E-02 |
| rs2820295 | 1 | 201800868 | 0.33 | A | G | 0.024 | 0.002 | 5.56E-39 | 0.017 | 0.026 | 5.08E-01 | TRUE | 9.34E-10 |
| rs2832283 | 21 | 30690558 | 0.23 | A | G | 0.012 | 0.002 | 4.72E-09 | 0.019 | 0.027 | 4.87E-01 | TRUE | 1.89E-02 |
| rs28350 | 3 | 42418446 | 0.17 | G | A | 0.017 | 0.002 | 1.07E-14 | -0.030 | 0.030 | 3.29E-01 | TRUE | 1.54E-03 |
| rs28489620 | 22 | 41804716 | 0.28 | A | G | -0.015 | 0.002 | 1.42E-12 | 0.032 | 0.026 | 2.25E-01 | TRUE | 8.27E-03 |
| rs2861089 | 5 | 164557954 | 0.38 | T | A | 0.011 | 0.002 | 1.55E-09 | 0.019 | 0.025 | 4.43E-01 | TRUE | 1.22E-02 |
| rs2861685 | 2 | 67837553 | 0.59 | C | T | 0.017 | 0.002 | 7.82E-18 | -0.015 | 0.024 | 5.23E-01 | TRUE | 9.60E-05 |
| rs2862996 | 11 | 43653833 | 0.70 | T | G | -0.022 | 0.002 | 3.60E-35 | -0.056 | 0.026 | 2.89E-02 | TRUE | 3.61E-06 |
| rs2875762 | 6 | 124925032 | 0.25 | C | G | 0.013 | 0.002 | 1.07E-10 | 0.038 | 0.038 | 3.22E-01 | TRUE | 1.41E-02 |
| rs2907948 | 7 | 150638484 | 0.24 | A | G | -0.015 | 0.002 | 1.95E-14 | -0.051 | 0.032 | 1.05E-01 | TRUE | 1.18E-02 |
| rs2910026 | 5 | 152529936 | 0.72 | T | C | -0.013 | 0.002 | 5.69E-10 | 0.037 | 0.028 | 1.84E-01 | TRUE | 3.76E-02 |
| rs2962334 | 5 | 86879056 | 0.03 | T | G | 0.040 | 0.006 | 1.78E-11 | 0.003 | 0.081 | 9.74E-01 | TRUE | 6.47E-04 |
| rs2984618 | 1 | 47690438 | 0.44 | T | G | 0.017 | 0.002 | 3.80E-24 | 0.011 | 0.025 | 6.58E-01 | TRUE | 9.46E-07 |
| rs3019466 | 11 | 92476178 | 0.17 | T | C | -0.013 | 0.002 | 4.91E-08 | -0.015 | 0.038 | 6.96E-01 | TRUE | 1.66E-02 |
| rs305256 | 8 | 137568252 | 0.23 | C | T | -0.012 | 0.002 | 2.79E-08 | -0.022 | 0.028 | 4.30E-01 | TRUE | 3.33E-02 |
| rs3101336 | 1 | 72751185 | 0.38 | C | T | -0.025 | 0.002 | 4.80E-54 | -0.006 | 0.025 | 8.09E-01 | TRUE | 2.28E-15 |
| rs312750 | 17 | 68343539 | 0.50 | A | G | 0.010 | 0.002 | 2.57E-09 | 0.018 | 0.024 | 4.69E-01 | TRUE | 1.30E-02 |
| rs321237 | 1 | 96478125 | 0.75 | G | A | 0.013 | 0.002 | 1.49E-10 | -0.058 | 0.027 | 3.13E-02 | TRUE | 1.68E-01 |
| rs326893 | 4 | 112691776 | 0.58 | T | C | 0.012 | 0.002 | 1.89E-12 | 0.075 | 0.025 | 3.31E-03 | TRUE | 2.61E-01 |
| rs329651 | 11 | 133767622 | 0.81 | T | G | 0.016 | 0.002 | 2.13E-14 | 0.037 | 0.041 | 3.63E-01 | TRUE | 1.79E-03 |
| rs337637 | 4 | 38604470 | 0.36 | A | G | -0.014 | 0.002 | 6.24E-16 | -0.008 | 0.025 | 7.59E-01 | TRUE | 1.11E-04 |
| rs339991 | 15 | 60913637 | 0.42 | G | A | -0.013 | 0.002 | 3.51E-13 | 0.015 | 0.024 | 5.36E-01 | TRUE | 1.22E-03 |
| rs34184235 | 3 | 86192846 | 0.44 | T | C | -0.012 | 0.002 | 2.14E-09 | -0.054 | 0.024 | 2.74E-02 | TRUE | 2.28E-01 |
| rs34234296 | 2 | 175166636 | 0.39 | A | G | -0.015 | 0.002 | 2.01E-13 | 0.048 | 0.026 | 6.70E-02 | TRUE | 3.22E-02 |
| rs34517439 | 1 | 78450517 | 0.12 | A | C | 0.039 | 0.003 | 3.40E-39 | 0.059 | 0.035 | 9.52E-02 | TRUE | 1.57E-07 |
| rs34811474 | 4 | 25408838 | 0.22 | A | G | -0.029 | 0.002 | 8.50E-38 | -0.016 | 0.028 | 5.62E-01 | TRUE | 1.66E-09 |
| rs349088 | 11 | 84814393 | 0.48 | A | C | -0.013 | 0.002 | 3.50E-14 | 0.017 | 0.025 | 4.94E-01 | TRUE | 8.67E-04 |
| rs35408866 | 4 | 187743245 | 0.13 | A | G | 0.016 | 0.003 | 1.50E-08 | -0.037 | 0.040 | 3.54E-01 | TRUE | 3.45E-02 |
| rs35867081 | 17 | 79047278 | 0.49 | G | A | 0.015 | 0.002 | 6.65E-15 | 0.026 | 0.024 | 2.77E-01 | TRUE | 1.87E-03 |
| rs35949039 | 16 | 70572605 | 0.11 | T | G | -0.022 | 0.003 | 6.30E-12 | -0.098 | 0.039 | 1.25E-02 | TRUE | 1.59E-01 |
| rs3732927 | 3 | 170586057 | 0.29 | T | C | 0.010 | 0.002 | 1.13E-08 | -0.010 | 0.027 | 7.01E-01 | TRUE | 7.96E-03 |
| rs3764625 | 19 | 49649051 | 0.40 | G | T | 0.010 | 0.002 | 2.42E-08 | 0.010 | 0.024 | 6.67E-01 | TRUE | 1.16E-02 |
| rs3764835 | 2 | 159519368 | 0.15 | A | G | -0.013 | 0.002 | 3.84E-08 | -0.002 | 0.031 | 9.55E-01 | TRUE | 6.41E-03 |
| rs3770890 | 2 | 36657992 | 0.97 | G | T | -0.030 | 0.005 | 2.10E-08 | -0.028 | 0.161 | 8.61E-01 | TRUE | 6.51E-03 |
| rs3796432 | 4 | 96030402 | 0.37 | T | G | -0.011 | 0.002 | 2.21E-10 | -0.016 | 0.026 | 5.30E-01 | TRUE | 7.43E-03 |
| rs3806114 | 6 | 20482335 | 0.69 | A | G | -0.012 | 0.002 | 1.46E-11 | -0.015 | 0.028 | 5.79E-01 | TRUE | 3.30E-03 |
| rs3808477 | 8 | 116670347 | 0.27 | T | C | -0.018 | 0.002 | 8.73E-22 | -0.034 | 0.026 | 1.81E-01 | TRUE | 1.71E-04 |
| rs3814883 | 16 | 29994922 | 0.48 | T | C | 0.023 | 0.002 | 1.47E-40 | 0.012 | 0.024 | 6.17E-01 | TRUE | 1.45E-10 |
| rs3825061 | 11 | 118944675 | 0.39 | T | C | 0.014 | 0.002 | 6.15E-16 | 0.032 | 0.026 | 2.24E-01 | TRUE | 1.50E-03 |
| rs3914628 | 4 | 147438019 | 0.86 | C | T | 0.017 | 0.002 | 6.91E-13 | -0.015 | 0.031 | 6.37E-01 | TRUE | 1.07E-03 |
| rs3923783 | 17 | 1843189 | 0.18 | A | C | -0.022 | 0.002 | 4.12E-23 | 0.017 | 0.028 | 5.48E-01 | TRUE | 3.26E-06 |
| rs39654 | 3 | 173095123 | 0.45 | G | A | -0.016 | 0.002 | 1.75E-21 | -0.013 | 0.024 | 5.81E-01 | TRUE | 9.08E-06 |
| rs40067 | 5 | 107439012 | 0.17 | A | G | -0.025 | 0.002 | 9.73E-29 | -0.024 | 0.029 | 4.19E-01 | TRUE | 8.72E-07 |
| rs4017425 | 3 | 44028764 | 0.47 | T | C | -0.012 | 0.002 | 2.91E-12 | -0.052 | 0.024 | 3.32E-02 | TRUE | 8.42E-02 |
| rs4148155 | 4 | 89054667 | 0.89 | G | A | 0.019 | 0.003 | 1.34E-13 | -0.048 | 0.045 | 2.94E-01 | TRUE | 3.95E-03 |
| rs4240673 | 8 | 10787612 | 0.45 | C | T | 0.018 | 0.002 | 1.58E-26 | -0.020 | 0.024 | 4.00E-01 | TRUE | 1.06E-06 |
| rs4256980 | 11 | 8673939 | 0.33 | G | C | -0.019 | 0.002 | 8.63E-29 | -0.016 | 0.025 | 5.29E-01 | TRUE | 3.50E-07 |
| rs427943 | 21 | 46570896 | 0.43 | C | A | -0.018 | 0.002 | 3.60E-25 | -0.030 | 0.024 | 2.16E-01 | TRUE | 1.94E-05 |
| rs4286488 | 4 | 94440026 | 0.76 | A | G | 0.012 | 0.002 | 1.70E-09 | 0.008 | 0.028 | 7.82E-01 | TRUE | 4.22E-03 |
| rs4303732 | 2 | 100830040 | 0.61 | C | T | 0.017 | 0.002 | 1.26E-22 | -0.038 | 0.024 | 1.18E-01 | TRUE | 1.78E-04 |
| rs4307239 | 7 | 24354300 | 0.54 | G | A | -0.012 | 0.002 | 1.47E-11 | 0.036 | 0.024 | 1.31E-01 | TRUE | 3.01E-02 |
| rs4390583 | 16 | 81694835 | 0.57 | C | A | -0.010 | 0.002 | 1.63E-08 | 0.017 | 0.025 | 4.97E-01 | TRUE | 2.37E-02 |
| rs4430672 | 14 | 63094407 | 0.20 | C | T | 0.012 | 0.002 | 5.00E-09 | 0.031 | 0.029 | 2.83E-01 | TRUE | 3.55E-02 |
| rs4482463 | 2 | 205375909 | 0.92 | A | C | -0.031 | 0.003 | 4.85E-23 | -0.040 | 0.051 | 4.31E-01 | TRUE | 8.71E-06 |
| rs4517716 | 14 | 83013876 | 0.79 | G | C | -0.012 | 0.002 | 4.27E-09 | 0.031 | 0.031 | 3.08E-01 | TRUE | 3.38E-02 |
| rs45486197 | 19 | 2244849 | 0.06 | A | G | 0.028 | 0.004 | 1.80E-12 | 0.081 | 0.050 | 1.08E-01 | TRUE | 2.82E-02 |
| rs459552 | 5 | 112176756 | 0.77 | A | T | -0.013 | 0.002 | 8.36E-12 | 0.037 | 0.026 | 1.57E-01 | TRUE | 1.77E-02 |
| rs4653017 | 1 | 33776728 | 0.67 | T | C | 0.012 | 0.002 | 1.11E-10 | 0.035 | 0.025 | 1.58E-01 | TRUE | 3.18E-02 |
| rs4655141 | 1 | 23312025 | 0.84 | T | C | -0.017 | 0.002 | 6.20E-15 | -0.001 | 0.037 | 9.75E-01 | TRUE | 6.27E-05 |
| rs4700608 | 5 | 63026280 | 0.52 | C | T | -0.016 | 0.002 | 4.32E-20 | 0.022 | 0.024 | 3.52E-01 | TRUE | 1.08E-04 |
| rs4721089 | 7 | 1872921 | 0.78 | T | C | 0.017 | 0.002 | 5.60E-13 | -0.005 | 0.027 | 8.45E-01 | TRUE | 3.82E-04 |
| rs4740383 | 9 | 133783566 | 0.42 | A | G | 0.013 | 0.002 | 7.03E-14 | 0.021 | 0.024 | 3.78E-01 | TRUE | 2.96E-03 |
| rs4740619 | 9 | 15634326 | 0.54 | C | T | 0.019 | 0.002 | 3.15E-31 | -0.018 | 0.024 | 4.57E-01 | TRUE | 6.19E-08 |
| rs474605 | 18 | 39612720 | 0.47 | G | A | -0.013 | 0.002 | 2.72E-13 | 0.009 | 0.024 | 7.16E-01 | TRUE | 5.50E-04 |
| rs4771218 | 13 | 28655311 | 0.62 | A | G | -0.014 | 0.002 | 3.18E-15 | 0.009 | 0.024 | 7.14E-01 | TRUE | 2.17E-04 |
| rs478707 | 18 | 7543207 | 0.20 | T | C | -0.015 | 0.002 | 1.98E-10 | -0.070 | 0.035 | 4.47E-02 | TRUE | 1.40E-01 |
| rs4812405 | 20 | 35276585 | 0.08 | A | C | -0.020 | 0.003 | 1.28E-09 | -0.061 | 0.077 | 4.30E-01 | TRUE | 1.40E-02 |
| rs4813619 | 20 | 2815715 | 0.53 | T | G | -0.011 | 0.002 | 1.70E-09 | -0.031 | 0.024 | 2.01E-01 | TRUE | 5.51E-02 |
| rs4858193 | 3 | 20441050 | 0.72 | C | T | 0.013 | 0.002 | 2.44E-12 | -0.018 | 0.027 | 4.96E-01 | TRUE | 2.69E-03 |
| rs4864201 | 4 | 130731284 | 0.35 | C | T | 0.014 | 0.002 | 4.30E-16 | 0.003 | 0.025 | 8.96E-01 | TRUE | 5.91E-05 |
| rs4865796 | 5 | 53272664 | 0.70 | A | G | -0.010 | 0.002 | 4.21E-08 | 0.037 | 0.025 | 1.42E-01 | TRUE | 1.41E-01 |
| rs4880341 | 10 | 133992689 | 0.57 | T | C | -0.013 | 0.002 | 3.06E-14 | -0.061 | 0.024 | 9.98E-03 | TRUE | 8.82E-02 |
| rs4900714 | 14 | 47302219 | 0.48 | G | T | 0.015 | 0.002 | 1.00E-18 | -0.032 | 0.024 | 1.88E-01 | TRUE | 7.01E-04 |
| rs4906263 | 14 | 103249127 | 0.66 | C | G | -0.018 | 0.002 | 8.11E-23 | -0.044 | 0.025 | 8.13E-02 | TRUE | 4.44E-04 |
| rs4921301 | 5 | 159984492 | 0.21 | T | C | -0.013 | 0.002 | 4.03E-08 | -0.011 | 0.029 | 7.18E-01 | TRUE | 1.45E-02 |
| rs4970991 | 1 | 151004003 | 0.22 | C | T | 0.012 | 0.002 | 1.81E-08 | -0.026 | 0.028 | 3.47E-01 | TRUE | 4.31E-02 |
| rs4973618 | 2 | 229002620 | 0.66 | G | A | -0.015 | 0.002 | 1.25E-16 | 0.021 | 0.026 | 4.08E-01 | TRUE | 4.61E-04 |
| rs4981693 | 14 | 29680331 | 0.77 | A | G | 0.020 | 0.002 | 7.89E-24 | 0.009 | 0.025 | 7.11E-01 | TRUE | 1.20E-06 |
| rs4986044 | 17 | 21261560 | 0.46 | T | C | -0.018 | 0.002 | 1.27E-27 | -0.047 | 0.024 | 5.01E-02 | TRUE | 6.76E-05 |
| rs4988235 | 2 | 136608646 | 0.72 | A | G | 0.012 | 0.002 | 7.09E-13 | 0.018 | 0.025 | 4.57E-01 | TRUE | 1.95E-03 |
| rs543874 | 1 | 177889480 | 0.78 | G | A | -0.048 | 0.002 | 3.06E-125 | 0.078 | 0.030 | 9.86E-03 | TRUE | 8.99E-24 |
| rs56133507 | 2 | 172818467 | 0.81 | G | T | -0.013 | 0.002 | 4.31E-08 | 0.043 | 0.036 | 2.27E-01 | TRUE | 7.85E-02 |
| rs56151256 | 15 | 78024806 | 0.76 | C | A | 0.017 | 0.002 | 6.19E-14 | 0.008 | 0.030 | 7.78E-01 | TRUE | 2.90E-04 |
| rs56161855 | 17 | 46288649 | 0.87 | T | A | -0.023 | 0.003 | 1.41E-16 | 0.018 | 0.033 | 5.77E-01 | TRUE | 1.54E-04 |
| rs56211164 | 7 | 158016764 | 0.24 | A | G | -0.013 | 0.002 | 8.90E-09 | -0.062 | 0.031 | 4.18E-02 | TRUE | 2.18E-01 |
| rs562664 | 11 | 63823619 | 0.19 | T | C | -0.014 | 0.002 | 4.15E-10 | 0.046 | 0.029 | 1.11E-01 | TRUE | 6.52E-02 |
| rs570463 | 11 | 28739318 | 0.33 | A | C | -0.012 | 0.002 | 4.33E-11 | -0.007 | 0.029 | 8.04E-01 | TRUE | 1.24E-03 |
| rs57989773 | 6 | 100629078 | 0.76 | C | T | -0.014 | 0.002 | 5.63E-10 | 0.026 | 0.030 | 3.76E-01 | TRUE | 1.63E-02 |
| rs587271 | 1 | 54743111 | 0.69 | T | C | 0.012 | 0.002 | 6.21E-11 | 0.013 | 0.026 | 6.28E-01 | TRUE | 2.71E-03 |
| rs592483 | 11 | 69445173 | 0.59 | T | C | -0.014 | 0.002 | 1.53E-16 | -0.020 | 0.024 | 4.05E-01 | TRUE | 6.42E-04 |
| rs59302296 | 3 | 9507314 | 0.10 | A | T | 0.022 | 0.003 | 9.12E-12 | -0.027 | 0.049 | 5.84E-01 | TRUE | 2.66E-03 |
| rs6010784 | 20 | 61540319 | 0.51 | C | T | 0.011 | 0.002 | 6.91E-11 | -0.016 | 0.024 | 5.13E-01 | TRUE | 4.62E-03 |
| rs61740466 | 1 | 19934900 | 0.23 | A | G | -0.014 | 0.002 | 2.24E-10 | -0.057 | 0.029 | 4.82E-02 | TRUE | 1.07E-01 |
| rs61813324 | 1 | 156049877 | 0.13 | T | C | 0.029 | 0.003 | 3.20E-24 | 0.021 | 0.033 | 5.12E-01 | TRUE | 2.31E-06 |
| rs61828641 | 1 | 174321997 | 0.11 | A | G | 0.022 | 0.003 | 2.49E-13 | 0.141 | 0.042 | 7.49E-04 | TRUE | 3.61E-01 |
| rs61983990 | 14 | 41475003 | 0.08 | A | G | 0.020 | 0.004 | 1.91E-08 | -0.027 | 0.063 | 6.74E-01 | TRUE | 1.12E-02 |
| rs62176243 | 2 | 166190881 | 0.76 | T | A | 0.015 | 0.002 | 2.41E-11 | -0.023 | 0.026 | 3.74E-01 | TRUE | 7.31E-03 |
| rs6265 | 11 | 27679916 | 0.18 | T | C | -0.041 | 0.002 | 7.40E-89 | -0.028 | 0.033 | 3.93E-01 | TRUE | 9.58E-21 |
| rs6443750 | 3 | 181329682 | 0.20 | C | T | -0.015 | 0.002 | 7.25E-13 | 0.008 | 0.029 | 7.84E-01 | TRUE | 5.16E-04 |
| rs6445258 | 3 | 62112198 | 0.21 | C | T | 0.013 | 0.002 | 2.48E-08 | 0.031 | 0.034 | 3.60E-01 | TRUE | 3.30E-02 |
| rs6463489 | 7 | 5542513 | 0.10 | C | T | 0.017 | 0.003 | 2.50E-10 | 0.028 | 0.046 | 5.45E-01 | TRUE | 5.59E-03 |
| rs6468266 | 8 | 34380276 | 0.42 | A | T | -0.011 | 0.002 | 7.71E-11 | -0.016 | 0.025 | 5.30E-01 | TRUE | 4.16E-03 |
| rs6470144 | 8 | 124152245 | 0.65 | G | T | 0.010 | 0.002 | 3.06E-08 | -0.030 | 0.024 | 2.26E-01 | TRUE | 8.03E-02 |
| rs6493498 | 15 | 51754451 | 0.45 | C | T | 0.014 | 0.002 | 4.84E-17 | 0.043 | 0.024 | 7.92E-02 | TRUE | 3.98E-03 |
| rs6496248 | 15 | 98262923 | 0.64 | T | A | 0.010 | 0.002 | 2.93E-09 | -0.029 | 0.024 | 2.30E-01 | TRUE | 5.37E-02 |
| rs6500208 | 16 | 49011249 | 0.20 | A | G | 0.015 | 0.002 | 3.21E-13 | 0.011 | 0.026 | 6.67E-01 | TRUE | 7.51E-04 |
| rs650198 | 12 | 69674595 | 0.73 | T | C | -0.014 | 0.002 | 6.43E-13 | -0.010 | 0.025 | 6.83E-01 | TRUE | 8.27E-04 |
| rs6512302 | 20 | 62691550 | 0.74 | C | G | 0.013 | 0.002 | 1.52E-11 | -0.041 | 0.029 | 1.59E-01 | TRUE | 2.63E-02 |
| rs6539064 | 12 | 103706754 | 0.75 | G | C | 0.019 | 0.002 | 1.14E-23 | 0.037 | 0.026 | 1.55E-01 | TRUE | 6.11E-05 |
| rs6545714 | 2 | 59307725 | 0.61 | A | G | -0.019 | 0.002 | 4.01E-32 | -0.024 | 0.024 | 3.13E-01 | TRUE | 9.03E-08 |
| rs6556301 | 5 | 176527577 | 0.37 | T | G | -0.011 | 0.002 | 8.14E-11 | 0.016 | 0.025 | 5.29E-01 | TRUE | 4.18E-03 |
| rs6567160 | 18 | 57829135 | 0.75 | C | T | -0.055 | 0.002 | 7.82E-184 | 0.104 | 0.030 | 5.17E-04 | TRUE | 1.18E-32 |
| rs657452 | 1 | 49589847 | 0.40 | G | A | 0.019 | 0.002 | 3.17E-30 | -0.028 | 0.024 | 2.52E-01 | TRUE | 4.77E-07 |
| rs6591407 | 11 | 56914157 | 0.20 | A | C | -0.012 | 0.002 | 3.58E-09 | 0.017 | 0.033 | 6.01E-01 | TRUE | 1.00E-02 |
| rs6607337 | NA | NA | 0.30 | C | T | -0.012 | 0.002 | 2.55E-11 | 0.031 | 0.028 | 2.58E-01 | TRUE | 1.77E-02 |
| rs6656785 | 1 | 75005776 | 0.61 | G | A | -0.018 | 0.002 | 3.06E-27 | -0.009 | 0.024 | 7.20E-01 | TRUE | 6.99E-08 |
| rs66595146 | 18 | 58204315 | 0.63 | C | A | 0.014 | 0.002 | 9.09E-12 | -0.037 | 0.024 | 1.26E-01 | TRUE | 3.21E-02 |
| rs6661316 | 1 | 210095527 | 0.59 | T | C | 0.012 | 0.002 | 1.72E-13 | 0.036 | 0.024 | 1.29E-01 | TRUE | 1.12E-02 |
| rs6696828 | 1 | 80812020 | 0.30 | C | G | 0.012 | 0.002 | 6.42E-11 | 0.033 | 0.029 | 2.48E-01 | TRUE | 1.53E-02 |
| rs6710091 | 2 | 239597 | 0.67 | G | C | 0.010 | 0.002 | 5.78E-09 | 0.000 | 0.025 | 9.94E-01 | TRUE | 3.45E-03 |
| rs6716898 | 2 | 198944271 | 0.49 | A | G | 0.013 | 0.002 | 2.33E-11 | 0.021 | 0.024 | 3.87E-01 | TRUE | 7.34E-03 |
| rs6720868 | 2 | 230663576 | 0.31 | T | C | 0.015 | 0.002 | 1.82E-17 | -0.005 | 0.026 | 8.34E-01 | TRUE | 2.63E-05 |
| rs6725931 | 2 | 220205146 | 0.85 | T | C | 0.019 | 0.002 | 2.30E-15 | 0.085 | 0.053 | 1.06E-01 | TRUE | 9.18E-03 |
| rs6783054 | 3 | 11672805 | 0.50 | C | A | 0.010 | 0.002 | 6.22E-09 | 0.035 | 0.024 | 1.50E-01 | TRUE | 8.05E-02 |
| rs6803161 | 3 | 196205694 | 0.39 | C | T | 0.011 | 0.002 | 2.71E-08 | -0.053 | 0.025 | 3.20E-02 | TRUE | 2.97E-01 |
| rs6804842 | 3 | 25106437 | 0.43 | G | A | -0.014 | 0.002 | 7.57E-18 | 0.056 | 0.024 | 1.80E-02 | TRUE | 1.30E-02 |
| rs6808814 | 3 | 116852469 | 0.73 | C | T | 0.012 | 0.002 | 1.31E-09 | -0.018 | 0.028 | 5.37E-01 | TRUE | 8.01E-03 |
| rs6850421 | 4 | 180187034 | 0.46 | G | A | 0.011 | 0.002 | 3.66E-09 | -0.004 | 0.024 | 8.77E-01 | TRUE | 3.57E-03 |
| rs6864049 | 5 | 124330522 | 0.49 | G | A | -0.012 | 0.002 | 1.48E-13 | 0.023 | 0.024 | 3.51E-01 | TRUE | 2.11E-03 |
| rs687339 | 3 | 135932359 | 0.77 | T | C | 0.019 | 0.002 | 4.32E-22 | 0.021 | 0.033 | 5.32E-01 | TRUE | 5.84E-06 |
| rs6882366 | 5 | 95864693 | 0.40 | T | C | -0.013 | 0.002 | 4.39E-14 | -0.034 | 0.026 | 1.88E-01 | TRUE | 4.84E-03 |
| rs6886072 | 5 | 136598460 | 0.47 | T | C | -0.010 | 0.002 | 4.13E-09 | -0.007 | 0.024 | 7.61E-01 | TRUE | 5.92E-03 |
| rs6888194 | 5 | 106910657 | 0.84 | C | T | -0.013 | 0.002 | 3.58E-08 | 0.028 | 0.033 | 3.93E-01 | TRUE | 3.62E-02 |
| rs6890310 | 5 | 27193573 | 0.29 | A | G | -0.012 | 0.002 | 3.29E-10 | -0.006 | 0.028 | 8.18E-01 | TRUE | 2.60E-03 |
| rs6893539 | 5 | 122705737 | 0.70 | A | C | -0.012 | 0.002 | 6.17E-11 | -0.003 | 0.026 | 8.96E-01 | TRUE | 1.49E-03 |
| rs6909685 | 6 | 97753952 | 0.33 | T | C | -0.015 | 0.002 | 2.78E-16 | -0.032 | 0.025 | 1.91E-01 | TRUE | 1.82E-03 |
| rs6915002 | 6 | 54028069 | 0.41 | C | T | 0.010 | 0.002 | 8.71E-09 | 0.013 | 0.025 | 5.86E-01 | TRUE | 1.19E-02 |
| rs6921533 | 6 | 73742334 | 0.29 | C | T | 0.010 | 0.002 | 1.37E-08 | 0.001 | 0.026 | 9.58E-01 | TRUE | 3.56E-03 |
| rs6922607 | 6 | 142703483 | 0.81 | G | A | -0.013 | 0.002 | 1.85E-09 | -0.012 | 0.031 | 7.10E-01 | TRUE | 6.76E-03 |
| rs6950388 | 7 | 1270699 | 0.78 | A | G | 0.014 | 0.002 | 1.59E-09 | 0.052 | 0.028 | 6.64E-02 | TRUE | 1.17E-01 |
| rs6973656 | 7 | 77422583 | 0.58 | G | A | -0.010 | 0.002 | 6.31E-09 | 0.011 | 0.025 | 6.67E-01 | TRUE | 7.50E-03 |
| rs698147 | 5 | 3513485 | 0.45 | G | A | 0.012 | 0.002 | 9.67E-12 | 0.006 | 0.024 | 8.05E-01 | TRUE | 1.03E-03 |
| rs7024334 | 9 | 109072075 | 0.23 | G | T | 0.014 | 0.002 | 4.71E-12 | -0.017 | 0.029 | 5.60E-01 | TRUE | 3.11E-03 |
| rs7025938 | 9 | 103088321 | 0.68 | G | C | -0.016 | 0.002 | 1.47E-19 | -0.043 | 0.024 | 7.97E-02 | TRUE | 1.90E-03 |
| rs7070670 | 10 | 61842645 | 0.32 | T | C | -0.013 | 0.002 | 7.14E-10 | -0.048 | 0.027 | 6.77E-02 | TRUE | 1.32E-01 |
| rs7084454 | 10 | 21821274 | 0.31 | A | G | 0.020 | 0.002 | 4.51E-27 | -0.007 | 0.026 | 7.88E-01 | TRUE | 6.52E-08 |
| rs7102454 | 11 | 65594820 | 0.64 | C | T | -0.017 | 0.002 | 3.84E-21 | 0.008 | 0.026 | 7.47E-01 | TRUE | 6.64E-06 |
| rs7124681 | 11 | 47529947 | 0.42 | A | C | 0.026 | 0.002 | 3.96E-55 | 0.013 | 0.024 | 5.92E-01 | TRUE | 7.95E-15 |
| rs7138803 | 12 | 50247468 | 0.39 | A | G | 0.030 | 0.002 | 3.10E-71 | 0.028 | 0.025 | 2.49E-01 | TRUE | 1.72E-15 |
| rs7144011 | 14 | 79940383 | 0.23 | T | G | 0.026 | 0.002 | 2.37E-40 | 0.062 | 0.028 | 2.63E-02 | TRUE | 1.37E-06 |
| rs7161194 | 14 | 101529005 | 0.34 | G | A | 0.019 | 0.002 | 2.23E-24 | -0.014 | 0.026 | 5.83E-01 | TRUE | 3.29E-06 |
| rs7171864 | 15 | 73227249 | 0.69 | A | G | 0.013 | 0.002 | 2.60E-14 | -0.002 | 0.026 | 9.41E-01 | TRUE | 9.08E-05 |
| rs7172627 | 15 | 31877690 | 0.52 | G | A | -0.011 | 0.002 | 1.81E-11 | 0.037 | 0.024 | 1.17E-01 | TRUE | 3.66E-02 |
| rs7206608 | 16 | 82872628 | 0.68 | G | C | -0.013 | 0.002 | 1.20E-12 | -0.054 | 0.025 | 2.97E-02 | TRUE | 6.69E-02 |
| rs7245985 | 19 | 30710410 | 0.80 | G | T | 0.012 | 0.002 | 1.88E-08 | -0.003 | 0.030 | 9.24E-01 | TRUE | 4.78E-03 |
| rs72649373 | 4 | 80609966 | 0.86 | C | T | -0.017 | 0.003 | 3.20E-09 | 0.014 | 0.033 | 6.77E-01 | TRUE | 6.97E-03 |
| rs72757415 | 15 | 92572762 | 0.21 | T | G | -0.016 | 0.002 | 1.34E-11 | -0.048 | 0.027 | 7.95E-02 | TRUE | 4.18E-02 |
| rs73213484 | 4 | 28489339 | 0.86 | T | A | 0.021 | 0.003 | 2.04E-14 | -0.044 | 0.034 | 1.91E-01 | TRUE | 4.76E-03 |
| rs73225274 | 8 | 21088909 | 0.86 | G | A | -0.016 | 0.003 | 2.33E-08 | 0.015 | 0.040 | 6.99E-01 | TRUE | 1.17E-02 |
| rs7323 | 13 | 28009031 | 0.27 | C | G | -0.017 | 0.002 | 2.18E-18 | -0.013 | 0.027 | 6.20E-01 | TRUE | 5.11E-05 |
| rs7357754 | 9 | 92207308 | 0.50 | G | A | -0.012 | 0.002 | 1.81E-12 | 0.021 | 0.024 | 3.66E-01 | TRUE | 4.51E-03 |
| rs73985439 | 2 | 212299249 | 0.69 | C | A | -0.013 | 0.002 | 1.84E-10 | 0.029 | 0.026 | 2.56E-01 | TRUE | 2.64E-02 |
| rs742748 | 20 | 39293397 | 0.59 | C | T | -0.011 | 0.002 | 1.13E-11 | -0.024 | 0.024 | 3.13E-01 | TRUE | 1.11E-02 |
| rs74887628 | 1 | 147032779 | 0.03 | A | G | 0.031 | 0.005 | 1.76E-08 | -0.120 | 0.100 | 2.31E-01 | TRUE | 6.21E-02 |
| rs750090 | 4 | 152931436 | 0.63 | C | T | 0.011 | 0.002 | 2.50E-10 | 0.015 | 0.024 | 5.48E-01 | TRUE | 6.93E-03 |
| rs7512146 | 1 | 34283008 | 0.53 | T | G | -0.010 | 0.002 | 1.29E-08 | -0.022 | 0.024 | 3.54E-01 | TRUE | 3.34E-02 |
| rs7534091 | 1 | 118864616 | 0.72 | G | A | -0.012 | 0.002 | 6.65E-11 | -0.003 | 0.028 | 9.09E-01 | TRUE | 9.08E-04 |
| rs7561278 | 2 | 48954905 | 0.77 | C | T | 0.017 | 0.002 | 4.89E-16 | 0.028 | 0.028 | 3.16E-01 | TRUE | 1.11E-03 |
| rs756717 | 16 | 72996162 | 0.39 | A | G | -0.013 | 0.002 | 2.38E-15 | -0.002 | 0.024 | 9.47E-01 | TRUE | 6.87E-05 |
| rs7588437 | 2 | 181575281 | 0.37 | A | G | -0.017 | 0.002 | 2.32E-22 | -0.005 | 0.026 | 8.41E-01 | TRUE | 1.59E-06 |
| rs7593917 | 2 | 203931012 | 0.45 | G | A | -0.012 | 0.002 | 9.67E-13 | 0.020 | 0.024 | 4.11E-01 | TRUE | 2.91E-03 |
| rs7599312 | 2 | 213413231 | 0.27 | A | G | -0.018 | 0.002 | 1.52E-23 | 0.035 | 0.026 | 1.77E-01 | TRUE | 5.78E-05 |
| rs7616009 | 3 | 194881756 | 0.16 | A | G | -0.016 | 0.002 | 4.33E-11 | 0.011 | 0.038 | 7.73E-01 | TRUE | 1.91E-03 |
| rs7631156 | 3 | 131751628 | 0.31 | A | G | 0.022 | 0.002 | 3.33E-32 | 0.093 | 0.026 | 2.84E-04 | TRUE | 2.68E-03 |
| rs7640424 | 3 | 107820063 | 0.31 | T | C | -0.014 | 0.002 | 1.23E-14 | -0.043 | 0.025 | 8.32E-02 | TRUE | 1.85E-02 |
| rs765125 | 12 | 2156207 | 0.59 | C | T | -0.010 | 0.002 | 1.27E-08 | 0.023 | 0.024 | 3.26E-01 | TRUE | 3.49E-02 |
| rs765875 | 6 | 143185683 | 0.47 | T | C | -0.013 | 0.002 | 1.08E-14 | -0.067 | 0.024 | 5.35E-03 | TRUE | 1.13E-01 |
| rs76638898 | 10 | 21099584 | 0.02 | A | G | -0.038 | 0.007 | 8.15E-09 | -0.146 | 0.064 | 2.29E-02 | TRUE | 3.13E-01 |
| rs7678054 | 4 | 95093855 | 0.48 | A | G | -0.010 | 0.002 | 4.57E-09 | -0.004 | 0.024 | 8.67E-01 | TRUE | 4.52E-03 |
| rs768023 | 6 | 108876002 | 0.60 | A | G | 0.016 | 0.002 | 1.13E-22 | 0.038 | 0.024 | 1.13E-01 | TRUE | 1.48E-04 |
| rs7685628 | 4 | 165310133 | 0.40 | A | T | 0.010 | 0.002 | 6.15E-09 | 0.028 | 0.025 | 2.60E-01 | TRUE | 3.79E-02 |
| rs76942203 | 11 | 116973247 | 0.06 | A | G | 0.026 | 0.004 | 9.08E-11 | 0.028 | 0.045 | 5.37E-01 | TRUE | 5.84E-03 |
| rs7696649 | 4 | 120322177 | 0.28 | A | G | 0.012 | 0.002 | 4.73E-10 | 0.037 | 0.028 | 1.86E-01 | TRUE | 4.65E-02 |
| rs7713317 | 5 | 95716722 | 0.72 | G | A | -0.017 | 0.002 | 1.96E-20 | -0.016 | 0.026 | 5.35E-01 | TRUE | 2.77E-05 |
| rs7715256 | 5 | 153537893 | 0.56 | T | G | -0.016 | 0.002 | 3.98E-22 | -0.029 | 0.024 | 2.25E-01 | TRUE | 5.89E-05 |
| rs77165542 | 2 | 430975 | 0.03 | T | C | -0.094 | 0.005 | 1.51E-70 | -0.068 | 0.092 | 4.60E-01 | TRUE | 3.13E-17 |
| rs7727781 | 5 | 165185571 | 0.52 | C | T | 0.009 | 0.002 | 4.27E-08 | 0.014 | 0.024 | 5.73E-01 | TRUE | 2.04E-02 |
| rs7730004 | 5 | 43191033 | 0.66 | T | C | 0.014 | 0.002 | 1.46E-14 | 0.020 | 0.024 | 3.94E-01 | TRUE | 1.26E-03 |
| rs7734385 | 5 | 158460212 | 0.44 | A | G | -0.010 | 0.002 | 6.08E-10 | -0.024 | 0.024 | 3.12E-01 | TRUE | 1.80E-02 |
| rs77432547 | 13 | 86494817 | 0.72 | G | A | -0.017 | 0.002 | 1.44E-15 | 0.008 | 0.025 | 7.50E-01 | TRUE | 1.07E-04 |
| rs7760482 | 6 | 147354276 | 0.62 | G | A | -0.010 | 0.002 | 6.25E-09 | 0.000 | 0.024 | 9.95E-01 | TRUE | 3.76E-03 |
| rs7774 | 17 | 4801163 | 0.32 | A | C | 0.013 | 0.002 | 4.22E-10 | -0.012 | 0.024 | 6.24E-01 | TRUE | 6.90E-03 |
| rs7802342 | 7 | 137435925 | 0.70 | G | T | -0.012 | 0.002 | 6.23E-11 | -0.005 | 0.027 | 8.42E-01 | TRUE | 1.51E-03 |
| rs7842934 | 8 | 132838921 | 0.92 | C | T | -0.018 | 0.003 | 1.55E-08 | 0.063 | 0.055 | 2.53E-01 | TRUE | 5.01E-02 |
| rs7861160 | 9 | 80799579 | 0.59 | C | T | 0.010 | 0.002 | 3.68E-08 | -0.026 | 0.024 | 2.83E-01 | TRUE | 5.23E-02 |
| rs7893571 | 10 | 16750129 | 0.67 | T | G | 0.013 | 0.002 | 5.83E-12 | -0.002 | 0.026 | 9.51E-01 | TRUE | 4.56E-04 |
| rs7899106 | 10 | 87410904 | 0.95 | G | A | -0.033 | 0.004 | 1.72E-18 | -0.097 | 0.058 | 9.24E-02 | TRUE | 2.05E-03 |
| rs7907470 | 10 | 10268989 | 0.92 | A | G | -0.018 | 0.003 | 7.36E-09 | -0.068 | 0.037 | 6.53E-02 | TRUE | 1.79E-01 |
| rs79113395 | 1 | 1590521 | 0.27 | A | G | -0.020 | 0.002 | 2.03E-20 | -0.056 | 0.029 | 5.10E-02 | TRUE | 2.87E-03 |
| rs79186842 | 20 | 47689036 | 0.86 | G | A | 0.020 | 0.003 | 2.60E-13 | 0.021 | 0.033 | 5.29E-01 | TRUE | 1.60E-03 |
| rs7944782 | 11 | 130795698 | 0.50 | G | T | -0.014 | 0.002 | 3.61E-17 | -0.007 | 0.024 | 7.55E-01 | TRUE | 4.67E-05 |
| rs7975187 | 12 | 60964108 | 0.77 | G | A | -0.014 | 0.002 | 3.86E-11 | 0.016 | 0.030 | 5.92E-01 | TRUE | 3.96E-03 |
| rs79780963 | 10 | 104952499 | 0.08 | T | C | 0.024 | 0.004 | 2.63E-12 | -0.037 | 0.043 | 3.94E-01 | TRUE | 4.51E-03 |
| rs79906980 | 1 | 57887985 | 0.16 | T | C | 0.016 | 0.003 | 1.69E-09 | 0.019 | 0.038 | 6.17E-01 | TRUE | 7.28E-03 |
| rs805412 | 2 | 54120820 | 0.43 | A | G | -0.010 | 0.002 | 1.14E-08 | 0.015 | 0.024 | 5.18E-01 | TRUE | 1.65E-02 |
| rs8057911 | 16 | 54143352 | 0.23 | T | C | 0.013 | 0.002 | 3.66E-08 | 0.074 | 0.030 | 1.33E-02 | TRUE | 5.10E-01 |
| rs8065172 | 17 | 31456969 | 0.24 | A | G | -0.012 | 0.002 | 5.29E-10 | 0.051 | 0.026 | 5.56E-02 | TRUE | 1.25E-01 |
| rs8097672 | 18 | 1839601 | 0.85 | T | A | -0.021 | 0.002 | 3.26E-18 | -0.052 | 0.031 | 9.55E-02 | TRUE | 2.45E-03 |
| rs8122855 | 20 | 25192049 | 0.34 | A | G | 0.014 | 0.002 | 4.11E-14 | 0.048 | 0.025 | 5.74E-02 | TRUE | 2.34E-02 |
| rs8126575 | 21 | 46435610 | 0.87 | G | T | 0.015 | 0.003 | 5.88E-10 | -0.040 | 0.031 | 1.97E-01 | TRUE | 4.48E-02 |
| rs8134638 | 21 | 40644170 | 0.63 | C | T | -0.013 | 0.002 | 1.57E-11 | 0.003 | 0.024 | 8.90E-01 | TRUE | 1.01E-03 |
| rs8181823 | 13 | 65477940 | 0.23 | C | A | -0.013 | 0.002 | 4.36E-10 | 0.018 | 0.027 | 5.00E-01 | TRUE | 8.73E-03 |
| rs845084 | 10 | 125220036 | 0.27 | A | G | 0.014 | 0.002 | 3.22E-12 | 0.049 | 0.027 | 7.04E-02 | TRUE | 3.46E-02 |
| rs852056 | 20 | 17102860 | 0.25 | C | T | 0.012 | 0.002 | 2.15E-10 | 0.002 | 0.028 | 9.32E-01 | TRUE | 1.18E-03 |
| rs865809 | 3 | 183997735 | 0.22 | G | A | 0.012 | 0.002 | 6.50E-10 | -0.008 | 0.026 | 7.59E-01 | TRUE | 3.58E-03 |
| rs879620 | 16 | 4015729 | 0.60 | T | C | 0.023 | 0.002 | 8.45E-39 | 0.008 | 0.024 | 7.38E-01 | TRUE | 6.88E-11 |
| rs889398 | 16 | 69556715 | 0.42 | T | C | -0.020 | 0.002 | 3.23E-32 | 0.005 | 0.024 | 8.35E-01 | TRUE | 1.32E-09 |
| rs891387 | 18 | 21103909 | 0.50 | C | T | 0.021 | 0.002 | 9.26E-35 | -0.041 | 0.024 | 8.78E-02 | TRUE | 1.57E-06 |
| rs895330 | 19 | 4060707 | 0.81 | G | C | 0.020 | 0.002 | 1.59E-19 | 0.012 | 0.032 | 7.04E-01 | TRUE | 2.25E-05 |
| rs900144 | 11 | 13294268 | 0.58 | T | C | 0.015 | 0.002 | 1.53E-18 | -0.002 | 0.024 | 9.43E-01 | TRUE | 1.09E-05 |
| rs9168 | 13 | 99101583 | 0.28 | A | C | -0.014 | 0.002 | 1.08E-13 | -0.026 | 0.027 | 3.37E-01 | TRUE | 3.47E-03 |
| rs925018 | 3 | 62713143 | 0.67 | G | C | -0.013 | 0.002 | 4.33E-14 | -0.011 | 0.027 | 6.88E-01 | TRUE | 3.53E-04 |
| rs9294260 | 6 | 83433228 | 0.47 | A | G | 0.014 | 0.002 | 8.16E-18 | -0.003 | 0.024 | 8.93E-01 | TRUE | 1.26E-05 |
| rs9299 | 17 | 46669430 | 0.64 | T | C | 0.012 | 0.002 | 1.87E-11 | 0.008 | 0.026 | 7.61E-01 | TRUE | 1.77E-03 |
| rs930295 | 2 | 50233352 | 0.15 | C | A | 0.021 | 0.002 | 2.03E-19 | -0.036 | 0.036 | 3.09E-01 | TRUE | 1.70E-04 |
| rs9304665 | 19 | 47602577 | 0.74 | A | T | 0.023 | 0.002 | 2.20E-31 | 0.023 | 0.026 | 3.89E-01 | TRUE | 2.58E-07 |
| rs9320823 | 6 | 98429337 | 0.41 | C | T | -0.017 | 0.002 | 2.07E-21 | -0.003 | 0.025 | 8.93E-01 | TRUE | 1.19E-06 |
| rs935166 | 2 | 26949366 | 0.49 | A | G | -0.015 | 0.002 | 8.69E-16 | 0.028 | 0.024 | 2.44E-01 | TRUE | 1.95E-03 |
| rs9370410 | 6 | 55171842 | 0.73 | G | A | 0.011 | 0.002 | 2.58E-08 | -0.020 | 0.027 | 4.55E-01 | TRUE | 2.85E-02 |
| rs942066 | 14 | 94031914 | 0.37 | G | A | -0.020 | 0.002 | 2.36E-24 | 0.050 | 0.025 | 4.36E-02 | TRUE | 5.79E-04 |
| rs9458814 | 6 | 163771305 | 0.77 | C | T | -0.011 | 0.002 | 2.33E-08 | -0.001 | 0.030 | 9.79E-01 | TRUE | 4.43E-03 |
| rs946824 | 1 | 243684019 | 0.14 | C | T | 0.020 | 0.003 | 4.82E-15 | -0.002 | 0.034 | 9.60E-01 | TRUE | 6.49E-05 |
| rs9478496 | 6 | 154333183 | 0.85 | C | T | -0.016 | 0.002 | 8.17E-12 | -0.023 | 0.030 | 4.37E-01 | TRUE | 4.67E-03 |
| rs9512648 | 13 | 27933910 | 0.47 | G | A | 0.010 | 0.002 | 4.11E-08 | -0.027 | 0.024 | 2.59E-01 | TRUE | 5.83E-02 |
| rs9522183 | 13 | 111977280 | 0.55 | T | G | -0.014 | 0.002 | 2.10E-12 | -0.005 | 0.024 | 8.30E-01 | TRUE | 5.49E-04 |
| rs9527895 | 13 | 59367767 | 0.82 | C | T | -0.016 | 0.002 | 3.69E-12 | 0.027 | 0.034 | 4.28E-01 | TRUE | 4.52E-03 |
| rs9531786 | 13 | 85983968 | 0.37 | C | G | -0.011 | 0.002 | 4.11E-10 | -0.012 | 0.025 | 6.23E-01 | TRUE | 6.19E-03 |
| rs9569777 | 13 | 58484786 | 0.19 | T | G | -0.020 | 0.002 | 3.43E-21 | -0.026 | 0.030 | 3.78E-01 | TRUE | 3.38E-05 |
| rs9595908 | 13 | 33184288 | 0.64 | C | T | 0.015 | 0.002 | 3.73E-20 | -0.043 | 0.024 | 7.70E-02 | TRUE | 1.80E-03 |
| rs9599161 | 13 | 67434016 | 0.57 | T | C | 0.010 | 0.002 | 2.32E-09 | 0.015 | 0.025 | 5.66E-01 | TRUE | 8.16E-03 |
| rs9603697 | 13 | 40783323 | 0.32 | T | C | 0.013 | 0.002 | 1.69E-13 | 0.009 | 0.024 | 6.95E-01 | TRUE | 5.07E-04 |
| rs962796 | 13 | 54385284 | 0.19 | C | T | 0.014 | 0.002 | 1.88E-11 | -0.039 | 0.030 | 1.93E-01 | TRUE | 2.15E-02 |
| rs9816226 | 3 | 185834499 | 0.18 | T | A | -0.032 | 0.002 | 1.45E-50 | -0.063 | 0.032 | 5.10E-02 | TRUE | 1.88E-09 |
| rs9818122 | 3 | 85861064 | 0.79 | C | T | -0.023 | 0.002 | 3.97E-30 | -0.063 | 0.031 | 4.36E-02 | TRUE | 4.00E-05 |
| rs9826775 | 3 | 156295341 | 0.85 | G | A | 0.016 | 0.002 | 6.61E-11 | 0.018 | 0.033 | 5.91E-01 | TRUE | 4.42E-03 |
| rs9827823 | 3 | 84221774 | 0.86 | C | T | 0.018 | 0.002 | 3.39E-15 | -0.017 | 0.037 | 6.51E-01 | TRUE | 2.47E-04 |
| rs9888533 | 13 | 107854612 | 0.53 | T | C | 0.013 | 0.002 | 1.38E-10 | 0.011 | 0.024 | 6.37E-01 | TRUE | 3.02E-03 |
| rs9926784 | 16 | 19941968 | 0.80 | C | T | 0.024 | 0.002 | 1.08E-30 | -0.022 | 0.031 | 4.76E-01 | TRUE | 2.35E-07 |
| rs9944219 | 15 | 46500612 | 0.60 | G | A | -0.012 | 0.002 | 8.14E-13 | 0.063 | 0.025 | 1.29E-02 | TRUE | 1.47E-01 |
| rs994596 | 4 | 18459828 | 0.32 | T | C | 0.013 | 0.002 | 1.06E-12 | -0.038 | 0.027 | 1.64E-01 | TRUE | 1.22E-02 |
| rs9951619 | 18 | 56882326 | 0.22 | G | T | -0.015 | 0.002 | 2.33E-15 | 0.042 | 0.026 | 9.62E-02 | TRUE | 7.59E-03 |
| rs9992189 | 4 | 144060728 | 0.61 | C | G | -0.010 | 0.002 | 2.04E-08 | -0.015 | 0.025 | 5.49E-01 | TRUE | 1.60E-02 |
| **adult WHR** | | | | | | | | | | | | | |
| rs10019888 | 4 | 26062990 | 0.14 | G | A | -0.021 | 0.002 | 8.05E-20 | 0.059 | 0.035 | 8.91E-02 | TRUE | 5.57E-04 |
| rs10049088 | 3 | 156797648 | 0.31 | T | C | -0.027 | 0.002 | 1.47E-53 | 0.013 | 0.026 | 6.13E-01 | TRUE | 1.33E-14 |
| rs10132280 | 14 | 25928179 | 0.35 | A | C | -0.012 | 0.002 | 3.11E-10 | -0.025 | 0.025 | 3.22E-01 | TRUE | 8.16E-03 |
| rs10144278 | 14 | 23749595 | 0.49 | A | G | 0.010 | 0.002 | 3.10E-08 | 0.030 | 0.024 | 2.00E-01 | TRUE | 5.10E-02 |
| rs10158345 | 1 | 224062306 | 0.70 | G | C | 0.012 | 0.002 | 1.57E-09 | 0.036 | 0.026 | 1.70E-01 | TRUE | 3.01E-02 |
| rs10164099 | 18 | 34690744 | 0.15 | C | T | -0.015 | 0.003 | 1.17E-09 | 0.035 | 0.033 | 2.89E-01 | TRUE | 2.00E-02 |
| rs1017698 | 2 | 219170525 | 0.69 | A | G | -0.011 | 0.002 | 5.13E-10 | -0.035 | 0.026 | 1.75E-01 | TRUE | 2.45E-02 |
| rs10195252 | 2 | 165513091 | 0.36 | C | T | 0.023 | 0.002 | 2.86E-41 | -0.054 | 0.025 | 2.82E-02 | TRUE | 6.89E-08 |
| rs1026462 | 12 | 41821630 | 0.45 | G | A | -0.013 | 0.002 | 1.52E-12 | 0.022 | 0.024 | 3.67E-01 | TRUE | 2.52E-03 |
| rs1035942 | 19 | 7199803 | 0.80 | G | A | 0.012 | 0.002 | 1.50E-09 | 0.029 | 0.029 | 3.23E-01 | TRUE | 1.49E-02 |
| rs10490869 | 3 | 35635145 | 0.78 | T | A | -0.018 | 0.002 | 9.34E-17 | -0.018 | 0.029 | 5.36E-01 | TRUE | 7.17E-05 |
| rs10499013 | 6 | 97946396 | 0.27 | A | G | -0.013 | 0.002 | 5.10E-10 | -0.025 | 0.027 | 3.60E-01 | TRUE | 9.20E-03 |
| rs10504486 | 8 | 71843581 | 0.05 | G | A | 0.018 | 0.003 | 3.06E-08 | 0.052 | 0.052 | 3.21E-01 | TRUE | 3.49E-02 |
| rs10506110 | 12 | 33708552 | 0.39 | C | A | 0.014 | 0.002 | 1.31E-13 | -0.029 | 0.024 | 2.37E-01 | TRUE | 3.43E-03 |
| rs10507524 | 13 | 44684600 | 0.14 | C | T | -0.017 | 0.003 | 1.79E-08 | 0.061 | 0.035 | 7.92E-02 | TRUE | 1.10E-01 |
| rs1056114 | 13 | 54690105 | 0.55 | A | G | -0.010 | 0.002 | 1.30E-08 | -0.031 | 0.024 | 1.98E-01 | TRUE | 4.80E-02 |
| rs10761254 | 9 | 96426344 | 0.61 | C | T | 0.012 | 0.002 | 3.20E-11 | -0.007 | 0.024 | 7.78E-01 | TRUE | 1.02E-03 |
| rs10761785 | 10 | 65318766 | 0.52 | T | G | -0.015 | 0.002 | 6.43E-18 | -0.017 | 0.024 | 4.86E-01 | TRUE | 4.47E-05 |
| rs10795055 | 10 | 3581221 | 0.56 | G | A | 0.011 | 0.002 | 5.43E-09 | 0.047 | 0.024 | 4.74E-02 | TRUE | 1.31E-01 |
| rs10797116 | 9 | 92191467 | 0.51 | C | T | -0.010 | 0.002 | 2.05E-08 | 0.022 | 0.024 | 3.53E-01 | TRUE | 2.46E-02 |
| rs10817158 | 9 | 113928964 | 0.28 | A | C | 0.014 | 0.002 | 4.08E-13 | -0.009 | 0.027 | 7.30E-01 | TRUE | 2.82E-04 |
| rs10827252 | 10 | 33672884 | 0.51 | G | A | -0.011 | 0.002 | 2.03E-10 | -0.010 | 0.024 | 6.88E-01 | TRUE | 2.28E-03 |
| rs10865355 | 2 | 66764997 | 0.58 | G | A | 0.013 | 0.002 | 6.61E-15 | -0.007 | 0.024 | 7.87E-01 | TRUE | 6.42E-05 |
| rs10887759 | 10 | 89603424 | 0.11 | A | G | 0.016 | 0.002 | 2.32E-11 | 0.039 | 0.038 | 3.05E-01 | TRUE | 5.79E-03 |
| rs10919388 | 1 | 170372503 | 0.74 | C | A | -0.027 | 0.002 | 6.40E-43 | 0.038 | 0.027 | 1.59E-01 | TRUE | 1.86E-10 |
| rs10923724 | 1 | 119546842 | 0.68 | T | C | 0.024 | 0.002 | 1.81E-45 | -0.038 | 0.025 | 1.32E-01 | TRUE | 2.07E-10 |
| rs10968576 | 9 | 28414339 | 0.37 | G | A | -0.014 | 0.002 | 2.25E-15 | 0.054 | 0.025 | 2.80E-02 | TRUE | 1.35E-02 |
| rs10991433 | 9 | 107726918 | 0.08 | C | T | -0.025 | 0.003 | 5.73E-20 | -0.013 | 0.044 | 7.69E-01 | TRUE | 3.30E-06 |
| rs11029441 | 11 | 26279145 | 0.07 | C | T | -0.020 | 0.004 | 1.73E-08 | 0.062 | 0.045 | 1.74E-01 | TRUE | 5.40E-02 |
| rs11030107 | 11 | 27694835 | 0.25 | G | A | -0.018 | 0.002 | 4.08E-20 | 0.049 | 0.027 | 7.51E-02 | TRUE | 3.70E-04 |
| rs11048456 | 12 | 26463082 | 0.72 | T | C | -0.027 | 0.002 | 3.00E-43 | -0.044 | 0.026 | 9.32E-02 | TRUE | 7.95E-10 |
| rs11055887 | 12 | 14417179 | 0.19 | A | G | -0.013 | 0.002 | 4.28E-09 | -0.058 | 0.030 | 5.51E-02 | TRUE | 1.32E-01 |
| rs11084399 | 19 | 56000492 | 0.39 | T | C | -0.011 | 0.002 | 1.81E-09 | -0.046 | 0.024 | 6.11E-02 | TRUE | 1.02E-01 |
| rs11133377 | 4 | 56279875 | 0.30 | G | A | 0.014 | 0.002 | 8.92E-13 | -0.021 | 0.026 | 4.21E-01 | TRUE | 1.46E-03 |
| rs11176015 | 12 | 66441684 | 0.26 | T | C | 0.015 | 0.002 | 7.21E-14 | -0.003 | 0.027 | 9.19E-01 | TRUE | 6.57E-05 |
| rs111874795 | 9 | 107901019 | 0.05 | C | T | 0.038 | 0.005 | 7.00E-16 | 0.002 | 0.055 | 9.77E-01 | TRUE | 1.74E-05 |
| rs11187537 | 10 | 95346805 | 0.26 | C | G | 0.013 | 0.002 | 1.73E-10 | 0.004 | 0.027 | 8.80E-01 | TRUE | 7.18E-04 |
| rs11214589 | 11 | 113245048 | 0.47 | A | G | -0.011 | 0.002 | 1.77E-10 | 0.031 | 0.024 | 2.02E-01 | TRUE | 1.90E-02 |
| rs1124639 | 2 | 200775744 | 0.49 | C | T | -0.011 | 0.002 | 7.48E-11 | 0.025 | 0.024 | 2.86E-01 | TRUE | 7.12E-03 |
| rs11247735 | 1 | 26131459 | 0.47 | A | G | 0.011 | 0.002 | 7.14E-09 | 0.012 | 0.024 | 6.16E-01 | TRUE | 7.45E-03 |
| rs113212351 | 1 | 1527952 | 0.35 | C | T | 0.012 | 0.002 | 4.11E-08 | -0.030 | 0.025 | 2.36E-01 | TRUE | 5.15E-02 |
| rs113928896 | 4 | 3140409 | 0.22 | T | C | -0.018 | 0.003 | 1.25E-09 | 0.003 | 0.029 | 9.31E-01 | TRUE | 1.11E-03 |
| rs1142 | 7 | 104756326 | 0.31 | T | C | 0.015 | 0.002 | 2.06E-17 | -0.003 | 0.026 | 8.96E-01 | TRUE | 1.30E-05 |
| rs115092994 | 1 | 46185726 | 0.03 | G | A | 0.028 | 0.005 | 3.62E-08 | -0.197 | 0.074 | 7.23E-03 | TRUE | 4.95E-01 |
| rs115111668 | 6 | 32151882 | 0.00 | T | C | 0.060 | 0.010 | 3.53E-09 | -0.155 | 0.261 | 5.53E-01 | TRUE | 7.20E-03 |
| rs11574218 | 8 | 30937790 | 0.65 | G | T | 0.014 | 0.002 | 4.36E-14 | -0.026 | 0.025 | 2.94E-01 | TRUE | 1.13E-03 |
| rs1158805 | 18 | 40736590 | 0.45 | A | C | -0.013 | 0.002 | 4.95E-13 | 0.019 | 0.024 | 4.36E-01 | TRUE | 8.57E-04 |
| rs11630842 | 15 | 51750652 | 0.56 | A | G | -0.011 | 0.002 | 6.37E-10 | 0.047 | 0.024 | 5.00E-02 | TRUE | 9.29E-02 |
| rs11654395 | 17 | 56040311 | 0.06 | C | T | -0.018 | 0.003 | 3.75E-08 | -0.044 | 0.051 | 3.86E-01 | TRUE | 2.56E-02 |
| rs11664106 | 18 | 2846812 | 0.67 | T | A | 0.020 | 0.002 | 8.69E-22 | 0.049 | 0.026 | 5.34E-02 | TRUE | 4.37E-04 |
| rs11699316 | 20 | 25199992 | 0.12 | A | G | 0.014 | 0.003 | 3.75E-08 | 0.060 | 0.036 | 9.52E-02 | TRUE | 1.18E-01 |
| rs11705729 | 3 | 185507299 | 0.70 | T | A | -0.011 | 0.002 | 1.70E-08 | -0.040 | 0.026 | 1.18E-01 | TRUE | 8.97E-02 |
| rs11711934 | 3 | 52358117 | 0.10 | C | T | 0.018 | 0.002 | 2.64E-15 | -0.004 | 0.039 | 9.12E-01 | TRUE | 2.64E-05 |
| rs11718898 | 3 | 12848822 | 0.62 | C | T | 0.014 | 0.002 | 2.17E-13 | -0.022 | 0.024 | 3.73E-01 | TRUE | 1.67E-03 |
| rs11724804 | 4 | 965779 | 0.46 | A | G | -0.017 | 0.002 | 4.98E-20 | 0.046 | 0.024 | 5.36E-02 | TRUE | 8.84E-04 |
| rs11747001 | 5 | 132412299 | 0.27 | G | A | 0.015 | 0.002 | 8.39E-15 | 0.005 | 0.027 | 8.64E-01 | TRUE | 6.00E-05 |
| rs11782074 | 8 | 142617096 | 0.36 | T | G | 0.011 | 0.002 | 6.32E-09 | 0.037 | 0.025 | 1.31E-01 | TRUE | 6.77E-02 |
| rs11792069 | 9 | 140646121 | 0.18 | G | A | 0.014 | 0.002 | 2.43E-08 | -0.040 | 0.031 | 1.95E-01 | TRUE | 4.89E-02 |
| rs1190982 | 14 | 58815839 | 0.62 | C | T | 0.016 | 0.002 | 5.10E-16 | -0.039 | 0.024 | 1.08E-01 | TRUE | 1.87E-03 |
| rs11956399 | 5 | 112888676 | 0.17 | T | G | 0.016 | 0.002 | 1.26E-12 | 0.056 | 0.032 | 7.63E-02 | TRUE | 1.70E-02 |
| rs11976018 | 7 | 99122437 | 0.22 | A | G | -0.015 | 0.002 | 3.69E-11 | 0.018 | 0.029 | 5.28E-01 | TRUE | 2.58E-03 |
| rs11992444 | 8 | 25464690 | 0.53 | T | G | 0.019 | 0.002 | 4.99E-21 | 0.020 | 0.024 | 4.15E-01 | TRUE | 1.65E-05 |
| rs12024554 | 1 | 19925759 | 0.22 | T | C | -0.015 | 0.002 | 1.08E-10 | -0.056 | 0.029 | 5.00E-02 | TRUE | 6.45E-02 |
| rs12042959 | 1 | 243533273 | 0.16 | G | A | 0.016 | 0.003 | 9.25E-10 | -0.067 | 0.033 | 3.83E-02 | TRUE | 1.09E-01 |
| rs12069549 | 1 | 200036139 | 0.13 | T | C | -0.016 | 0.003 | 3.58E-10 | -0.040 | 0.035 | 2.57E-01 | TRUE | 1.40E-02 |
| rs12101393 | 15 | 92570921 | 0.74 | G | C | 0.014 | 0.002 | 1.19E-10 | 0.043 | 0.027 | 1.11E-01 | TRUE | 3.63E-02 |
| rs12287076 | 11 | 47606865 | 0.77 | C | G | 0.019 | 0.002 | 3.53E-21 | -0.007 | 0.028 | 7.95E-01 | TRUE | 6.90E-07 |
| rs12419064 | 11 | 10320608 | 0.50 | G | A | -0.011 | 0.002 | 5.77E-10 | 0.045 | 0.024 | 5.75E-02 | TRUE | 6.86E-02 |
| rs12430764 | 13 | 93896935 | 0.45 | A | G | 0.011 | 0.002 | 1.87E-09 | 0.022 | 0.024 | 3.69E-01 | TRUE | 1.42E-02 |
| rs1243188 | 10 | 21908803 | 0.29 | C | T | -0.015 | 0.002 | 2.37E-14 | 0.002 | 0.026 | 9.28E-01 | TRUE | 3.63E-05 |
| rs12440605 | 15 | 42102285 | 0.53 | A | G | 0.012 | 0.002 | 4.02E-11 | 0.029 | 0.024 | 2.23E-01 | TRUE | 1.19E-02 |
| rs12459350 | 19 | 2176586 | 0.47 | G | A | 0.013 | 0.002 | 2.23E-15 | 0.021 | 0.024 | 3.70E-01 | TRUE | 4.63E-04 |
| rs12470785 | 2 | 67634003 | 0.66 | A | G | -0.011 | 0.002 | 9.10E-09 | -0.029 | 0.025 | 2.54E-01 | TRUE | 2.83E-02 |
| rs12489576 | 3 | 129658705 | 0.02 | T | C | 0.042 | 0.007 | 6.34E-09 | 0.076 | 0.087 | 3.86E-01 | TRUE | 1.64E-02 |
| rs12495178 | 3 | 85886077 | 0.43 | C | T | 0.012 | 0.002 | 1.32E-12 | -0.020 | 0.024 | 3.97E-01 | TRUE | 2.64E-03 |
| rs1250552 | 10 | 81058027 | 0.56 | G | A | -0.009 | 0.002 | 3.62E-08 | 0.058 | 0.024 | 1.52E-02 | TRUE | 3.46E-01 |
| rs12527712 | 6 | 80916967 | 0.07 | T | C | 0.028 | 0.003 | 3.38E-18 | 0.013 | 0.045 | 7.71E-01 | TRUE | 1.07E-05 |
| rs12575252 | 11 | 8694073 | 0.35 | C | G | -0.015 | 0.002 | 2.32E-18 | -0.014 | 0.025 | 5.77E-01 | TRUE | 3.32E-05 |
| rs12593088 | 15 | 81058640 | 0.28 | A | G | -0.013 | 0.002 | 5.16E-12 | -0.037 | 0.026 | 1.64E-01 | TRUE | 1.36E-02 |
| rs12595496 | 15 | 56528806 | 0.08 | G | A | -0.018 | 0.003 | 2.08E-12 | -0.032 | 0.043 | 4.52E-01 | TRUE | 1.32E-03 |
| rs12602912 | 17 | 65870073 | 0.24 | T | C | 0.018 | 0.002 | 1.23E-16 | 0.057 | 0.028 | 4.13E-02 | TRUE | 4.99E-03 |
| rs12608504 | 19 | 18389135 | 0.67 | G | A | 0.025 | 0.002 | 4.68E-46 | 0.010 | 0.025 | 6.86E-01 | TRUE | 6.55E-13 |
| rs12643960 | 4 | 78655671 | 0.84 | A | G | -0.017 | 0.003 | 1.51E-09 | -0.027 | 0.033 | 4.08E-01 | TRUE | 9.24E-03 |
| rs12669521 | 7 | 77047102 | 0.26 | G | A | 0.013 | 0.002 | 1.29E-09 | 0.009 | 0.027 | 7.50E-01 | TRUE | 2.73E-03 |
| rs12777288 | 10 | 115860058 | 0.21 | C | T | -0.014 | 0.002 | 6.88E-11 | 0.035 | 0.029 | 2.42E-01 | TRUE | 9.62E-03 |
| rs12828016 | 12 | 998365 | 0.29 | T | G | -0.011 | 0.002 | 4.08E-10 | -0.023 | 0.026 | 3.75E-01 | TRUE | 7.52E-03 |
| rs1294410 | 6 | 6738752 | 0.64 | C | T | -0.025 | 0.002 | 5.49E-46 | -0.033 | 0.025 | 1.89E-01 | TRUE | 1.60E-11 |
| rs13063979 | 3 | 131564741 | 0.27 | G | T | -0.014 | 0.002 | 1.19E-12 | 0.057 | 0.027 | 3.41E-02 | TRUE | 4.01E-02 |
| rs13130484 | 4 | 45175691 | 0.47 | T | C | 0.015 | 0.002 | 7.83E-18 | 0.043 | 0.024 | 6.94E-02 | TRUE | 1.69E-03 |
| rs13229637 | 7 | 136717447 | 0.20 | C | T | 0.016 | 0.003 | 1.91E-10 | -0.045 | 0.030 | 1.27E-01 | TRUE | 3.16E-02 |
| rs13232789 | 7 | 84554998 | 0.25 | G | T | -0.011 | 0.002 | 5.09E-09 | 0.036 | 0.028 | 1.97E-01 | TRUE | 4.14E-02 |
| rs13256367 | 8 | 128334900 | 0.31 | C | A | 0.014 | 0.002 | 5.42E-14 | 0.010 | 0.026 | 6.88E-01 | TRUE | 2.44E-04 |
| rs1328757 | 20 | 56135199 | 0.45 | T | C | 0.011 | 0.002 | 1.69E-10 | 0.060 | 0.024 | 1.26E-02 | TRUE | 1.87E-01 |
| rs13316065 | 3 | 49884913 | 0.25 | T | C | 0.017 | 0.002 | 1.34E-20 | 0.029 | 0.027 | 2.91E-01 | TRUE | 2.54E-05 |
| rs1345203 | 2 | 112253851 | 0.18 | C | T | 0.019 | 0.002 | 3.07E-15 | -0.015 | 0.031 | 6.22E-01 | TRUE | 1.49E-04 |
| rs1360485 | 13 | 31031884 | 0.65 | T | C | 0.015 | 0.002 | 2.01E-16 | 0.018 | 0.025 | 4.67E-01 | TRUE | 7.89E-05 |
| rs13642 | 11 | 30432220 | 0.69 | T | A | 0.011 | 0.002 | 9.49E-10 | 0.040 | 0.026 | 1.20E-01 | TRUE | 5.69E-02 |
| rs142989338 | 5 | 56265899 | 0.02 | C | T | -0.037 | 0.007 | 4.30E-08 | -0.107 | 0.078 | 1.71E-01 | TRUE | 7.27E-02 |
| rs1431659 | 8 | 73439070 | 0.76 | G | A | 0.014 | 0.002 | 1.12E-12 | -0.043 | 0.028 | 1.20E-01 | TRUE | 1.07E-02 |
| rs143384 |  |  | 0.44 | G | A | 0.016 | 0.002 | 1.73E-19 | 0.030 | 0.024 | 2.12E-01 | TRUE | 1.08E-04 |
| rs1437 | 10 | 93790523 | 0.46 | G | A | 0.013 | 0.002 | 4.50E-13 | -0.062 | 0.024 | 9.07E-03 | TRUE | 1.00E-01 |
| rs1441264 | 13 | 79580919 | 0.67 | A | G | 0.011 | 0.002 | 3.16E-10 | 0.016 | 0.025 | 5.28E-01 | TRUE | 5.14E-03 |
| rs1443512 | 12 | 54342684 | 0.72 | C | A | 0.028 | 0.002 | 1.06E-45 | -0.039 | 0.026 | 1.45E-01 | TRUE | 1.70E-10 |
| rs1452075 | 3 | 62481063 | 0.71 | T | C | 0.012 | 0.002 | 1.32E-10 | 0.034 | 0.026 | 1.90E-01 | TRUE | 1.67E-02 |
| rs145952040 | 10 | 33461248 | 0.02 | C | T | -0.033 | 0.005 | 2.66E-10 | 0.106 | 0.077 | 1.67E-01 | TRUE | 2.37E-02 |
| rs1464454 | 4 | 157616767 | 0.27 | A | G | -0.010 | 0.002 | 4.15E-08 | -0.060 | 0.027 | 2.35E-02 | TRUE | 3.16E-01 |
| rs147627829 | 6 | 34335088 | 0.02 | A | G | 0.061 | 0.005 | 1.87E-35 | 0.084 | 0.078 | 2.80E-01 | TRUE | 5.37E-09 |
| rs148589464 | 12 | 113579313 | 0.08 | T | C | 0.021 | 0.004 | 4.43E-08 | 0.079 | 0.043 | 6.99E-02 | TRUE | 1.55E-01 |
| rs1488541 | 11 | 103037635 | 0.76 | C | T | -0.013 | 0.002 | 5.02E-09 | 0.042 | 0.028 | 1.34E-01 | TRUE | 6.02E-02 |
| rs15285 | 8 | 19824667 | 0.26 | T | C | -0.012 | 0.002 | 1.62E-09 | -0.033 | 0.027 | 2.26E-01 | TRUE | 2.11E-02 |
| rs1534696 | 7 | 26397239 | 0.56 | A | C | -0.023 | 0.002 | 3.67E-39 | -0.029 | 0.024 | 2.25E-01 | TRUE | 7.48E-10 |
| rs1558902 | 16 | 53803574 | 0.42 | A | T | 0.040 | 0.002 | 3.99E-118 | 0.083 | 0.024 | 5.38E-04 | TRUE | 2.33E-22 |
| rs1563355 | 1 | 219653101 | 0.66 | C | T | -0.028 | 0.002 | 1.59E-49 | 0.044 | 0.025 | 7.92E-02 | TRUE | 1.62E-10 |
| rs1563575 | 2 | 161131694 | 0.79 | A | G | 0.012 | 0.002 | 8.55E-10 | -0.004 | 0.029 | 8.97E-01 | TRUE | 1.44E-03 |
| rs1569135 | 2 | 188115398 | 0.43 | G | A | 0.022 | 0.002 | 2.76E-37 | -0.027 | 0.024 | 2.69E-01 | TRUE | 3.68E-09 |
| rs1609906 | 3 | 94033599 | 0.56 | A | G | -0.013 | 0.002 | 1.22E-10 | -0.002 | 0.024 | 9.22E-01 | TRUE | 9.03E-04 |
| rs1657930 | 15 | 57120989 | 0.85 | A | G | -0.013 | 0.002 | 5.62E-10 | -0.008 | 0.033 | 8.05E-01 | TRUE | 2.06E-03 |
| rs1680490 | 9 | 29642688 | 0.35 | G | A | -0.012 | 0.002 | 2.41E-09 | 0.029 | 0.025 | 2.42E-01 | TRUE | 2.78E-02 |
| rs16853606 | 3 | 107270460 | 0.19 | G | A | -0.013 | 0.002 | 1.22E-08 | -0.040 | 0.030 | 1.87E-01 | TRUE | 4.68E-02 |
| rs17078048 | 6 | 116866019 | 0.19 | G | A | -0.014 | 0.003 | 3.90E-08 | 0.063 | 0.031 | 4.06E-02 | TRUE | 2.25E-01 |
| rs17109256 | 14 | 79939993 | 0.24 | A | G | 0.017 | 0.002 | 2.40E-16 | 0.063 | 0.028 | 2.20E-02 | TRUE | 1.56E-02 |
| rs1718618 | 7 | 68632251 | 0.97 | G | A | -0.024 | 0.004 | 2.19E-10 | 0.005 | 0.070 | 9.46E-01 | TRUE | 8.00E-04 |
| rs17289049 | 3 | 57187036 | 0.12 | G | A | -0.016 | 0.003 | 3.06E-08 | -0.038 | 0.036 | 2.97E-01 | TRUE | 3.81E-02 |
| rs17324331 | 2 | 230739209 | 0.31 | C | G | 0.012 | 0.002 | 1.79E-10 | -0.012 | 0.026 | 6.46E-01 | TRUE | 2.50E-03 |
| rs17326656 | 2 | 48962291 | 0.16 | T | G | 0.015 | 0.002 | 4.17E-13 | -0.023 | 0.032 | 4.70E-01 | TRUE | 8.84E-04 |
| rs1757471 | 10 | 34168090 | 0.57 | T | C | 0.013 | 0.002 | 5.89E-13 | 0.002 | 0.024 | 9.25E-01 | TRUE | 1.69E-04 |
| rs17644283 | 4 | 26308792 | 0.64 | G | A | 0.015 | 0.002 | 3.24E-17 | 0.030 | 0.025 | 2.31E-01 | TRUE | 6.00E-04 |
| rs17724992 | 19 | 18454825 | 0.22 | G | A | 0.014 | 0.002 | 1.29E-13 | 0.014 | 0.028 | 6.31E-01 | TRUE | 2.53E-04 |
| rs17764730 | 5 | 127357526 | 0.16 | T | C | -0.011 | 0.002 | 4.86E-08 | -0.037 | 0.033 | 2.63E-01 | TRUE | 4.68E-02 |
| rs17782313 | 18 | 57851097 | 0.18 | C | T | -0.026 | 0.002 | 3.13E-38 | 0.103 | 0.030 | 5.38E-04 | TRUE | 2.83E-05 |
| rs1789882 | 4 | 100235053 | 0.81 | G | A | 0.016 | 0.002 | 9.97E-12 | -0.013 | 0.031 | 6.74E-01 | TRUE | 6.69E-04 |
| rs1800437 | 19 | 46181392 | 0.26 | C | G | -0.021 | 0.002 | 6.23E-22 | 0.015 | 0.027 | 5.86E-01 | TRUE | 2.99E-06 |
| rs1815892 | 2 | 9616728 | 0.49 | T | G | 0.011 | 0.002 | 3.90E-09 | 0.002 | 0.024 | 9.33E-01 | TRUE | 2.08E-03 |
| rs1863652 | 4 | 95991417 | 0.30 | A | G | -0.010 | 0.002 | 3.05E-08 | -0.031 | 0.026 | 2.36E-01 | TRUE | 5.36E-02 |
| rs1891216 | 1 | 7728391 | 0.42 | G | T | -0.010 | 0.002 | 2.03E-08 | 0.035 | 0.024 | 1.48E-01 | TRUE | 5.70E-02 |
| rs1896686 | 5 | 66384463 | 0.46 | A | G | -0.010 | 0.002 | 3.12E-08 | -0.005 | 0.024 | 8.40E-01 | TRUE | 5.12E-03 |
| rs1942831 | 18 | 63444000 | 0.38 | C | G | 0.011 | 0.002 | 2.93E-08 | -0.004 | 0.025 | 8.58E-01 | TRUE | 3.51E-03 |
| rs1969354 | 12 | 123741776 | 0.79 | C | T | -0.017 | 0.002 | 2.52E-15 | 0.023 | 0.029 | 4.24E-01 | TRUE | 3.88E-04 |
| rs1992145 | 15 | 74723644 | 0.56 | A | G | 0.011 | 0.002 | 5.77E-10 | -0.009 | 0.024 | 7.18E-01 | TRUE | 2.48E-03 |
| rs2008514 | 16 | 28825605 | 0.41 | A | G | 0.017 | 0.002 | 8.56E-23 | 0.019 | 0.024 | 4.41E-01 | TRUE | 2.03E-06 |
| rs2022464 | 6 | 108945370 | 0.63 | C | A | -0.013 | 0.002 | 1.67E-11 | 0.043 | 0.025 | 7.96E-02 | TRUE | 2.85E-02 |
| rs2061007 | 15 | 100269680 | 0.64 | C | G | -0.011 | 0.002 | 4.65E-09 | -0.015 | 0.025 | 5.55E-01 | TRUE | 7.98E-03 |
| rs2069443 | 7 | 150755173 | 0.29 | G | T | -0.012 | 0.002 | 1.40E-08 | 0.076 | 0.026 | 3.15E-03 | TRUE | 5.66E-01 |
| rs2112347 | 5 | 75015242 | 0.42 | G | T | 0.015 | 0.002 | 8.41E-18 | -0.065 | 0.024 | 7.24E-03 | TRUE | 1.23E-02 |
| rs2161097 | 5 | 103945178 | 0.39 | T | C | 0.013 | 0.002 | 2.11E-12 | 0.037 | 0.024 | 1.32E-01 | TRUE | 1.20E-02 |
| rs2161228 | 5 | 88001798 | 0.09 | T | C | 0.019 | 0.003 | 4.32E-11 | 0.079 | 0.042 | 5.84E-02 | TRUE | 4.75E-02 |
| rs2167750 | 4 | 89730074 | 0.52 | T | C | 0.020 | 0.002 | 6.47E-29 | 0.048 | 0.024 | 4.55E-02 | TRUE | 1.82E-05 |
| rs2176598 | 11 | 43864278 | 0.73 | C | T | 0.013 | 0.002 | 5.51E-11 | -0.013 | 0.027 | 6.21E-01 | TRUE | 1.28E-03 |
| rs2178663 | 12 | 124433905 | 0.30 | T | C | -0.026 | 0.002 | 2.79E-43 | -0.040 | 0.026 | 1.23E-01 | TRUE | 6.53E-10 |
| rs220381 | 16 | 3344618 | 0.71 | A | G | -0.011 | 0.002 | 2.79E-08 | -0.031 | 0.026 | 2.38E-01 | TRUE | 4.34E-02 |
| rs2235148 | 22 | 35664941 | 0.61 | C | T | 0.012 | 0.002 | 9.23E-11 | 0.049 | 0.024 | 4.29E-02 | TRUE | 6.21E-02 |
| rs2236519 | 20 | 45529571 | 0.37 | A | G | 0.021 | 0.002 | 1.89E-30 | 0.010 | 0.025 | 6.72E-01 | TRUE | 1.94E-09 |
| rs2254069 | 10 | 122875589 | 0.15 | A | G | 0.021 | 0.003 | 4.77E-15 | -0.025 | 0.033 | 4.38E-01 | TRUE | 4.21E-04 |
| rs2276390 | 11 | 111895254 | 0.42 | T | G | -0.021 | 0.002 | 5.76E-28 | -0.021 | 0.024 | 3.89E-01 | TRUE | 1.58E-07 |
| rs2294239 | 22 | 29449477 | 0.40 | G | A | 0.020 | 0.002 | 3.17E-31 | 0.031 | 0.024 | 2.03E-01 | TRUE | 1.29E-07 |
| rs2298632 | 1 | 23710475 | 0.55 | T | C | -0.014 | 0.002 | 3.49E-17 | 0.012 | 0.024 | 6.31E-01 | TRUE | 3.06E-05 |
| rs2333496 | 4 | 177609609 | 0.73 | T | C | 0.010 | 0.002 | 1.08E-08 | -0.012 | 0.027 | 6.56E-01 | TRUE | 6.05E-03 |
| rs2371767 | 3 | 64718258 | 0.22 | C | G | -0.029 | 0.002 | 1.00E-54 | -0.062 | 0.029 | 3.08E-02 | TRUE | 1.10E-10 |
| rs2391168 | 7 | 25862790 | 0.27 | A | C | 0.026 | 0.002 | 1.38E-34 | 0.036 | 0.027 | 1.89E-01 | TRUE | 2.42E-08 |
| rs2398893 | 9 | 96758342 | 0.31 | G | A | 0.016 | 0.002 | 9.01E-17 | 0.002 | 0.026 | 9.34E-01 | TRUE | 1.13E-05 |
| rs244722 | 5 | 176534724 | 0.48 | C | A | -0.013 | 0.002 | 5.08E-11 | -0.008 | 0.024 | 7.43E-01 | TRUE | 1.22E-03 |
| rs2481665 | 1 | 62594677 | 0.37 | C | T | 0.011 | 0.002 | 3.78E-11 | -0.038 | 0.025 | 1.21E-01 | TRUE | 2.43E-02 |
| rs2509963 | 11 | 62192931 | 0.74 | C | T | -0.014 | 0.002 | 2.81E-12 | 0.011 | 0.027 | 6.76E-01 | TRUE | 4.50E-04 |
| rs2526886 | 14 | 71359064 | 0.69 | T | G | 0.013 | 0.002 | 3.65E-09 | 0.021 | 0.026 | 4.04E-01 | TRUE | 1.20E-02 |
| rs2696309 | 10 | 36227101 | 0.67 | C | T | -0.011 | 0.002 | 4.11E-08 | 0.035 | 0.025 | 1.68E-01 | TRUE | 7.90E-02 |
| rs2715135 | 7 | 50750128 | 0.65 | G | T | 0.011 | 0.002 | 4.09E-09 | -0.027 | 0.025 | 2.74E-01 | TRUE | 1.88E-02 |
| rs2727331 | 17 | 61926990 | 0.42 | T | G | 0.013 | 0.002 | 1.35E-12 | 0.021 | 0.024 | 3.83E-01 | TRUE | 2.32E-03 |
| rs2791558 | 1 | 219583791 | 0.21 | T | C | 0.013 | 0.002 | 4.06E-10 | -0.032 | 0.029 | 2.67E-01 | TRUE | 1.17E-02 |
| rs2799465 | 9 | 126544609 | 0.16 | C | T | -0.015 | 0.003 | 3.07E-09 | 0.009 | 0.033 | 7.78E-01 | TRUE | 3.31E-03 |
| rs2802536 | 1 | 98556115 | 0.77 | C | T | 0.015 | 0.002 | 7.79E-11 | -0.014 | 0.028 | 6.06E-01 | TRUE | 1.86E-03 |
| rs2808104 | 10 | 32112728 | 0.15 | T | C | -0.012 | 0.002 | 2.29E-09 | -0.022 | 0.033 | 5.01E-01 | TRUE | 8.74E-03 |
| rs2815749 | 1 | 72814783 | 0.86 | G | A | -0.017 | 0.002 | 6.37E-15 | 0.010 | 0.034 | 7.80E-01 | TRUE | 5.51E-05 |
| rs2823096 | 21 | 16522082 | 0.19 | G | A | 0.013 | 0.002 | 3.17E-08 | 0.055 | 0.030 | 7.15E-02 | TRUE | 1.37E-01 |
| rs2836179 | 21 | 39544159 | 0.55 | A | G | -0.013 | 0.002 | 2.44E-13 | -0.016 | 0.024 | 5.04E-01 | TRUE | 6.82E-04 |
| rs2838006 | 21 | 42653567 | 0.62 | C | T | -0.011 | 0.002 | 4.97E-09 | -0.021 | 0.024 | 3.97E-01 | TRUE | 1.71E-02 |
| rs28451064 | 21 | 35593827 | 0.15 | A | G | 0.018 | 0.003 | 4.23E-09 | -0.014 | 0.033 | 6.61E-01 | TRUE | 4.86E-03 |
| rs28647893 | 9 | 136994893 | 0.42 | T | C | -0.012 | 0.002 | 2.91E-09 | 0.020 | 0.024 | 4.16E-01 | TRUE | 1.44E-02 |
| rs2925979 | 16 | 81534790 | 0.68 | C | T | 0.022 | 0.002 | 1.62E-31 | -0.004 | 0.025 | 8.70E-01 | TRUE | 2.77E-10 |
| rs2964006 | 5 | 153213880 | 0.56 | G | T | 0.013 | 0.002 | 3.57E-11 | 0.008 | 0.024 | 7.48E-01 | TRUE | 1.20E-03 |
| rs2981423 | 18 | 76741423 | 0.06 | C | T | 0.019 | 0.004 | 3.64E-08 | 0.041 | 0.049 | 4.01E-01 | TRUE | 2.22E-02 |
| rs2993481 | 1 | 2973433 | 0.89 | T | A | -0.015 | 0.002 | 1.32E-10 | 0.031 | 0.039 | 4.26E-01 | TRUE | 6.26E-03 |
| rs3092781 |  |  | 0.50 | T | C | -0.015 | 0.002 | 1.92E-17 | -0.006 | 0.024 | 7.89E-01 | TRUE | 1.19E-05 |
| rs317646 | 12 | 69657145 | 0.65 | A | G | -0.011 | 0.002 | 2.56E-08 | -0.011 | 0.025 | 6.71E-01 | TRUE | 9.00E-03 |
| rs332105 | 2 | 119444229 | 0.50 | A | G | -0.014 | 0.002 | 1.12E-14 | -0.003 | 0.024 | 9.15E-01 | TRUE | 4.52E-05 |
| rs34848977 | 22 | 38165587 | 0.34 | A | G | 0.012 | 0.002 | 2.85E-08 | 0.003 | 0.025 | 8.91E-01 | TRUE | 3.23E-03 |
| rs35154326 | 16 | 24862414 | 0.31 | G | A | 0.014 | 0.002 | 4.85E-10 | 0.015 | 0.026 | 5.68E-01 | TRUE | 3.66E-03 |
| rs35169799 | 11 | 64031241 | 0.09 | T | C | 0.037 | 0.004 | 6.26E-20 | 0.037 | 0.041 | 3.64E-01 | TRUE | 3.36E-05 |
| rs35643724 | 12 | 54387102 | 0.05 | A | G | -0.025 | 0.003 | 2.88E-19 | 0.017 | 0.057 | 7.60E-01 | TRUE | 4.18E-06 |
| rs35708461 | 8 | 103543446 | 0.23 | T | C | 0.014 | 0.002 | 1.73E-08 | 0.039 | 0.028 | 1.62E-01 | TRUE | 6.29E-02 |
| rs3747579 | 16 | 4445327 | 0.71 | T | C | -0.015 | 0.002 | 4.07E-16 | -0.001 | 0.026 | 9.54E-01 | TRUE | 8.53E-06 |
| rs3754963 | 2 | 166185707 | 0.70 | T | A | 0.011 | 0.002 | 2.13E-08 | -0.020 | 0.026 | 4.37E-01 | TRUE | 1.65E-02 |
| rs3764002 | 12 | 108618630 | 0.34 | T | C | -0.021 | 0.002 | 3.91E-27 | -0.004 | 0.025 | 8.73E-01 | TRUE | 2.67E-08 |
| rs377436 | 6 | 109527104 | 0.36 | A | G | -0.011 | 0.002 | 1.78E-08 | 0.000 | 0.025 | 9.88E-01 | TRUE | 2.04E-03 |
| rs3786897 | 19 | 33893008 | 0.41 | G | A | -0.024 | 0.002 | 1.45E-43 | -0.047 | 0.024 | 5.32E-02 | TRUE | 3.64E-09 |
| rs3789615 | 1 | 114941326 | 0.47 | T | C | -0.013 | 0.002 | 5.81E-15 | 0.004 | 0.024 | 8.79E-01 | TRUE | 4.20E-05 |
| rs3804381 | 4 | 102143616 | 0.78 | G | A | 0.012 | 0.002 | 5.00E-10 | 0.021 | 0.028 | 4.69E-01 | TRUE | 6.19E-03 |
| rs3825061 | 11 | 118944675 | 0.29 | T | C | 0.014 | 0.002 | 5.45E-15 | 0.032 | 0.026 | 2.24E-01 | TRUE | 1.21E-03 |
| rs3891424 | 2 | 239365456 | 0.06 | A | G | -0.028 | 0.004 | 4.00E-10 | -0.013 | 0.049 | 7.87E-01 | TRUE | 1.45E-03 |
| rs39312 | 7 | 116954785 | 0.41 | C | A | -0.015 | 0.002 | 3.50E-18 | 0.018 | 0.024 | 4.63E-01 | TRUE | 6.28E-05 |
| rs4075353 | 10 | 102415609 | 0.35 | A | G | -0.012 | 0.002 | 9.32E-09 | -0.028 | 0.025 | 2.60E-01 | TRUE | 3.47E-02 |
| rs414865 | 4 | 124097632 | 0.82 | A | T | 0.011 | 0.002 | 1.19E-08 | -0.008 | 0.031 | 8.05E-01 | TRUE | 3.86E-03 |
| rs4239275 | 17 | 79923718 | 0.63 | C | T | 0.012 | 0.002 | 3.26E-11 | 0.014 | 0.025 | 5.80E-01 | TRUE | 2.12E-03 |
| rs4243130 | 16 | 76838461 | 0.16 | G | T | -0.012 | 0.002 | 1.68E-08 | 0.039 | 0.033 | 2.30E-01 | TRUE | 3.56E-02 |
| rs4283409 | 2 | 228977142 | 0.59 | G | A | -0.010 | 0.002 | 2.34E-08 | 0.012 | 0.024 | 6.29E-01 | TRUE | 7.99E-03 |
| rs4372913 | 2 | 114517748 | 0.29 | G | A | -0.014 | 0.002 | 8.60E-11 | 0.012 | 0.026 | 6.35E-01 | TRUE | 2.40E-03 |
| rs4382592 | 9 | 134870755 | 0.73 | G | T | 0.012 | 0.002 | 2.71E-10 | -0.042 | 0.027 | 1.13E-01 | TRUE | 3.78E-02 |
| rs4395620 | 5 | 106328326 | 0.52 | C | T | 0.011 | 0.002 | 3.57E-09 | -0.032 | 0.024 | 1.84E-01 | TRUE | 3.94E-02 |
| rs4454042 | 5 | 155824774 | 0.69 | C | T | 0.013 | 0.002 | 1.37E-10 | -0.018 | 0.026 | 4.93E-01 | TRUE | 4.34E-03 |
| rs4586926 | 4 | 15375527 | 0.41 | C | A | 0.011 | 0.002 | 1.00E-08 | -0.008 | 0.024 | 7.53E-01 | TRUE | 3.56E-03 |
| rs459193 | 5 | 55806751 | 0.68 | G | A | 0.024 | 0.002 | 1.07E-35 | -0.011 | 0.025 | 6.58E-01 | TRUE | 1.37E-10 |
| rs4635727 | 3 | 18705035 | 0.67 | A | G | 0.012 | 0.002 | 2.35E-09 | 0.049 | 0.025 | 5.14E-02 | TRUE | 1.22E-01 |
| rs4673616 | 2 | 212281381 | 0.27 | G | T | -0.012 | 0.002 | 2.70E-09 | 0.037 | 0.027 | 1.69E-01 | TRUE | 3.49E-02 |
| rs4714704 | 6 | 43825023 | 0.33 | A | G | -0.017 | 0.002 | 1.13E-16 | 0.093 | 0.025 | 2.02E-04 | TRUE | 1.68E-01 |
| rs4718966 | 7 | 70040558 | 0.41 | T | C | 0.010 | 0.002 | 1.69E-08 | 0.032 | 0.024 | 1.85E-01 | TRUE | 5.18E-02 |
| rs4738141 | 8 | 72469742 | 0.24 | G | A | -0.020 | 0.002 | 7.34E-25 | 0.043 | 0.028 | 1.26E-01 | TRUE | 1.11E-05 |
| rs4773395 | 13 | 112241549 | 0.50 | G | A | 0.011 | 0.002 | 3.42E-09 | 0.021 | 0.024 | 3.86E-01 | TRUE | 1.43E-02 |
| rs4779526 | 15 | 31705683 | 0.72 | T | A | 0.013 | 0.002 | 2.09E-10 | 0.032 | 0.027 | 2.39E-01 | TRUE | 1.63E-02 |
| rs4788204 | 16 | 29995218 | 0.40 | A | G | 0.017 | 0.002 | 2.64E-21 | 0.011 | 0.024 | 6.47E-01 | TRUE | 2.81E-06 |
| rs4837261 | 9 | 119142351 | 0.80 | C | T | 0.013 | 0.002 | 1.45E-08 | -0.023 | 0.029 | 4.33E-01 | TRUE | 1.79E-02 |
| rs4851283 | 2 | 100894802 | 0.22 | G | C | 0.016 | 0.002 | 1.54E-14 | 0.047 | 0.029 | 1.06E-01 | TRUE | 4.31E-03 |
| rs4868256 | 5 | 172748679 | 0.60 | T | C | 0.011 | 0.002 | 8.66E-09 | 0.016 | 0.024 | 5.20E-01 | TRUE | 1.15E-02 |
| rs4894803 | 3 | 171800256 | 0.42 | G | A | 0.015 | 0.002 | 3.74E-16 | 0.027 | 0.024 | 2.74E-01 | TRUE | 3.87E-04 |
| rs4902632 | 14 | 69149428 | 0.25 | T | A | 0.016 | 0.002 | 1.78E-10 | 0.034 | 0.028 | 2.25E-01 | TRUE | 1.37E-02 |
| rs525731 | 3 | 37571827 | 0.57 | A | G | 0.013 | 0.002 | 7.84E-13 | -0.022 | 0.024 | 3.52E-01 | TRUE | 2.02E-03 |
| rs536665 | 11 | 85322400 | 0.09 | G | A | -0.017 | 0.002 | 2.18E-12 | -0.024 | 0.042 | 5.75E-01 | TRUE | 7.39E-04 |
| rs55677077 | 12 | 50219508 | 0.54 | C | T | -0.010 | 0.002 | 2.77E-08 | 0.040 | 0.024 | 9.56E-02 | TRUE | 9.76E-02 |
| rs55747707 | 7 | 73037366 | 0.18 | A | G | -0.015 | 0.002 | 3.40E-11 | 0.039 | 0.031 | 2.02E-01 | TRUE | 1.10E-02 |
| rs55920843 | 2 | 158412701 | 0.00 | G | T | 0.062 | 0.009 | 1.55E-11 | -0.525 | 0.288 | 6.81E-02 | TRUE | 3.39E-02 |
| rs579682 | 11 | 122014110 | 0.79 | T | C | -0.013 | 0.002 | 2.02E-11 | 0.039 | 0.029 | 1.79E-01 | TRUE | 1.39E-02 |
| rs587271 | 1 | 54743111 | 0.68 | T | C | 0.011 | 0.002 | 3.18E-08 | 0.013 | 0.026 | 6.28E-01 | TRUE | 9.20E-03 |
| rs591939 | 17 | 40698075 | 0.21 | G | A | -0.018 | 0.002 | 9.11E-15 | 0.029 | 0.029 | 3.11E-01 | TRUE | 9.56E-04 |
| rs605066 | 6 | 139829666 | 0.52 | T | C | -0.019 | 0.002 | 2.38E-26 | -0.036 | 0.024 | 1.34E-01 | TRUE | 6.85E-06 |
| rs60570301 | 19 | 18597196 | 0.16 | A | G | -0.017 | 0.003 | 1.48E-08 | 0.020 | 0.032 | 5.28E-01 | TRUE | 1.24E-02 |
| rs6130360 | 20 | 42010996 | 0.10 | G | A | 0.015 | 0.003 | 1.13E-09 | 0.024 | 0.040 | 5.45E-01 | TRUE | 5.92E-03 |
| rs61876729 | 11 | 778857 | 0.11 | G | A | 0.021 | 0.003 | 5.55E-10 | -0.046 | 0.039 | 2.36E-01 | TRUE | 1.63E-02 |
| rs61986159 | 14 | 53956168 | 0.23 | T | C | 0.014 | 0.002 | 4.85E-09 | 0.001 | 0.028 | 9.73E-01 | TRUE | 1.55E-03 |
| rs62063286 | 17 | 44041987 | 0.19 | C | T | 0.024 | 0.002 | 2.49E-23 | 0.007 | 0.030 | 8.27E-01 | TRUE | 1.43E-07 |
| rs62095889 | 18 | 21069068 | 0.36 | A | G | -0.017 | 0.002 | 1.62E-15 | -0.028 | 0.025 | 2.52E-01 | TRUE | 9.53E-04 |
| rs62206548 | 20 | 51708340 | 0.17 | T | G | -0.017 | 0.003 | 5.05E-11 | -0.030 | 0.032 | 3.48E-01 | TRUE | 5.75E-03 |
| rs62424543 | 6 | 133190249 | 0.10 | G | A | -0.022 | 0.004 | 2.22E-08 | 0.034 | 0.039 | 3.89E-01 | TRUE | 1.98E-02 |
| rs62506196 | 8 | 60264465 | 0.23 | C | A | -0.016 | 0.003 | 2.70E-09 | 0.080 | 0.028 | 3.79E-03 | TRUE | 4.58E-01 |
| rs6430168 | 2 | 148121393 | 0.72 | G | T | 0.012 | 0.002 | 1.38E-08 | -0.067 | 0.026 | 1.15E-02 | TRUE | 3.73E-01 |
| rs6433219 | 2 | 171421125 | 0.29 | A | G | 0.014 | 0.002 | 2.25E-11 | 0.022 | 0.026 | 3.94E-01 | TRUE | 3.14E-03 |
| rs645040 | 3 | 135926622 | 0.85 | T | G | 0.016 | 0.002 | 1.15E-14 | 0.017 | 0.033 | 6.08E-01 | TRUE | 1.57E-04 |
| rs647248 | 11 | 89917699 | 0.54 | A | G | 0.011 | 0.002 | 6.49E-09 | 0.021 | 0.024 | 3.79E-01 | TRUE | 1.60E-02 |
| rs6474945 | 9 | 15670492 | 0.51 | T | G | -0.010 | 0.002 | 2.67E-09 | -0.017 | 0.024 | 4.62E-01 | TRUE | 9.63E-03 |
| rs6590683 | 11 | 133110361 | 0.48 | C | T | 0.011 | 0.002 | 3.29E-10 | -0.021 | 0.024 | 3.68E-01 | TRUE | 8.50E-03 |
| rs6688233 | 1 | 9335745 | 0.18 | T | C | 0.019 | 0.002 | 3.02E-19 | 0.026 | 0.031 | 4.00E-01 | TRUE | 4.63E-05 |
| rs668871 | 6 | 160769811 | 0.50 | T | C | -0.013 | 0.002 | 7.19E-12 | 0.003 | 0.024 | 9.08E-01 | TRUE | 4.31E-04 |
| rs67184556 | 11 | 36327794 | 0.37 | T | C | -0.012 | 0.002 | 4.93E-08 | 0.008 | 0.025 | 7.56E-01 | TRUE | 6.03E-03 |
| rs6719428 | 2 | 66238005 | 0.32 | C | T | -0.017 | 0.002 | 3.76E-18 | -0.005 | 0.025 | 8.48E-01 | TRUE | 9.48E-06 |
| rs6743060 | 2 | 629510 | 0.84 | A | C | 0.027 | 0.002 | 1.80E-32 | 0.033 | 0.032 | 2.95E-01 | TRUE | 1.63E-08 |
| rs6749646 | 2 | 25193998 | 0.86 | T | A | -0.021 | 0.002 | 5.99E-22 | -0.053 | 0.034 | 1.25E-01 | TRUE | 1.13E-04 |
| rs6785245 | 3 | 82647990 | 0.35 | C | T | -0.011 | 0.002 | 7.21E-10 | -0.009 | 0.025 | 7.12E-01 | TRUE | 2.30E-03 |
| rs6795831 | 3 | 129341403 | 0.17 | C | A | 0.029 | 0.002 | 2.09E-37 | -0.008 | 0.032 | 7.93E-01 | TRUE | 6.14E-11 |
| rs6861681 | 5 | 173362458 | 0.41 | A | G | 0.018 | 0.002 | 1.38E-22 | 0.011 | 0.024 | 6.39E-01 | TRUE | 5.62E-07 |
| rs6870983 | 5 | 87697533 | 0.19 | T | C | -0.016 | 0.002 | 2.08E-14 | -0.003 | 0.030 | 9.12E-01 | TRUE | 4.29E-05 |
| rs6874848 | 5 | 118688115 | 0.67 | A | T | -0.014 | 0.002 | 1.75E-12 | 0.004 | 0.025 | 8.90E-01 | TRUE | 3.08E-04 |
| rs6898870 | 5 | 53293107 | 0.25 | A | G | -0.016 | 0.002 | 1.03E-14 | 0.053 | 0.027 | 5.26E-02 | TRUE | 9.88E-03 |
| rs6932767 | 6 | 14595873 | 0.77 | T | G | 0.016 | 0.002 | 2.20E-12 | 0.004 | 0.028 | 8.90E-01 | TRUE | 2.27E-04 |
| rs693301 | 18 | 13097207 | 0.43 | T | C | 0.011 | 0.002 | 4.02E-09 | 0.055 | 0.024 | 2.16E-02 | TRUE | 2.24E-01 |
| rs6941962 | 6 | 100629725 | 0.14 | A | T | 0.024 | 0.003 | 5.28E-20 | 0.021 | 0.035 | 5.40E-01 | TRUE | 1.05E-05 |
| rs7070670 | 10 | 61842645 | 0.28 | T | C | -0.013 | 0.002 | 3.64E-10 | -0.048 | 0.027 | 6.77E-02 | TRUE | 6.34E-02 |
| rs7101 | 14 | 75745626 | 0.80 | T | C | -0.011 | 0.002 | 1.97E-08 | -0.042 | 0.030 | 1.62E-01 | TRUE | 6.89E-02 |
| rs711869 | 2 | 13073967 | 0.61 | A | G | -0.017 | 0.002 | 2.38E-21 | -0.009 | 0.024 | 7.18E-01 | TRUE | 1.40E-06 |
| rs71385734 | 16 | 2160503 | 0.18 | G | T | 0.019 | 0.003 | 1.06E-13 | 0.006 | 0.031 | 8.55E-01 | TRUE | 1.26E-04 |
| rs7143963 | 14 | 103304425 | 0.22 | T | C | 0.014 | 0.002 | 3.31E-10 | 0.065 | 0.029 | 2.33E-02 | TRUE | 1.53E-01 |
| rs7206608 | 16 | 82872628 | 0.65 | G | C | -0.013 | 0.002 | 2.48E-11 | -0.054 | 0.025 | 2.97E-02 | TRUE | 7.53E-02 |
| rs7213608 | 17 | 21279289 | 0.64 | T | C | -0.017 | 0.002 | 6.95E-19 | -0.087 | 0.025 | 3.97E-04 | TRUE | 6.04E-02 |
| rs7217226 | 17 | 2136065 | 0.35 | G | T | -0.014 | 0.002 | 9.94E-15 | -0.008 | 0.025 | 7.61E-01 | TRUE | 1.16E-04 |
| rs7222 | 12 | 2055266 | 0.50 | C | T | 0.011 | 0.002 | 1.17E-09 | -0.008 | 0.024 | 7.45E-01 | TRUE | 2.73E-03 |
| rs7250389 | 19 | 30325332 | 0.36 | T | G | 0.013 | 0.002 | 1.33E-11 | -0.043 | 0.025 | 8.56E-02 | TRUE | 2.46E-02 |
| rs72940579 | 11 | 66182450 | 0.29 | T | C | -0.012 | 0.002 | 3.53E-09 | 0.026 | 0.026 | 3.31E-01 | TRUE | 1.78E-02 |
| rs72959041 | 6 | 127454893 | 0.06 | A | G | 0.126 | 0.004 | 4.56E-183 | 0.032 | 0.049 | 5.16E-01 | TRUE | 2.73E-50 |
| rs7311622 | 12 | 98772975 | 0.33 | T | C | -0.012 | 0.002 | 2.96E-11 | -0.004 | 0.025 | 8.72E-01 | TRUE | 5.96E-04 |
| rs7350648 | 13 | 81064756 | 0.81 | T | G | 0.014 | 0.003 | 1.99E-08 | 0.021 | 0.031 | 4.96E-01 | TRUE | 1.61E-02 |
| rs7395513 | 11 | 69262756 | 0.45 | A | G | -0.017 | 0.002 | 2.46E-18 | 0.024 | 0.024 | 3.15E-01 | TRUE | 1.31E-04 |
| rs7453812 | 6 | 153405199 | 0.96 | G | T | -0.024 | 0.004 | 3.33E-09 | 0.030 | 0.063 | 6.33E-01 | TRUE | 5.16E-03 |
| rs74628422 | 12 | 116034706 | 0.26 | A | G | 0.014 | 0.002 | 9.10E-09 | -0.015 | 0.027 | 5.86E-01 | TRUE | 6.66E-03 |
| rs747249 | 11 | 130271647 | 0.66 | G | A | 0.011 | 0.002 | 2.65E-09 | -0.038 | 0.025 | 1.27E-01 | TRUE | 6.13E-02 |
| rs747601 | 11 | 13274553 | 0.28 | T | A | -0.013 | 0.002 | 7.27E-11 | 0.036 | 0.026 | 1.71E-01 | TRUE | 2.12E-02 |
| rs7492628 | 14 | 91547136 | 0.72 | G | C | -0.013 | 0.002 | 7.89E-11 | -0.015 | 0.027 | 5.78E-01 | TRUE | 2.05E-03 |
| rs7528123 | 1 | 176751613 | 0.31 | C | T | -0.010 | 0.002 | 1.42E-08 | -0.029 | 0.026 | 2.55E-01 | TRUE | 3.31E-02 |
| rs7531656 | 1 | 49828663 | 0.62 | G | A | 0.016 | 0.002 | 1.41E-19 | -0.010 | 0.025 | 6.96E-01 | TRUE | 5.09E-06 |
| rs753804 | 9 | 95498420 | 0.19 | C | G | 0.017 | 0.002 | 2.06E-13 | 0.047 | 0.030 | 1.23E-01 | TRUE | 6.13E-03 |
| rs7591387 | 2 | 43756032 | 0.06 | T | C | 0.018 | 0.003 | 9.30E-11 | -0.119 | 0.052 | 2.15E-02 | TRUE | 1.29E-01 |
| rs7598832 | 2 | 67853013 | 0.40 | T | C | -0.019 | 0.002 | 1.22E-26 | 0.023 | 0.024 | 3.36E-01 | TRUE | 1.17E-06 |
| rs7599312 | 2 | 213413231 | 0.30 | A | G | -0.011 | 0.002 | 2.36E-09 | 0.035 | 0.026 | 1.77E-01 | TRUE | 3.54E-02 |
| rs7631052 | 3 | 81866235 | 0.42 | A | C | 0.011 | 0.002 | 2.45E-08 | -0.016 | 0.024 | 5.03E-01 | TRUE | 1.55E-02 |
| rs7647305 | 3 | 185834290 | 0.82 | C | T | -0.014 | 0.002 | 8.28E-12 | 0.067 | 0.031 | 2.83E-02 | TRUE | 7.11E-02 |
| rs7680787 | 4 | 122624065 | 0.33 | C | T | 0.010 | 0.002 | 4.40E-08 | 0.003 | 0.025 | 8.91E-01 | TRUE | 5.20E-03 |
| rs77165542 | 2 | 430975 | 0.02 | T | C | -0.042 | 0.006 | 1.61E-14 | -0.068 | 0.092 | 4.60E-01 | TRUE | 4.80E-04 |
| rs7744833 | 6 | 20581828 | 0.26 | G | A | 0.013 | 0.002 | 6.88E-11 | -0.030 | 0.027 | 2.62E-01 | TRUE | 9.10E-03 |
| rs7754297 | 6 | 43365207 | 0.80 | G | A | 0.014 | 0.002 | 3.38E-09 | -0.031 | 0.029 | 2.90E-01 | TRUE | 2.13E-02 |
| rs780159 | 10 | 80907147 | 0.57 | G | A | -0.013 | 0.002 | 2.86E-14 | 0.020 | 0.024 | 4.00E-01 | TRUE | 4.39E-04 |
| rs7861458 | 9 | 11771159 | 0.82 | A | C | 0.013 | 0.002 | 3.55E-08 | 0.011 | 0.031 | 7.29E-01 | TRUE | 6.66E-03 |
| rs789351 | 4 | 145868370 | 0.37 | T | C | 0.012 | 0.002 | 7.57E-13 | 0.039 | 0.025 | 1.13E-01 | TRUE | 1.01E-02 |
| rs7898903 | 10 | 77469230 | 0.08 | T | C | -0.015 | 0.003 | 3.68E-08 | -0.038 | 0.043 | 3.79E-01 | TRUE | 2.65E-02 |
| rs7907173 | 10 | 5648787 | 0.46 | A | G | -0.011 | 0.002 | 9.43E-10 | 0.045 | 0.024 | 6.17E-02 | TRUE | 8.97E-02 |
| rs7919055 | 10 | 63808475 | 0.06 | C | T | -0.028 | 0.005 | 4.81E-10 | -0.023 | 0.051 | 6.53E-01 | TRUE | 2.65E-03 |
| rs793456 | 3 | 99525631 | 0.58 | A | G | -0.011 | 0.002 | 3.56E-09 | -0.041 | 0.024 | 8.43E-02 | TRUE | 8.42E-02 |
| rs797486 | 13 | 51221618 | 0.90 | A | C | 0.032 | 0.003 | 1.09E-34 | 0.089 | 0.039 | 2.32E-02 | TRUE | 3.29E-06 |
| rs7982447 | 13 | 54453811 | 0.19 | C | T | -0.013 | 0.002 | 6.07E-09 | 0.036 | 0.030 | 2.35E-01 | TRUE | 3.21E-02 |
| rs8054299 | 16 | 53498655 | 0.73 | G | C | 0.011 | 0.002 | 2.46E-08 | 0.016 | 0.027 | 5.44E-01 | TRUE | 1.14E-02 |
| rs805770 | 20 | 5668714 | 0.41 | T | C | 0.018 | 0.002 | 3.42E-22 | 0.028 | 0.024 | 2.47E-01 | TRUE | 1.27E-05 |
| rs8070737 | 17 | 3981066 | 0.19 | T | G | 0.016 | 0.002 | 7.39E-12 | 0.019 | 0.030 | 5.37E-01 | TRUE | 1.21E-03 |
| rs8071778 | 17 | 46080233 | 0.14 | C | G | -0.016 | 0.003 | 6.62E-11 | -0.047 | 0.034 | 1.69E-01 | TRUE | 2.16E-02 |
| rs809955 | 4 | 140874760 | 0.36 | A | G | -0.016 | 0.002 | 2.48E-14 | -0.027 | 0.025 | 2.86E-01 | TRUE | 1.01E-03 |
| rs8141715 | 22 | 47214749 | 0.34 | G | T | -0.014 | 0.002 | 1.45E-11 | 0.032 | 0.025 | 2.04E-01 | TRUE | 9.25E-03 |
| rs852425 | 7 | 5566671 | 0.39 | G | A | -0.010 | 0.002 | 8.40E-09 | 0.003 | 0.024 | 9.12E-01 | TRUE | 3.01E-03 |
| rs860262 | 7 | 28194397 | 0.49 | A | C | -0.012 | 0.002 | 1.54E-09 | -0.020 | 0.024 | 3.97E-01 | TRUE | 1.34E-02 |
| rs863750 | 12 | 124505444 | 0.58 | T | C | 0.026 | 0.002 | 6.20E-51 | 0.045 | 0.024 | 6.01E-02 | TRUE | 3.06E-11 |
| rs876383 | 15 | 74333413 | 0.38 | A | G | -0.013 | 0.002 | 2.13E-13 | -0.010 | 0.025 | 6.77E-01 | TRUE | 3.08E-04 |
| rs881301 | 8 | 38332318 | 0.43 | C | T | -0.013 | 0.002 | 1.18E-13 | 0.020 | 0.024 | 4.00E-01 | TRUE | 9.29E-04 |
| rs889398 | 16 | 69556715 | 0.43 | T | C | -0.017 | 0.002 | 1.99E-23 | 0.005 | 0.024 | 8.35E-01 | TRUE | 1.47E-07 |
| rs901630 | 6 | 98539519 | 0.34 | T | C | -0.012 | 0.002 | 6.81E-12 | 0.006 | 0.025 | 8.12E-01 | TRUE | 3.45E-04 |
| rs905938 | 1 | 154991389 | 0.23 | C | T | 0.013 | 0.002 | 7.83E-11 | 0.014 | 0.028 | 6.09E-01 | TRUE | 1.63E-03 |
| rs924814 | 3 | 15649550 | 0.35 | A | G | 0.011 | 0.002 | 6.05E-11 | 0.067 | 0.025 | 6.88E-03 | TRUE | 1.78E-01 |
| rs9425301 | 1 | 172363815 | 0.60 | C | A | 0.018 | 0.002 | 2.41E-25 | 0.015 | 0.024 | 5.36E-01 | TRUE | 7.87E-07 |
| rs9515201 | 13 | 111040798 | 0.73 | C | A | -0.012 | 0.002 | 6.63E-11 | 0.010 | 0.027 | 7.19E-01 | TRUE | 8.79E-04 |
| rs9556979 | 13 | 99241507 | 0.35 | G | T | -0.011 | 0.002 | 6.63E-09 | 0.003 | 0.025 | 9.06E-01 | TRUE | 1.86E-03 |
| rs9644033 | 8 | 23610639 | 0.79 | T | A | 0.019 | 0.002 | 2.10E-19 | 0.054 | 0.029 | 6.43E-02 | TRUE | 1.05E-03 |
| rs979012 | 20 | 6623374 | 0.70 | C | T | 0.012 | 0.002 | 7.86E-11 | -0.029 | 0.026 | 2.62E-01 | TRUE | 1.12E-02 |
| rs9872031 | 3 | 12496461 | 0.46 | A | G | 0.020 | 0.002 | 3.57E-29 | 0.076 | 0.024 | 1.43E-03 | TRUE | 7.38E-04 |
| rs987237 | 6 | 50803050 | 0.21 | G | A | -0.020 | 0.002 | 9.98E-20 | 0.036 | 0.029 | 2.17E-01 | TRUE | 1.09E-04 |
| rs9951872 | 18 | 46678832 | 0.79 | G | A | 0.021 | 0.002 | 1.24E-17 | 0.013 | 0.029 | 6.53E-01 | TRUE | 2.08E-05 |
| rs9969455 | 8 | 12617155 | 0.38 | G | A | 0.011 | 0.002 | 7.10E-10 | -0.004 | 0.025 | 8.80E-01 | TRUE | 1.36E-03 |
| rs998584 | 6 | 43757896 | 0.48 | A | C | 0.035 | 0.002 | 7.42E-92 | 0.055 | 0.024 | 2.04E-02 | TRUE | 3.74E-20 |
| rs998732 | 19 | 19378671 | 0.10 | G | A | 0.017 | 0.002 | 1.04E-12 | -0.012 | 0.039 | 7.51E-01 | TRUE | 3.11E-04 |
| rs998749 | 3 | 168972802 | 0.51 | G | A | 0.013 | 0.002 | 2.16E-14 | 0.006 | 0.024 | 8.01E-01 | TRUE | 8.98E-05 |
| **adult WHR_adj_BMI** | | | | | | | | | | | | | |
| rs10049088 | 3 | 156797648 | 0.38 | T | C | -0.029 | 0.002 | 1.45E-59 | 0.013 | 0.026 | 6.13E-01 | TRUE | 1.17E-16 |
| rs10074193 | 5 | 34041686 | 0.15 | T | C | -0.015 | 0.002 | 2.33E-09 | -0.030 | 0.032 | 3.61E-01 | TRUE | 1.17E-02 |
| rs10172295 | 2 | 58201202 | 0.41 | A | G | 0.012 | 0.002 | 4.51E-11 | 0.001 | 0.024 | 9.71E-01 | TRUE | 3.51E-04 |
| rs10195252 | 2 | 165513091 | 0.58 | C | T | 0.032 | 0.002 | 8.43E-78 | -0.054 | 0.025 | 2.82E-02 | TRUE | 2.68E-17 |
| rs10264590 | 7 | 77357748 | 0.48 | G | A | 0.018 | 0.002 | 8.32E-23 | 0.013 | 0.024 | 5.75E-01 | TRUE | 1.70E-06 |
| rs1029645 | 17 | 70295760 | 0.55 | A | G | -0.013 | 0.002 | 7.20E-13 | -0.005 | 0.024 | 8.45E-01 | TRUE | 2.06E-04 |
| rs1035942 | 19 | 7199803 | 0.27 | G | A | 0.014 | 0.002 | 2.14E-12 | 0.029 | 0.029 | 3.23E-01 | TRUE | 2.87E-03 |
| rs1042917 | 21 | 47545768 | 0.48 | A | G | -0.013 | 0.002 | 2.56E-12 | -0.022 | 0.024 | 3.61E-01 | TRUE | 2.55E-03 |
| rs1045241 | 5 | 118729286 | 0.28 | T | C | -0.019 | 0.002 | 3.51E-22 | 0.000 | 0.026 | 9.93E-01 | TRUE | 1.46E-07 |
| rs10462028 | 4 | 56298300 | 0.32 | A | G | 0.019 | 0.002 | 2.95E-23 | 0.014 | 0.025 | 5.82E-01 | TRUE | 8.81E-07 |
| rs10463416 | 5 | 148572915 | 0.36 | A | G | 0.014 | 0.002 | 9.28E-14 | -0.014 | 0.025 | 5.68E-01 | TRUE | 2.77E-04 |
| rs10498948 | 6 | 85405761 | 0.10 | T | G | 0.021 | 0.003 | 4.08E-12 | -0.066 | 0.042 | 1.18E-01 | TRUE | 1.74E-02 |
| rs10502148 | 11 | 111647998 | 0.36 | T | C | -0.023 | 0.002 | 5.87E-36 | -0.010 | 0.024 | 6.71E-01 | TRUE | 5.64E-11 |
| rs10507524 | 13 | 44684600 | 0.90 | C | T | -0.018 | 0.003 | 2.68E-09 | 0.061 | 0.035 | 7.92E-02 | TRUE | 8.94E-02 |
| rs1053593 | 22 | 35660875 | 0.64 | T | G | -0.014 | 0.002 | 3.07E-15 | 0.056 | 0.024 | 2.04E-02 | TRUE | 2.41E-02 |
| rs1057119 | 14 | 23746269 | 0.77 | G | C | 0.013 | 0.002 | 9.17E-10 | 0.066 | 0.028 | 1.98E-02 | TRUE | 1.82E-01 |
| rs10803236 | 1 | 236191170 | 0.53 | G | A | -0.010 | 0.002 | 2.94E-08 | 0.066 | 0.024 | 6.73E-03 | TRUE | 5.14E-01 |
| rs10820747 | 9 | 107686823 | 0.24 | A | G | 0.027 | 0.002 | 1.10E-31 | 0.061 | 0.030 | 4.50E-02 | TRUE | 2.79E-06 |
| rs10850127 | 12 | 113512945 | 0.31 | T | C | 0.013 | 0.002 | 8.17E-11 | 0.003 | 0.032 | 9.21E-01 | TRUE | 5.99E-04 |
| rs10878367 | 12 | 66436097 | 0.30 | A | T | 0.019 | 0.002 | 5.84E-21 | -0.012 | 0.027 | 6.52E-01 | TRUE | 1.95E-06 |
| rs10880823 | 12 | 45986738 | 0.77 | C | T | -0.013 | 0.002 | 5.65E-10 | 0.062 | 0.030 | 3.48E-02 | TRUE | 1.04E-01 |
| rs10887759 | 10 | 89603424 | 0.16 | A | G | 0.017 | 0.002 | 1.06E-12 | 0.039 | 0.038 | 3.05E-01 | TRUE | 2.40E-03 |
| rs10891490 | 11 | 112885527 | 0.42 | C | T | 0.010 | 0.002 | 8.94E-09 | -0.027 | 0.025 | 2.70E-01 | TRUE | 2.43E-02 |
| rs10919388 | 1 | 170372503 | 0.27 | C | A | -0.033 | 0.002 | 9.71E-66 | 0.038 | 0.027 | 1.59E-01 | TRUE | 1.12E-16 |
| rs10923724 | 1 | 119546842 | 0.56 | T | C | 0.035 | 0.002 | 1.25E-89 | -0.038 | 0.025 | 1.32E-01 | TRUE | 1.49E-22 |
| rs10963067 | 9 | 17367946 | 0.09 | A | C | -0.021 | 0.003 | 5.86E-11 | -0.037 | 0.052 | 4.79E-01 | TRUE | 3.31E-03 |
| rs10992414 | 9 | 95448381 | 0.83 | C | A | -0.022 | 0.002 | 1.10E-21 | 0.047 | 0.030 | 1.21E-01 | TRUE | 8.21E-05 |
| rs11048456 | 12 | 26463082 | 0.76 | T | C | -0.034 | 0.002 | 1.05E-69 | -0.044 | 0.026 | 9.32E-02 | TRUE | 8.69E-17 |
| rs1105881 | 15 | 42072530 | 0.63 | G | C | -0.016 | 0.002 | 1.10E-16 | -0.041 | 0.024 | 9.37E-02 | TRUE | 2.44E-03 |
| rs11134029 | 5 | 4025324 | 0.55 | C | T | 0.014 | 0.002 | 5.72E-13 | 0.023 | 0.024 | 3.34E-01 | TRUE | 2.26E-03 |
| rs11187537 | 10 | 95346805 | 0.26 | C | G | 0.016 | 0.002 | 2.43E-15 | 0.004 | 0.027 | 8.80E-01 | TRUE | 4.69E-05 |
| rs11205773 | 1 | 51284905 | 0.91 | C | T | 0.019 | 0.003 | 2.31E-09 | 0.044 | 0.051 | 3.85E-01 | TRUE | 1.14E-02 |
| rs114847269 | 18 | 15301379 | 0.98 | G | A | -0.041 | 0.007 | 2.54E-08 | -0.305 | 0.167 | 6.76E-02 | TRUE | 1.45E-01 |
| rs11592754 | 10 | 32619572 | 0.86 | C | A | -0.022 | 0.003 | 3.22E-18 | -0.063 | 0.033 | 5.76E-02 | TRUE | 2.26E-03 |
| rs11596221 | 10 | 30374786 | 0.06 | A | G | -0.022 | 0.004 | 2.16E-08 | 0.002 | 0.057 | 9.72E-01 | TRUE | 2.89E-03 |
| rs11649660 | 16 | 85251413 | 0.79 | G | C | 0.015 | 0.002 | 2.49E-09 | 0.007 | 0.030 | 8.16E-01 | TRUE | 2.13E-03 |
| rs11664106 | 18 | 2846812 | 0.63 | T | A | 0.028 | 0.002 | 5.90E-41 | 0.049 | 0.026 | 5.34E-02 | TRUE | 1.73E-08 |
| rs11679731 | 2 | 43589064 | 0.82 | T | A | -0.014 | 0.002 | 6.38E-11 | -0.018 | 0.031 | 5.61E-01 | TRUE | 2.47E-03 |
| rs116825877 | 4 | 145277105 | 0.97 | G | A | -0.043 | 0.006 | 3.43E-11 | -0.027 | 0.056 | 6.27E-01 | TRUE | 1.38E-03 |
| rs1169644 | 16 | 86537032 | 0.77 | A | G | 0.012 | 0.002 | 3.90E-08 | -0.056 | 0.030 | 5.69E-02 | TRUE | 1.70E-01 |
| rs11717954 | 3 | 136112109 | 0.60 | G | A | -0.010 | 0.002 | 1.27E-08 | -0.058 | 0.024 | 1.47E-02 | TRUE | 2.97E-01 |
| rs11718898 | 3 | 12848822 | 0.32 | C | T | 0.016 | 0.002 | 8.87E-17 | -0.022 | 0.024 | 3.73E-01 | TRUE | 2.00E-04 |
| rs11721258 | 3 | 64605119 | 0.72 | G | A | -0.012 | 0.002 | 6.27E-10 | -0.007 | 0.025 | 7.93E-01 | TRUE | 1.70E-03 |
| rs11724804 | 4 | 965779 | 0.44 | A | G | -0.017 | 0.002 | 7.61E-21 | 0.046 | 0.024 | 5.36E-02 | TRUE | 4.94E-04 |
| rs11747001 | 5 | 132412299 | 0.76 | G | A | 0.017 | 0.002 | 7.73E-18 | 0.005 | 0.027 | 8.64E-01 | TRUE | 6.60E-06 |
| rs11757455 | 6 | 112522852 | 0.06 | A | G | -0.022 | 0.004 | 2.20E-09 | 0.042 | 0.059 | 4.73E-01 | TRUE | 8.62E-03 |
| rs117630421 | 7 | 27140237 | 0.05 | A | G | -0.028 | 0.005 | 8.29E-10 | -0.096 | 0.077 | 2.14E-01 | TRUE | 2.54E-02 |
| rs11770285 | 7 | 107616011 | 0.90 | G | C | 0.031 | 0.003 | 7.06E-24 | -0.038 | 0.047 | 4.15E-01 | TRUE | 2.55E-06 |
| rs11893688 | 2 | 9695282 | 0.66 | T | C | 0.014 | 0.002 | 7.75E-13 | 0.011 | 0.024 | 6.38E-01 | TRUE | 4.12E-04 |
| rs11899550 | 2 | 158451825 | 0.07 | T | C | 0.020 | 0.004 | 1.22E-08 | 0.060 | 0.051 | 2.42E-01 | TRUE | 3.93E-02 |
| rs1190982 | 14 | 58815839 | 0.30 | C | T | 0.016 | 0.002 | 2.46E-16 | -0.039 | 0.024 | 1.08E-01 | TRUE | 1.50E-03 |
| rs11929253 | 3 | 150074251 | 0.18 | A | G | 0.015 | 0.002 | 3.86E-11 | 0.003 | 0.034 | 9.22E-01 | TRUE | 4.14E-04 |
| rs11992444 | 8 | 25464690 | 0.51 | T | G | 0.023 | 0.002 | 5.57E-32 | 0.020 | 0.024 | 4.15E-01 | TRUE | 1.97E-08 |
| rs12141791 | 1 | 156419786 | 0.70 | A | G | -0.012 | 0.002 | 3.61E-08 | 0.017 | 0.025 | 4.88E-01 | TRUE | 1.41E-02 |
| rs12144610 | 1 | 119501103 | 0.83 | T | A | -0.021 | 0.002 | 4.00E-22 | 0.029 | 0.027 | 2.82E-01 | TRUE | 1.33E-05 |
| rs12293855 | 11 | 69184067 | 0.13 | A | G | 0.021 | 0.003 | 1.10E-12 | 0.022 | 0.038 | 5.57E-01 | TRUE | 6.67E-04 |
| rs12325187 | 16 | 3364997 | 0.73 | G | C | 0.013 | 0.002 | 8.30E-10 | 0.041 | 0.026 | 1.14E-01 | TRUE | 5.22E-02 |
| rs12415793 | 10 | 93696575 | 0.37 | A | G | -0.011 | 0.002 | 1.24E-08 | -0.064 | 0.024 | 7.00E-03 | TRUE | 4.54E-01 |
| rs12435790 | 14 | 35154381 | 0.92 | G | A | 0.021 | 0.004 | 1.16E-09 | -0.010 | 0.046 | 8.28E-01 | TRUE | 1.78E-03 |
| rs12441543 | 15 | 31689543 | 0.29 | A | G | -0.019 | 0.002 | 8.68E-22 | -0.025 | 0.026 | 3.39E-01 | TRUE | 1.66E-05 |
| rs12454712 | 18 | 60845884 | 0.62 | C | T | 0.017 | 0.002 | 1.33E-20 | -0.037 | 0.024 | 1.25E-01 | TRUE | 1.35E-04 |
| rs12459350 | 19 | 2176586 | 0.54 | G | A | 0.014 | 0.002 | 2.39E-16 | 0.021 | 0.024 | 3.70E-01 | TRUE | 1.91E-04 |
| rs1250259 | 2 | 216300482 | 0.74 | A | T | -0.016 | 0.002 | 1.04E-15 | -0.057 | 0.029 | 4.90E-02 | TRUE | 5.46E-03 |
| rs12527712 | 6 | 80916967 | 0.09 | T | C | 0.034 | 0.003 | 6.85E-25 | 0.013 | 0.045 | 7.71E-01 | TRUE | 1.09E-07 |
| rs12595496 | 15 | 56528806 | 0.87 | G | A | -0.026 | 0.003 | 1.05E-23 | -0.032 | 0.043 | 4.52E-01 | TRUE | 2.45E-06 |
| rs12608504 | 19 | 18389135 | 0.36 | G | A | 0.026 | 0.002 | 3.09E-47 | 0.010 | 0.025 | 6.86E-01 | TRUE | 1.26E-13 |
| rs12684047 | 9 | 111972671 | 0.19 | A | T | -0.015 | 0.002 | 6.85E-11 | -0.002 | 0.031 | 9.59E-01 | TRUE | 6.10E-04 |
| rs12709891 | 19 | 46185217 | 0.21 | A | C | 0.013 | 0.002 | 7.44E-09 | 0.073 | 0.027 | 7.38E-03 | TRUE | 3.99E-01 |
| rs12774134 | 10 | 4963327 | 0.12 | T | C | -0.019 | 0.003 | 1.99E-12 | 0.036 | 0.035 | 3.07E-01 | TRUE | 3.67E-03 |
| rs12823266 | 12 | 131441717 | 0.72 | G | A | 0.013 | 0.002 | 2.37E-10 | -0.017 | 0.025 | 5.06E-01 | TRUE | 4.89E-03 |
| rs12828318 | 12 | 133766122 | 0.83 | G | A | 0.017 | 0.002 | 3.84E-14 | -0.019 | 0.033 | 5.69E-01 | TRUE | 3.32E-04 |
| rs1286769 | 3 | 25585166 | 0.46 | A | G | -0.010 | 0.002 | 1.58E-08 | 0.015 | 0.024 | 5.26E-01 | TRUE | 9.77E-03 |
| rs12936587 | 17 | 17543722 | 0.48 | A | G | -0.018 | 0.002 | 9.25E-27 | -0.007 | 0.025 | 7.69E-01 | TRUE | 2.07E-08 |
| rs1294410 | 6 | 6738752 | 0.38 | C | T | -0.031 | 0.002 | 2.26E-69 | -0.033 | 0.025 | 1.89E-01 | TRUE | 2.91E-16 |
| rs12999687 | 2 | 25512438 | 0.52 | G | T | 0.015 | 0.002 | 3.07E-17 | 0.029 | 0.024 | 2.19E-01 | TRUE | 3.06E-04 |
| rs13107325 | 4 | 103188709 | 0.08 | T | C | -0.031 | 0.003 | 3.69E-19 | 0.075 | 0.102 | 4.62E-01 | TRUE | 2.16E-05 |
| rs13223034 | 7 | 42701627 | 0.32 | T | C | -0.013 | 0.002 | 3.67E-12 | -0.039 | 0.024 | 1.10E-01 | TRUE | 1.46E-02 |
| rs13256367 | 8 | 128334900 | 0.65 | C | A | 0.018 | 0.002 | 6.15E-22 | 0.010 | 0.026 | 6.88E-01 | TRUE | 1.28E-06 |
| rs13379794 | 15 | 98367100 | 0.39 | A | G | 0.011 | 0.002 | 4.34E-09 | 0.047 | 0.024 | 5.16E-02 | TRUE | 1.01E-01 |
| rs13406302 | 2 | 37874850 | 0.73 | C | A | -0.014 | 0.002 | 2.17E-12 | -0.030 | 0.028 | 2.78E-01 | TRUE | 3.38E-03 |
| rs1345203 | 2 | 112253851 | 0.78 | C | T | 0.026 | 0.002 | 2.23E-28 | -0.015 | 0.031 | 6.22E-01 | TRUE | 3.13E-08 |
| rs1360485 | 13 | 31031884 | 0.68 | T | C | 0.011 | 0.002 | 8.04E-10 | 0.018 | 0.025 | 4.67E-01 | TRUE | 8.45E-03 |
| rs1364422 | 7 | 130445981 | 0.27 | T | C | 0.016 | 0.002 | 1.07E-16 | 0.022 | 0.026 | 4.13E-01 | TRUE | 1.57E-04 |
| rs1415361 | 1 | 103562263 | 0.59 | T | C | 0.016 | 0.002 | 5.83E-16 | 0.021 | 0.024 | 3.83E-01 | TRUE | 2.95E-04 |
| rs1425486 | 4 | 157683685 | 0.35 | T | C | -0.012 | 0.002 | 2.91E-11 | -0.067 | 0.028 | 1.47E-02 | TRUE | 1.28E-01 |
| rs143384 | NA | NA | 0.59 | G | A | 0.018 | 0.002 | 9.86E-25 | 0.030 | 0.024 | 2.12E-01 | TRUE | 3.98E-06 |
| rs1440372 | 15 | 67033151 | 0.27 | C | T | -0.016 | 0.002 | 9.53E-16 | -0.017 | 0.027 | 5.35E-01 | TRUE | 1.00E-04 |
| rs1443512 | 12 | 54342684 | 0.22 | C | A | 0.031 | 0.002 | 1.16E-53 | -0.039 | 0.026 | 1.45E-01 | TRUE | 1.23E-12 |
| rs144926207 | 9 | 140079779 | 0.96 | C | T | 0.032 | 0.005 | 1.60E-09 | 0.039 | 0.069 | 5.74E-01 | TRUE | 5.41E-03 |
| rs146095395 | 2 | 239769885 | 0.18 | T | C | -0.020 | 0.003 | 2.60E-12 | -0.068 | 0.030 | 2.31E-02 | TRUE | 6.90E-02 |
| rs146167147 | 3 | 129170212 | 0.01 | T | C | 0.060 | 0.009 | 7.20E-11 | 0.028 | 0.096 | 7.70E-01 | TRUE | 1.09E-03 |
| rs1473553 | 12 | 122985057 | 0.75 | T | G | -0.012 | 0.002 | 1.57E-08 | -0.047 | 0.030 | 1.14E-01 | TRUE | 9.75E-02 |
| rs147627829 | 6 | 34335088 | 0.04 | A | G | 0.086 | 0.005 | 1.27E-66 | 0.084 | 0.078 | 2.80E-01 | TRUE | 7.49E-17 |
| rs1494204 | 10 | 27904321 | 0.42 | T | C | -0.015 | 0.002 | 8.06E-16 | -0.003 | 0.025 | 8.90E-01 | TRUE | 1.70E-05 |
| rs149492184 | 6 | 32020717 | 0.01 | T | G | 0.065 | 0.010 | 2.97E-10 | -0.119 | 0.268 | 6.57E-01 | TRUE | 2.37E-03 |
| rs150493080 | 13 | 50609483 | 0.05 | T | G | 0.026 | 0.005 | 6.38E-09 | 0.003 | 0.049 | 9.45E-01 | TRUE | 1.84E-03 |
| rs151235402 | 20 | 569164 | 0.02 | T | C | 0.052 | 0.008 | 4.70E-10 | -0.016 | 0.096 | 8.67E-01 | TRUE | 1.31E-03 |
| rs1529884 | 15 | 47896440 | 0.34 | G | A | 0.011 | 0.002 | 2.26E-08 | 0.004 | 0.026 | 8.87E-01 | TRUE | 3.76E-03 |
| rs1534696 | 7 | 26397239 | 0.57 | A | C | -0.024 | 0.002 | 3.15E-44 | -0.029 | 0.024 | 2.25E-01 | TRUE | 1.58E-11 |
| rs1547149 | 13 | 22476288 | 0.65 | G | A | -0.012 | 0.002 | 6.55E-11 | 0.012 | 0.025 | 6.42E-01 | TRUE | 1.87E-03 |
| rs1569135 | 2 | 188115398 | 0.54 | G | A | 0.021 | 0.002 | 2.99E-33 | -0.027 | 0.024 | 2.69E-01 | TRUE | 2.25E-08 |
| rs1577099 | 13 | 93894245 | 0.52 | T | C | 0.010 | 0.002 | 3.13E-08 | 0.021 | 0.024 | 3.81E-01 | TRUE | 2.52E-02 |
| rs16853606 | 3 | 107270460 | 0.84 | G | A | -0.015 | 0.002 | 1.25E-10 | -0.040 | 0.030 | 1.87E-01 | TRUE | 1.47E-02 |
| rs16957415 | 16 | 67418957 | 0.96 | G | A | 0.031 | 0.004 | 3.29E-13 | 0.063 | 0.061 | 3.02E-01 | TRUE | 2.15E-03 |
| rs17067999 | 13 | 78163787 | 0.14 | T | C | -0.016 | 0.003 | 1.45E-10 | -0.008 | 0.041 | 8.39E-01 | TRUE | 1.20E-03 |
| rs17154889 | 5 | 102305065 | 0.31 | A | C | -0.016 | 0.002 | 3.90E-16 | -0.004 | 0.025 | 8.78E-01 | TRUE | 1.25E-05 |
| rs1727332 | 12 | 123718301 | 0.74 | T | C | 0.023 | 0.002 | 2.66E-24 | 0.038 | 0.028 | 1.69E-01 | TRUE | 1.09E-05 |
| rs17311057 | 3 | 78841789 | 0.29 | T | C | 0.015 | 0.002 | 1.72E-12 | 0.029 | 0.027 | 2.72E-01 | TRUE | 2.73E-03 |
| rs17325066 | 3 | 129116419 | 0.91 | C | T | -0.023 | 0.004 | 2.38E-11 | -0.030 | 0.038 | 4.31E-01 | TRUE | 3.30E-03 |
| rs17326656 | 2 | 48962291 | 0.23 | T | G | 0.015 | 0.002 | 5.77E-13 | -0.023 | 0.032 | 4.70E-01 | TRUE | 8.63E-04 |
| rs17357115 | 7 | 120872121 | 0.54 | A | C | 0.014 | 0.002 | 3.30E-14 | 0.035 | 0.024 | 1.51E-01 | TRUE | 3.99E-03 |
| rs174829 | 3 | 37535577 | 0.35 | G | A | -0.014 | 0.002 | 6.28E-13 | -0.051 | 0.024 | 3.36E-02 | TRUE | 2.80E-02 |
| rs17703883 | 15 | 51530097 | 0.76 | C | T | -0.015 | 0.002 | 7.27E-13 | -0.015 | 0.027 | 5.71E-01 | TRUE | 7.77E-04 |
| rs17819328 | 3 | 12489342 | 0.57 | G | T | -0.025 | 0.002 | 4.79E-46 | 0.078 | 0.024 | 9.94E-04 | TRUE | 3.62E-07 |
| rs1792672 | 18 | 45392531 | 0.55 | T | C | -0.011 | 0.002 | 1.87E-08 | -0.001 | 0.024 | 9.64E-01 | TRUE | 2.78E-03 |
| rs1892203 | 20 | 51696096 | 0.49 | T | C | -0.020 | 0.002 | 1.48E-23 | -0.029 | 0.024 | 2.31E-01 | TRUE | 1.22E-05 |
| rs1922437 | 12 | 107058045 | 0.52 | T | C | 0.012 | 0.002 | 5.97E-10 | 0.036 | 0.024 | 1.35E-01 | TRUE | 4.13E-02 |
| rs1979527 | 3 | 127400781 | 0.20 | A | C | 0.013 | 0.002 | 2.36E-09 | 0.017 | 0.027 | 5.13E-01 | TRUE | 6.07E-03 |
| rs2023772 | 7 | 93131162 | 0.51 | C | A | 0.012 | 0.002 | 1.42E-11 | 0.031 | 0.024 | 1.91E-01 | TRUE | 1.05E-02 |
| rs2041733 | 16 | 11229589 | 0.44 | C | T | -0.010 | 0.002 | 1.86E-08 | 0.033 | 0.024 | 1.71E-01 | TRUE | 5.31E-02 |
| rs2047937 | 16 | 49864791 | 0.52 | T | C | -0.015 | 0.002 | 7.31E-18 | -0.012 | 0.024 | 6.29E-01 | TRUE | 1.96E-05 |
| rs2058914 | 15 | 63831984 | 0.73 | A | G | -0.012 | 0.002 | 1.56E-10 | -0.009 | 0.024 | 7.22E-01 | TRUE | 2.22E-03 |
| rs2145272 | 20 | 6626218 | 0.64 | A | G | -0.025 | 0.002 | 3.95E-43 | -0.030 | 0.026 | 2.43E-01 | TRUE | 1.20E-10 |
| rs2167750 | 4 | 89730074 | 0.47 | T | C | 0.027 | 0.002 | 5.53E-53 | 0.048 | 0.024 | 4.55E-02 | TRUE | 5.77E-11 |
| rs2186955 | 11 | 74375475 | 0.81 | C | T | 0.012 | 0.002 | 1.53E-08 | -0.046 | 0.037 | 2.17E-01 | TRUE | 4.72E-02 |
| rs2200155 | 12 | 33734935 | 0.37 | A | G | -0.014 | 0.002 | 1.26E-13 | -0.021 | 0.024 | 3.85E-01 | TRUE | 1.39E-03 |
| rs2227831 | 5 | 76023494 | 0.95 | G | A | -0.026 | 0.004 | 1.65E-09 | 0.064 | 0.061 | 2.88E-01 | TRUE | 1.87E-02 |
| rs2236519 | 20 | 45529571 | 0.37 | A | G | 0.030 | 0.002 | 4.07E-58 | 0.010 | 0.025 | 6.72E-01 | TRUE | 4.46E-16 |
| rs2254708 | 9 | 107667147 | 0.84 | C | T | 0.021 | 0.002 | 4.42E-19 | -0.038 | 0.037 | 3.12E-01 | TRUE | 4.38E-05 |
| rs2270445 | 17 | 8219478 | 0.51 | G | A | -0.011 | 0.002 | 1.48E-09 | 0.039 | 0.024 | 1.03E-01 | TRUE | 5.69E-02 |
| rs227756 | 17 | 54752709 | 0.26 | A | G | 0.012 | 0.002 | 8.99E-09 | -0.025 | 0.025 | 3.03E-01 | TRUE | 2.32E-02 |
| rs2294239 | 22 | 29449477 | 0.57 | G | A | 0.024 | 0.002 | 4.04E-44 | 0.031 | 0.024 | 2.03E-01 | TRUE | 2.77E-11 |
| rs2298632 | 1 | 23710475 | 0.49 | T | C | -0.016 | 0.002 | 4.96E-19 | 0.012 | 0.024 | 6.31E-01 | TRUE | 6.01E-06 |
| rs2301254 | 11 | 32457675 | 0.62 | G | A | 0.013 | 0.002 | 5.35E-11 | 0.022 | 0.026 | 4.02E-01 | TRUE | 3.84E-03 |
| rs2306363 | 11 | 65405600 | 0.20 | T | G | -0.017 | 0.002 | 1.61E-12 | -0.091 | 0.033 | 5.50E-03 | TRUE | 1.18E-01 |
| rs2333496 | 4 | 177609609 | 0.69 | T | C | 0.011 | 0.002 | 7.15E-10 | -0.012 | 0.027 | 6.56E-01 | TRUE | 2.29E-03 |
| rs2343813 | 5 | 149878840 | 0.08 | T | C | 0.019 | 0.003 | 8.62E-10 | -0.015 | 0.034 | 6.56E-01 | TRUE | 2.93E-03 |
| rs2357996 | 2 | 11380529 | 0.13 | C | A | 0.016 | 0.003 | 7.27E-09 | 0.114 | 0.055 | 3.76E-02 | TRUE | 1.86E-01 |
| rs2371767 | 3 | 64718258 | 0.27 | C | G | -0.040 | 0.002 | 1.00E-100 | -0.062 | 0.029 | 3.08E-02 | TRUE | 6.29E-22 |
| rs2376585 | 17 | 76417883 | 0.20 | C | T | -0.018 | 0.002 | 5.50E-14 | -0.016 | 0.035 | 6.50E-01 | TRUE | 1.89E-04 |
| rs2398893 | 9 | 96758342 | 0.71 | G | A | 0.015 | 0.002 | 6.75E-16 | 0.002 | 0.026 | 9.34E-01 | TRUE | 1.83E-05 |
| rs2418135 | 9 | 113901309 | 0.52 | A | G | 0.015 | 0.002 | 5.31E-17 | -0.001 | 0.024 | 9.58E-01 | TRUE | 8.12E-06 |
| rs2444770 | 2 | 158503739 | 0.85 | T | C | 0.018 | 0.003 | 1.78E-13 | -0.026 | 0.037 | 4.87E-01 | TRUE | 8.03E-04 |
| rs244722 | 5 | 176534724 | 0.50 | C | A | -0.019 | 0.002 | 5.40E-21 | -0.008 | 0.024 | 7.43E-01 | TRUE | 1.74E-06 |
| rs2503100 | 6 | 100613915 | 0.83 | G | A | -0.025 | 0.002 | 4.86E-25 | 0.007 | 0.034 | 8.26E-01 | TRUE | 9.50E-08 |
| rs2506135 | 10 | 33465037 | 0.71 | C | T | 0.012 | 0.002 | 3.08E-09 | -0.003 | 0.028 | 9.06E-01 | TRUE | 2.18E-03 |
| rs2509963 | 11 | 62192931 | 0.26 | C | T | -0.017 | 0.002 | 9.36E-17 | 0.011 | 0.027 | 6.76E-01 | TRUE | 2.19E-05 |
| rs2526886 | 14 | 71359064 | 0.69 | T | G | 0.014 | 0.002 | 1.01E-10 | 0.021 | 0.026 | 4.04E-01 | TRUE | 4.35E-03 |
| rs2529411 | 7 | 50738482 | 0.64 | C | G | -0.011 | 0.002 | 2.83E-09 | -0.029 | 0.025 | 2.51E-01 | TRUE | 2.26E-02 |
| rs2545401 | 5 | 66348255 | 0.39 | C | G | 0.010 | 0.002 | 2.10E-08 | -0.001 | 0.025 | 9.67E-01 | TRUE | 2.22E-03 |
| rs2602674 | 2 | 225059163 | 0.54 | G | A | 0.011 | 0.002 | 1.87E-10 | -0.002 | 0.024 | 9.27E-01 | TRUE | 8.15E-04 |
| rs2701523 | 15 | 37384284 | 0.74 | G | A | 0.013 | 0.002 | 3.21E-10 | 0.020 | 0.032 | 5.17E-01 | TRUE | 3.25E-03 |
| rs2791550 | 1 | 219655369 | 0.25 | G | T | -0.037 | 0.002 | 3.35E-93 | 0.045 | 0.025 | 7.57E-02 | TRUE | 6.52E-22 |
| rs2791558 | 1 | 219583791 | 0.26 | T | C | 0.018 | 0.002 | 5.76E-18 | -0.032 | 0.029 | 2.67E-01 | TRUE | 2.62E-04 |
| rs2836179 | 21 | 39544159 | 0.41 | A | G | -0.017 | 0.002 | 7.16E-20 | -0.016 | 0.024 | 5.04E-01 | TRUE | 8.39E-06 |
| rs28451064 | 21 | 35593827 | 0.13 | A | G | 0.018 | 0.003 | 2.87E-09 | -0.014 | 0.033 | 6.61E-01 | TRUE | 4.03E-03 |
| rs28488569 | 5 | 180645353 | 0.88 | G | A | 0.017 | 0.003 | 2.37E-08 | 0.001 | 0.039 | 9.89E-01 | TRUE | 2.71E-03 |
| rs2854152 | 17 | 61986027 | 0.32 | G | A | 0.013 | 0.002 | 3.94E-12 | -0.006 | 0.025 | 7.93E-01 | TRUE | 3.64E-04 |
| rs2898237 | 21 | 36763769 | 0.69 | G | A | 0.012 | 0.002 | 7.47E-11 | 0.021 | 0.025 | 4.01E-01 | TRUE | 4.53E-03 |
| rs2925128 | 14 | 98362355 | 0.37 | T | C | -0.013 | 0.002 | 1.27E-11 | 0.062 | 0.024 | 8.99E-03 | TRUE | 1.19E-01 |
| rs2925979 | 16 | 81534790 | 0.30 | C | T | 0.027 | 0.002 | 7.33E-46 | -0.004 | 0.025 | 8.70E-01 | TRUE | 1.32E-13 |
| rs2970332 | 11 | 14360435 | 0.76 | A | G | 0.016 | 0.002 | 1.01E-13 | -0.062 | 0.025 | 1.36E-02 | TRUE | 5.21E-02 |
| rs2997447 | 1 | 26387423 | 0.21 | A | G | -0.014 | 0.002 | 1.38E-10 | -0.049 | 0.032 | 1.24E-01 | TRUE | 2.54E-02 |
| rs303084 | 4 | 124066948 | 0.78 | A | G | 0.019 | 0.002 | 2.29E-19 | 0.017 | 0.033 | 6.03E-01 | TRUE | 1.19E-05 |
| rs3098872 | 8 | 71932204 | 0.59 | T | G | -0.012 | 0.002 | 4.72E-11 | 0.034 | 0.024 | 1.58E-01 | TRUE | 1.70E-02 |
| rs3110697 | 7 | 45955029 | 0.43 | G | A | 0.012 | 0.002 | 1.08E-11 | -0.015 | 0.024 | 5.32E-01 | TRUE | 1.53E-03 |
| rs332105 | 2 | 119444229 | 0.55 | A | G | -0.014 | 0.002 | 4.93E-14 | -0.003 | 0.024 | 9.15E-01 | TRUE | 5.68E-05 |
| rs34005 | 5 | 141976945 | 0.43 | A | G | -0.013 | 0.002 | 7.04E-12 | 0.012 | 0.024 | 6.27E-01 | TRUE | 8.19E-04 |
| rs34312154 | 11 | 47470345 | 0.09 | A | G | 0.019 | 0.003 | 3.18E-10 | -0.039 | 0.030 | 1.93E-01 | TRUE | 2.18E-02 |
| rs34905952 | 3 | 138104635 | 0.14 | A | G | 0.021 | 0.003 | 1.23E-15 | 0.026 | 0.039 | 5.01E-01 | TRUE | 1.56E-04 |
| rs35643724 | 12 | 54387102 | 0.14 | A | G | -0.027 | 0.003 | 2.08E-21 | 0.017 | 0.057 | 7.60E-01 | TRUE | 8.69E-07 |
| rs36043408 | 1 | 203511492 | 0.51 | A | G | -0.012 | 0.002 | 4.07E-09 | -0.052 | 0.024 | 2.90E-02 | TRUE | 1.94E-01 |
| rs3731246 | 9 | 21971989 | 0.90 | G | C | -0.016 | 0.003 | 1.05E-08 | 0.001 | 0.043 | 9.83E-01 | TRUE | 2.13E-03 |
| rs3732083 | 2 | 207041053 | 0.58 | C | T | -0.011 | 0.002 | 1.74E-10 | -0.029 | 0.024 | 2.27E-01 | TRUE | 1.60E-02 |
| rs3747577 | 16 | 4415696 | 0.27 | G | C | 0.022 | 0.002 | 3.34E-26 | 0.028 | 0.027 | 3.00E-01 | TRUE | 7.73E-07 |
| rs3764002 | 12 | 108618630 | 0.26 | T | C | -0.018 | 0.002 | 1.86E-19 | -0.004 | 0.025 | 8.73E-01 | TRUE | 3.29E-06 |
| rs3786897 | 19 | 33893008 | 0.58 | G | A | -0.028 | 0.002 | 3.00E-58 | -0.047 | 0.024 | 5.32E-02 | TRUE | 4.21E-13 |
| rs3789615 | 1 | 114941326 | 0.43 | T | C | -0.012 | 0.002 | 7.33E-12 | 0.004 | 0.024 | 8.79E-01 | TRUE | 2.88E-04 |
| rs3792751 | 5 | 32773314 | 0.36 | T | C | 0.014 | 0.002 | 1.41E-14 | -0.016 | 0.025 | 5.12E-01 | TRUE | 4.08E-04 |
| rs3807947 | 7 | 20424889 | 0.61 | G | T | 0.014 | 0.002 | 7.54E-14 | 0.017 | 0.024 | 4.65E-01 | TRUE | 4.63E-04 |
| rs3848370 | 16 | 4360314 | 0.22 | C | G | -0.013 | 0.002 | 4.53E-09 | 0.032 | 0.026 | 2.27E-01 | TRUE | 3.11E-02 |
| rs3851294 | 1 | 205130413 | 0.10 | G | A | -0.026 | 0.003 | 9.03E-17 | -0.040 | 0.043 | 3.51E-01 | TRUE | 2.16E-04 |
| rs38902 | 7 | 116889718 | 0.54 | A | G | 0.014 | 0.002 | 1.60E-14 | 0.028 | 0.024 | 2.37E-01 | TRUE | 1.63E-03 |
| rs3913009 | 6 | 34397848 | 0.81 | T | C | 0.014 | 0.002 | 4.24E-10 | 0.058 | 0.029 | 4.24E-02 | TRUE | 8.29E-02 |
| rs3915053 | 4 | 125233408 | 0.24 | A | G | 0.014 | 0.002 | 1.60E-11 | -0.013 | 0.027 | 6.47E-01 | TRUE | 1.07E-03 |
| rs4130903 | 6 | 130370168 | 0.09 | T | C | 0.019 | 0.003 | 3.64E-09 | -0.038 | 0.052 | 4.69E-01 | TRUE | 1.02E-02 |
| rs41562 | 7 | 104845094 | 0.67 | C | T | -0.015 | 0.002 | 3.83E-16 | 0.006 | 0.026 | 8.05E-01 | TRUE | 2.97E-05 |
| rs4372913 | 2 | 114517748 | 0.79 | G | A | -0.016 | 0.002 | 2.65E-13 | 0.012 | 0.026 | 6.35E-01 | TRUE | 4.50E-04 |
| rs4420638 | 19 | 45422946 | 0.81 | G | A | 0.023 | 0.002 | 6.81E-22 | -0.064 | 0.027 | 1.74E-02 | TRUE | 1.58E-03 |
| rs4450871 | 4 | 4990298 | 0.55 | G | A | 0.017 | 0.002 | 3.23E-18 | -0.019 | 0.024 | 4.24E-01 | TRUE | 5.06E-05 |
| rs4474021 | 8 | 12632903 | 0.31 | T | G | 0.012 | 0.002 | 2.44E-10 | -0.023 | 0.025 | 3.48E-01 | TRUE | 1.02E-02 |
| rs4512391 | 8 | 126516988 | 0.62 | C | T | -0.016 | 0.002 | 5.49E-19 | 0.033 | 0.027 | 2.17E-01 | TRUE | 2.64E-04 |
| rs4558863 | 4 | 26079129 | 0.81 | C | T | -0.021 | 0.002 | 4.69E-19 | 0.061 | 0.035 | 8.06E-02 | TRUE | 1.11E-03 |
| rs4586926 | 4 | 15375527 | 0.64 | C | A | 0.012 | 0.002 | 1.32E-10 | -0.008 | 0.024 | 7.53E-01 | TRUE | 1.79E-03 |
| rs459193 | 5 | 55806751 | 0.25 | G | A | 0.028 | 0.002 | 3.99E-47 | -0.011 | 0.025 | 6.58E-01 | TRUE | 4.70E-14 |
| rs4612354 | 8 | 25916018 | 0.26 | C | T | 0.013 | 0.002 | 2.66E-08 | -0.034 | 0.026 | 2.00E-01 | TRUE | 4.61E-02 |
| rs4656912 | 1 | 160412880 | 0.27 | A | G | -0.015 | 0.002 | 8.77E-12 | 0.062 | 0.029 | 3.19E-02 | TRUE | 5.34E-02 |
| rs4686696 | 3 | 185516520 | 0.31 | A | G | 0.015 | 0.002 | 5.67E-17 | 0.041 | 0.026 | 1.16E-01 | TRUE | 1.17E-03 |
| rs4704389 | 5 | 76446103 | 0.41 | G | A | 0.012 | 0.002 | 1.74E-12 | -0.065 | 0.024 | 7.75E-03 | TRUE | 9.44E-02 |
| rs4714704 | 6 | 43825023 | 0.27 | A | G | -0.016 | 0.002 | 6.95E-16 | 0.093 | 0.025 | 2.02E-04 | TRUE | 2.00E-01 |
| rs473629 | 1 | 172141541 | 0.42 | A | T | -0.012 | 0.002 | 4.40E-11 | 0.016 | 0.025 | 5.16E-01 | TRUE | 2.45E-03 |
| rs4738141 | 8 | 72469742 | 0.74 | G | A | -0.026 | 0.002 | 9.58E-41 | 0.043 | 0.028 | 1.26E-01 | TRUE | 6.80E-09 |
| rs4751628 | 10 | 119315825 | 0.63 | T | G | -0.013 | 0.002 | 6.16E-13 | -0.015 | 0.024 | 5.35E-01 | TRUE | 5.14E-04 |
| rs4773173 | 13 | 111025118 | 0.67 | G | A | 0.015 | 0.002 | 4.23E-17 | -0.006 | 0.025 | 7.95E-01 | TRUE | 1.20E-05 |
| rs4795593 | 17 | 29613600 | 0.33 | C | G | -0.011 | 0.002 | 3.16E-09 | 0.028 | 0.025 | 2.52E-01 | TRUE | 2.42E-02 |
| rs4865444 | 4 | 1624125 | 0.63 | G | C | -0.011 | 0.002 | 4.35E-08 | -0.006 | 0.026 | 8.10E-01 | TRUE | 6.77E-03 |
| rs4868256 | 5 | 172748679 | 0.50 | T | C | 0.013 | 0.002 | 1.16E-11 | 0.016 | 0.024 | 5.20E-01 | TRUE | 1.92E-03 |
| rs4902632 | 14 | 69149428 | 0.18 | T | A | 0.020 | 0.002 | 2.39E-16 | 0.034 | 0.028 | 2.25E-01 | TRUE | 4.50E-04 |
| rs4925169 | 17 | 18222341 | 0.32 | A | G | -0.015 | 0.002 | 2.90E-13 | 0.024 | 0.025 | 3.35E-01 | TRUE | 1.60E-03 |
| rs4930724 | 12 | 124423817 | 0.66 | C | T | 0.037 | 0.002 | 2.46E-85 | -0.041 | 0.026 | 1.17E-01 | TRUE | 2.44E-20 |
| rs4964058 | 12 | 27522766 | 0.51 | A | G | 0.011 | 0.002 | 2.84E-10 | 0.041 | 0.024 | 8.66E-02 | TRUE | 5.07E-02 |
| rs541091 | 6 | 160770552 | 0.43 | A | G | -0.020 | 0.002 | 2.99E-26 | 0.002 | 0.024 | 9.41E-01 | TRUE | 2.13E-08 |
| rs55665241 | 14 | 68866527 | 0.40 | G | A | -0.012 | 0.002 | 1.02E-08 | -0.026 | 0.024 | 2.82E-01 | TRUE | 2.56E-02 |
| rs55747707 | 7 | 73037366 | 0.18 | A | G | -0.024 | 0.002 | 3.05E-25 | 0.039 | 0.031 | 2.02E-01 | TRUE | 9.61E-06 |
| rs55920843 | 2 | 158412701 | 0.99 | G | T | 0.086 | 0.009 | 2.68E-20 | -0.525 | 0.288 | 6.81E-02 | TRUE | 5.39E-04 |
| rs55957788 | 19 | 17207851 | 0.30 | A | G | 0.016 | 0.002 | 7.82E-14 | 0.021 | 0.028 | 4.59E-01 | TRUE | 8.56E-04 |
| rs56021223 | 14 | 52502227 | 0.04 | T | C | 0.030 | 0.005 | 1.85E-09 | -0.030 | 0.053 | 5.70E-01 | TRUE | 5.28E-03 |
| rs56185013 | 4 | 106160133 | 0.22 | A | G | -0.017 | 0.002 | 2.49E-12 | 0.038 | 0.029 | 1.88E-01 | TRUE | 7.43E-03 |
| rs56196860 | 12 | 2908330 | 0.03 | A | C | -0.036 | 0.006 | 6.45E-10 | -0.071 | 0.108 | 5.12E-01 | TRUE | 5.48E-03 |
| rs57462170 | 3 | 50239803 | 0.11 | A | G | 0.019 | 0.003 | 4.96E-09 | 0.090 | 0.033 | 6.82E-03 | TRUE | 3.86E-01 |
| rs577721086 | 6 | 127440047 | 0.95 | C | T | -0.166 | 0.005 | 1.00E-200 | 0.031 | 0.049 | 5.26E-01 | TRUE | 5.32E-77 |
| rs59888683 | 15 | 79049464 | 0.18 | T | C | 0.014 | 0.002 | 1.80E-09 | -0.020 | 0.029 | 4.86E-01 | TRUE | 8.96E-03 |
| rs6047259 | 20 | 21098319 | 0.62 | T | C | 0.010 | 0.002 | 4.32E-09 | 0.007 | 0.025 | 7.76E-01 | TRUE | 3.91E-03 |
| rs6068279 | 20 | 51234923 | 0.54 | T | G | 0.014 | 0.002 | 3.31E-15 | 0.024 | 0.024 | 3.15E-01 | TRUE | 6.23E-04 |
| rs615672 | 6 | 32574171 | 0.31 | C | G | -0.018 | 0.002 | 6.75E-21 | 0.055 | 0.025 | 2.43E-02 | TRUE | 1.37E-03 |
| rs62070804 | 17 | 27889643 | 0.02 | T | C | 0.047 | 0.008 | 4.36E-10 | -0.230 | 0.200 | 2.48E-01 | TRUE | 1.59E-02 |
| rs634869 | 6 | 139831757 | 0.42 | C | T | 0.023 | 0.002 | 2.60E-40 | -0.035 | 0.024 | 1.36E-01 | TRUE | 1.63E-09 |
| rs6427992 | 1 | 203102563 | 0.60 | C | G | 0.010 | 0.002 | 1.18E-08 | 0.019 | 0.025 | 4.37E-01 | TRUE | 1.35E-02 |
| rs6433219 | 2 | 171421125 | 0.27 | A | G | 0.016 | 0.002 | 2.84E-15 | 0.022 | 0.026 | 3.94E-01 | TRUE | 2.49E-04 |
| rs6434426 | 2 | 191726609 | 0.09 | A | G | -0.018 | 0.003 | 3.72E-08 | -0.047 | 0.064 | 4.64E-01 | TRUE | 1.64E-02 |
| rs6658424 | 1 | 200049302 | 0.28 | A | T | -0.014 | 0.002 | 2.88E-13 | -0.060 | 0.026 | 2.03E-02 | TRUE | 5.55E-02 |
| rs6664892 | 1 | 221363995 | 0.32 | T | C | 0.015 | 0.002 | 2.34E-12 | 0.003 | 0.026 | 9.14E-01 | TRUE | 1.79E-04 |
| rs6688233 | 1 | 9335745 | 0.24 | T | C | 0.021 | 0.002 | 9.64E-24 | 0.026 | 0.031 | 4.00E-01 | TRUE | 2.61E-06 |
| rs6719672 | 2 | 66234202 | 0.82 | G | A | -0.026 | 0.002 | 1.67E-29 | 0.008 | 0.029 | 7.83E-01 | TRUE | 2.66E-09 |
| rs6721459 | 2 | 67852836 | 0.53 | G | A | -0.018 | 0.002 | 4.08E-23 | -0.032 | 0.024 | 1.81E-01 | TRUE | 2.43E-05 |
| rs6795831 | 3 | 129341403 | 0.81 | C | A | 0.035 | 0.002 | 4.91E-53 | -0.008 | 0.032 | 7.93E-01 | TRUE | 1.70E-15 |
| rs6853254 | 4 | 26352363 | 0.35 | G | T | 0.017 | 0.002 | 4.02E-20 | 0.018 | 0.025 | 4.66E-01 | TRUE | 1.43E-05 |
| rs6861681 | 5 | 173362458 | 0.29 | A | G | 0.028 | 0.002 | 4.18E-49 | 0.011 | 0.024 | 6.39E-01 | TRUE | 1.07E-13 |
| rs6879860 | 5 | 157879652 | 0.41 | A | G | 0.011 | 0.002 | 4.78E-09 | -0.010 | 0.024 | 6.85E-01 | TRUE | 5.27E-03 |
| rs7070749 | 10 | 63882682 | 0.55 | A | G | 0.014 | 0.002 | 9.10E-12 | 0.025 | 0.024 | 2.94E-01 | TRUE | 5.20E-03 |
| rs7096078 | 10 | 3560644 | 0.57 | T | C | -0.011 | 0.002 | 4.43E-09 | 0.054 | 0.024 | 2.31E-02 | TRUE | 1.93E-01 |
| rs7102 | 16 | 11642242 | 0.65 | C | T | -0.012 | 0.002 | 1.38E-10 | 0.028 | 0.025 | 2.62E-01 | TRUE | 1.20E-02 |
| rs711076 | 12 | 77905207 | 0.68 | C | T | -0.012 | 0.002 | 1.49E-11 | -0.002 | 0.027 | 9.47E-01 | TRUE | 2.73E-04 |
| rs711869 | 2 | 13073967 | 0.56 | A | G | -0.019 | 0.002 | 1.96E-24 | -0.009 | 0.024 | 7.18E-01 | TRUE | 1.26E-07 |
| rs717795 | 1 | 2970464 | 0.80 | C | T | -0.020 | 0.002 | 6.86E-18 | -0.030 | 0.034 | 3.70E-01 | TRUE | 1.16E-04 |
| rs7178948 | 15 | 100119117 | 0.45 | G | T | 0.010 | 0.002 | 1.21E-08 | 0.003 | 0.024 | 8.84E-01 | TRUE | 2.96E-03 |
| rs7207618 | 17 | 73321687 | 0.24 | C | T | -0.014 | 0.002 | 1.25E-11 | 0.014 | 0.031 | 6.53E-01 | TRUE | 1.25E-03 |
| rs7221167 | 17 | 43933307 | 0.57 | C | T | -0.018 | 0.002 | 6.80E-19 | -0.015 | 0.025 | 5.46E-01 | TRUE | 1.02E-05 |
| rs7235010 | 18 | 20724810 | 0.77 | A | G | 0.019 | 0.002 | 5.65E-19 | -0.010 | 0.028 | 7.13E-01 | TRUE | 7.45E-06 |
| rs7242873 | 18 | 46885025 | 0.92 | G | A | -0.024 | 0.003 | 2.55E-14 | -0.006 | 0.032 | 8.61E-01 | TRUE | 8.05E-05 |
| rs727428 | 17 | 7537792 | 0.45 | C | T | 0.016 | 0.002 | 4.25E-18 | -0.031 | 0.024 | 2.05E-01 | TRUE | 2.33E-04 |
| rs72823022 | 17 | 40513732 | 0.92 | C | T | -0.026 | 0.004 | 3.81E-13 | 0.115 | 0.054 | 3.32E-02 | TRUE | 3.45E-02 |
| rs72999033 | 19 | 19366632 | 0.06 | T | C | 0.022 | 0.004 | 2.10E-08 | 0.082 | 0.049 | 9.13E-02 | TRUE | 1.06E-01 |
| rs73005768 | 6 | 152021049 | 0.96 | G | A | -0.028 | 0.005 | 8.04E-09 | 0.030 | 0.088 | 7.30E-01 | TRUE | 4.93E-03 |
| rs73111557 | 3 | 61523251 | 0.10 | T | C | -0.019 | 0.003 | 7.74E-09 | -0.025 | 0.035 | 4.77E-01 | TRUE | 1.13E-02 |
| rs7311622 | 12 | 98772975 | 0.45 | T | C | -0.010 | 0.002 | 1.07E-08 | -0.004 | 0.025 | 8.72E-01 | TRUE | 2.80E-03 |
| rs73164714 | 22 | 30491000 | 0.93 | C | T | -0.027 | 0.004 | 2.11E-11 | 0.020 | 0.083 | 8.11E-01 | TRUE | 7.20E-04 |
| rs73858966 | 3 | 99654183 | 0.08 | A | T | 0.024 | 0.004 | 2.46E-11 | -0.006 | 0.041 | 8.91E-01 | TRUE | 5.58E-04 |
| rs7395513 | 11 | 69262756 | 0.44 | A | G | -0.021 | 0.002 | 9.35E-27 | 0.024 | 0.024 | 3.15E-01 | TRUE | 7.80E-07 |
| rs740755 | 17 | 59496797 | 0.25 | C | G | 0.015 | 0.002 | 1.17E-13 | -0.006 | 0.030 | 8.30E-01 | TRUE | 1.54E-04 |
| rs747249 | 11 | 130271647 | 0.36 | G | A | 0.011 | 0.002 | 1.25E-09 | -0.038 | 0.025 | 1.27E-01 | TRUE | 4.97E-02 |
| rs74792869 | 17 | 45313545 | 0.99 | C | T | 0.049 | 0.009 | 2.68E-08 | -0.124 | 0.076 | 1.02E-01 | TRUE | 1.05E-01 |
| rs7492628 | 14 | 91547136 | 0.68 | G | C | -0.023 | 0.002 | 4.91E-32 | -0.015 | 0.027 | 5.78E-01 | TRUE | 1.45E-09 |
| rs750460 | 15 | 74241506 | 0.44 | A | G | -0.013 | 0.002 | 4.81E-13 | -0.014 | 0.024 | 5.66E-01 | TRUE | 6.20E-04 |
| rs75136602 | 9 | 93969277 | 0.08 | A | G | 0.022 | 0.004 | 1.14E-09 | 0.005 | 0.046 | 9.12E-01 | TRUE | 1.25E-03 |
| rs7589318 | 2 | 25378372 | 0.31 | A | G | -0.016 | 0.002 | 1.24E-18 | 0.031 | 0.026 | 2.33E-01 | TRUE | 1.12E-04 |
| rs7680787 | 4 | 122624065 | 0.65 | C | T | 0.014 | 0.002 | 1.17E-14 | 0.003 | 0.025 | 8.91E-01 | TRUE | 5.56E-05 |
| rs77097175 | 6 | 127472589 | 0.02 | A | C | 0.046 | 0.006 | 7.17E-15 | -0.065 | 0.083 | 4.31E-01 | TRUE | 4.32E-04 |
| rs7736177 | 5 | 55003944 | 0.69 | A | G | -0.017 | 0.002 | 6.30E-19 | -0.013 | 0.026 | 6.24E-01 | TRUE | 7.34E-06 |
| rs7744833 | 6 | 20581828 | 0.68 | G | A | 0.012 | 0.002 | 1.42E-09 | -0.030 | 0.027 | 2.62E-01 | TRUE | 1.70E-02 |
| rs7753502 | 6 | 127205732 | 0.50 | C | T | 0.021 | 0.002 | 1.29E-33 | -0.032 | 0.024 | 1.74E-01 | TRUE | 4.45E-08 |
| rs7767011 | 6 | 14591781 | 0.19 | G | A | -0.016 | 0.003 | 7.98E-11 | 0.007 | 0.029 | 8.17E-01 | TRUE | 8.65E-04 |
| rs780159 | 10 | 80907147 | 0.43 | G | A | -0.016 | 0.002 | 3.33E-20 | 0.020 | 0.024 | 4.00E-01 | TRUE | 2.81E-05 |
| rs78058190 | 2 | 219699999 | 0.05 | A | G | 0.036 | 0.005 | 4.27E-12 | 0.024 | 0.044 | 5.76E-01 | TRUE | 1.19E-03 |
| rs7828497 | 8 | 89339721 | 0.73 | G | T | -0.017 | 0.002 | 1.31E-14 | -0.066 | 0.027 | 1.66E-02 | TRUE | 2.69E-02 |
| rs7932891 | 11 | 10921512 | 0.30 | G | A | 0.014 | 0.002 | 1.39E-12 | 0.002 | 0.026 | 9.46E-01 | TRUE | 1.73E-04 |
| rs7942991 | 11 | 62337203 | 0.28 | T | C | 0.016 | 0.002 | 5.61E-18 | 0.010 | 0.029 | 7.39E-01 | TRUE | 1.48E-05 |
| rs7943309 | 11 | 116773653 | 0.03 | A | G | 0.027 | 0.005 | 9.59E-09 | -0.028 | 0.050 | 5.71E-01 | TRUE | 7.99E-03 |
| rs797486 | 13 | 51221618 | 0.89 | A | C | 0.037 | 0.003 | 4.38E-46 | 0.089 | 0.039 | 2.32E-02 | TRUE | 4.95E-09 |
| rs80076900 | 19 | 10056320 | 0.05 | T | C | 0.027 | 0.005 | 9.70E-09 | 0.024 | 0.045 | 5.94E-01 | TRUE | 7.65E-03 |
| rs801593 | 22 | 47195050 | 0.74 | C | G | -0.012 | 0.002 | 6.68E-10 | -0.032 | 0.025 | 1.96E-01 | TRUE | 2.53E-02 |
| rs8030277 | 15 | 94029153 | 0.68 | T | A | -0.013 | 0.002 | 5.97E-12 | 0.005 | 0.025 | 8.41E-01 | TRUE | 3.32E-04 |
| rs8054299 | 16 | 53498655 | 0.69 | G | C | 0.015 | 0.002 | 5.79E-14 | 0.016 | 0.027 | 5.44E-01 | TRUE | 2.68E-04 |
| rs805769 | 20 | 5667459 | 0.58 | C | T | -0.022 | 0.002 | 4.76E-27 | 0.029 | 0.024 | 2.39E-01 | TRUE | 1.41E-06 |
| rs8066985 | 17 | 68453345 | 0.49 | G | A | 0.023 | 0.002 | 2.03E-40 | 0.010 | 0.025 | 6.81E-01 | TRUE | 4.96E-12 |
| rs8103017 | 19 | 55999142 | 0.70 | G | C | -0.020 | 0.002 | 2.98E-19 | -0.058 | 0.026 | 2.73E-02 | TRUE | 3.06E-03 |
| rs8126001 | 20 | 62711459 | 0.49 | T | C | -0.016 | 0.002 | 1.60E-16 | -0.001 | 0.024 | 9.60E-01 | TRUE | 1.12E-05 |
| rs838145 | 19 | 49248730 | 0.55 | A | G | -0.012 | 0.002 | 2.44E-10 | -0.027 | 0.025 | 2.79E-01 | TRUE | 1.10E-02 |
| rs863750 | 12 | 124505444 | 0.58 | T | C | 0.037 | 0.002 | 4.17E-101 | 0.045 | 0.024 | 6.01E-02 | TRUE | 8.40E-25 |
| rs864745 | 7 | 28180556 | 0.51 | C | T | 0.018 | 0.002 | 2.06E-25 | -0.022 | 0.024 | 3.61E-01 | TRUE | 1.16E-06 |
| rs872401 | 4 | 13211708 | 0.66 | C | T | -0.011 | 0.002 | 1.32E-08 | 0.034 | 0.028 | 2.11E-01 | TRUE | 4.61E-02 |
| rs875868 | 1 | 65539253 | 0.57 | C | A | 0.012 | 0.002 | 6.88E-11 | -0.009 | 0.025 | 7.13E-01 | TRUE | 1.28E-03 |
| rs8887 | 19 | 4502201 | 0.43 | C | T | -0.011 | 0.002 | 3.22E-09 | 0.062 | 0.024 | 1.02E-02 | TRUE | 2.66E-01 |
| rs905938 | 1 | 154991389 | 0.72 | C | T | 0.024 | 0.002 | 1.35E-35 | 0.014 | 0.028 | 6.09E-01 | TRUE | 8.23E-10 |
| rs910071 | 20 | 39213710 | 0.32 | T | C | -0.017 | 0.002 | 6.27E-19 | 0.001 | 0.027 | 9.84E-01 | TRUE | 1.23E-06 |
| rs9327468 | 5 | 127441861 | 0.77 | A | C | 0.015 | 0.002 | 4.15E-12 | 0.031 | 0.033 | 3.54E-01 | TRUE | 3.14E-03 |
| rs9369409 | 6 | 43346462 | 0.42 | G | C | 0.015 | 0.002 | 9.37E-15 | 0.036 | 0.025 | 1.45E-01 | TRUE | 3.30E-03 |
| rs9415106 | 10 | 77295957 | 0.71 | A | G | 0.014 | 0.002 | 9.78E-13 | 0.027 | 0.025 | 2.92E-01 | TRUE | 3.14E-03 |
| rs9425301 | 1 | 172363815 | 0.36 | C | A | 0.024 | 0.002 | 2.26E-40 | 0.015 | 0.024 | 5.36E-01 | TRUE | 5.05E-11 |
| rs951252 | 4 | 145825151 | 0.45 | G | A | -0.019 | 0.002 | 3.44E-27 | 0.014 | 0.024 | 5.53E-01 | TRUE | 7.22E-08 |
| rs9557378 | 13 | 100747566 | 0.26 | A | G | 0.011 | 0.002 | 2.50E-08 | -0.027 | 0.025 | 2.87E-01 | TRUE | 3.09E-02 |
| rs9601364 | 13 | 80952100 | 0.79 | C | T | 0.014 | 0.002 | 2.36E-08 | 0.013 | 0.030 | 6.69E-01 | TRUE | 6.84E-03 |
| rs9644033 | 8 | 23610639 | 0.75 | T | A | 0.022 | 0.002 | 3.40E-26 | 0.054 | 0.029 | 6.43E-02 | TRUE | 3.17E-05 |
| rs9678859 | 2 | 100288478 | 0.18 | G | A | 0.019 | 0.002 | 7.97E-16 | -0.035 | 0.035 | 3.23E-01 | TRUE | 3.32E-04 |
| rs9792401 | 8 | 25789840 | 0.34 | G | T | -0.012 | 0.002 | 5.29E-10 | 0.013 | 0.025 | 6.02E-01 | TRUE | 4.19E-03 |
| rs990211 | 3 | 48721040 | 0.21 | G | A | -0.021 | 0.002 | 3.06E-21 | -0.063 | 0.031 | 3.97E-02 | TRUE | 8.57E-04 |
| rs998584 | 6 | 43757896 | 0.48 | A | C | 0.049 | 0.002 | 1.22E-170 | 0.055 | 0.024 | 2.04E-02 | TRUE | 9.25E-42 |
| rs998749 | 3 | 168972802 | 0.50 | G | A | 0.014 | 0.002 | 6.23E-16 | 0.006 | 0.024 | 8.01E-01 | TRUE | 2.56E-05 |
| **childhood BMI** | | | | | | | | | | | | | |
| rs114670539 | 2 | 207064335 | 0.05 | T | C | 0.099 | 0.018 | 3.16E-08 | 0.100 | 0.072 | 1.68E-01 | TRUE | 2.49E-06 |
| rs11676272 | 2 | 25141538 | 0.47 | A | G | -0.075 | 0.008 | 2.37E-21 | -0.058 | 0.024 | 1.64E-02 | TRUE | 7.62E-16 |
| rs12042908 | 1 | 74997762 | 0.45 | A | G | 0.059 | 0.008 | 2.77E-14 | 0.007 | 0.024 | 7.66E-01 | TRUE | 2.09E-12 |
| rs12641981 | 4 | 45179883 | 0.43 | T | C | 0.044 | 0.008 | 4.19E-08 | 0.045 | 0.024 | 5.89E-02 | TRUE | 6.88E-06 |
| rs13107325 | 4 | 103188709 | 0.07 | T | C | 0.095 | 0.017 | 3.51E-08 | 0.075 | 0.102 | 4.62E-01 | TRUE | 9.25E-07 |
| rs17817449 | 16 | 53813367 | 0.40 | T | G | -0.068 | 0.008 | 1.69E-17 | -0.081 | 0.024 | 8.24E-04 | TRUE | 8.14E-12 |
| rs41279738 | 1 | 110082551 | 0.94 | T | G | -0.120 | 0.021 | 1.30E-08 | -0.129 | 0.050 | 9.66E-03 | TRUE | 9.64E-06 |
| rs4477562 | 13 | 54104968 | 0.13 | T | C | 0.080 | 0.011 | 8.29E-13 | 0.029 | 0.034 | 3.97E-01 | TRUE | 1.40E-10 |
| rs543874 | 1 | 177889480 | 0.20 | A | G | -0.079 | 0.010 | 1.62E-15 | -0.078 | 0.030 | 9.86E-03 | TRUE | 3.89E-11 |
| rs56133711 | 11 | 27723334 | 0.25 | A | G | 0.057 | 0.009 | 2.00E-10 | 0.052 | 0.027 | 6.03E-02 | TRUE | 1.12E-07 |
| rs571312 | 18 | 57839769 | 0.23 | A | C | 0.059 | 0.009 | 2.00E-10 | 0.103 | 0.030 | 5.45E-04 | TRUE | 2.12E-06 |
| rs61765651 | 1 | 72754314 | 0.83 | T | C | -0.058 | 0.010 | 9.50E-09 | -0.013 | 0.034 | 7.05E-01 | TRUE | 1.65E-07 |
| rs62500888 | 8 | 28061823 | 0.57 | A | G | 0.047 | 0.008 | 6.91E-10 | -0.001 | 0.025 | 9.59E-01 | TRUE | 6.51E-09 |
| rs7138803 | 12 | 50247468 | 0.38 | A | G | 0.073 | 0.008 | 7.12E-20 | 0.028 | 0.025 | 2.49E-01 | TRUE | 4.03E-16 |
| rs7199285 | 16 | 19980931 | 0.17 | T | C | -0.065 | 0.010 | 1.34E-10 | -0.025 | 0.032 | 4.30E-01 | TRUE | 1.01E-08 |
| rs939584 | 2 | 621558 | 0.83 | T | C | 0.107 | 0.010 | 8.85E-26 | 0.039 | 0.032 | 2.16E-01 | TRUE | 7.85E-21 |
| ^※^Only lists SNPs of the exposures after matching and harmonizing with the outcomes.  Abbreviations: BMI, body mass index; WHR, waist-to-hip ratio; WHR_adj_BMI, waist-to-hip ratio adjusted for body mass index; diabetic nephropathy . | | | | | | | | | | | | | |
